# Discovery of a Peptoid-Based Nanoparticle Platform for Therapeutic mRNA Delivery via Diverse Library Clustering and Structural Parametrization

**DOI:** 10.1021/acsnano.4c05513

**Published:** 2024-08-06

**Authors:** Elizabeth R. Webster, Nicole E. Peck, Juan Diego Echeverri, Shima Gholizadeh, Wei-Lun Tang, Rinette Woo, Anushtha Sharma, Weiqun Liu, Chris S. Rae, Adrienne Sallets, Gowrisudha Adusumilli, Kannan Gunasekaran, Ole A. W. Haabeth, Meredith Leong, Ronald N. Zuckermann, Samuel Deutsch, Colin J. McKinlay

**Affiliations:** † Nutcracker Therapeutics, 5980 Horton Street Suite 350, Emeryville, California 94608, United States; ‡ Molecular Foundry, Lawrence Berkeley National Laboratory, Berkeley, California 94720, United States

**Keywords:** peptoid, mRNA delivery, lipid nanoparticle, design of experiments, nucleic acid delivery, high-throughput screening

## Abstract

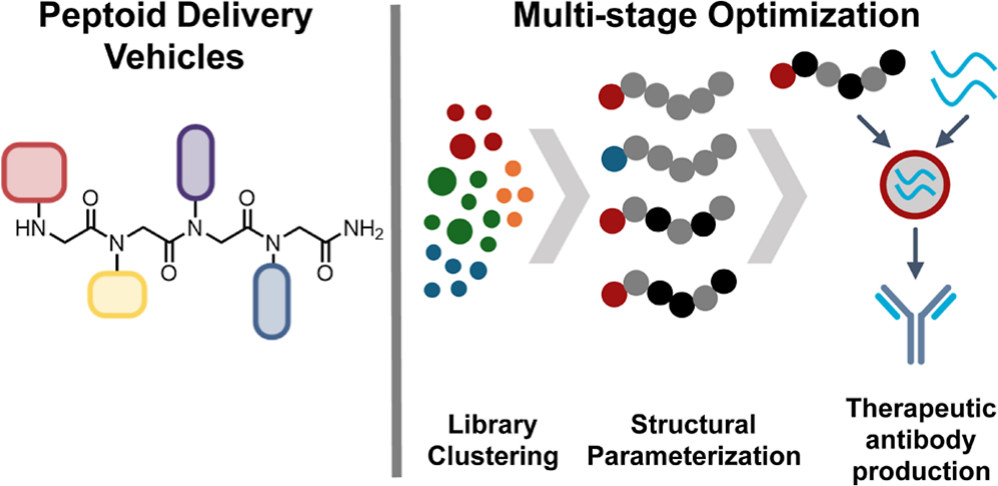

Nanoparticle-mediated mRNA delivery has emerged as a promising therapeutic modality, but its growth is still limited by the discovery and optimization of effective and well-tolerated delivery strategies. Lipid nanoparticles containing charged or ionizable lipids are an emerging standard for in vivo mRNA delivery, so creating facile, tunable strategies to synthesize these key lipid-like molecules is essential to advance the field. Here, we generate a library of N-substituted glycine oligomers, peptoids, and undertake a multistage down-selection process to identify lead candidate peptoids as the ionizable component in our Nutshell nanoparticle platform. First, we identify a promising peptoid structural motif by clustering a library of >200 molecules based on predicted physical properties and evaluate members of each cluster for reporter gene expression in vivo. Then, the lead peptoid motif is optimized using design of experiments methodology to explore variations on the charged and lipophilic portions of the peptoid, facilitating the discovery of trends between structural elements and nanoparticle properties. We further demonstrate that peptoid-based Nutshells leads to expression of therapeutically relevant levels of an anti-respiratory syncytial virus antibody in mice with minimal tolerability concerns or induced immune responses compared to benchmark ionizable lipid, DLin-MC3-DMA. Through this work, we present peptoid-based nanoparticles as a tunable delivery platform that can be optimized toward a range of therapeutic programs.

The therapeutic use of messenger-RNA based drugs has proven potential to transform many areas of human health. The FDA approval of mRNA vaccines against SARS-COV2 employing lipid nanoparticle (LNP) delivery technology demonstrates the significant progress in this field.^1−3^ The extension of mRNA drugs to applications beyond vaccines, such as protein replacement or immune-oncology therapies generating circulating antibodies, necessitates robust delivery strategies and platforms.^4,5^ In LNPs, mRNA molecules are encapsulated in a combination of a cationic or ionizable lipid, helper lipids such as cholesterol and phospholipids, and a shielding lipid such as poly(ethylene glycol) (PEG).^6,7^ While many reports have shown that tuning ionizable lipid properties impacts expression and tissue selectivity of delivery,^7−9^ understanding how the structure of individual components impacts nanoparticle function is still an ongoing effort.^10,11^ As mRNA is applied to a wider range of applications, including those that rely on IV delivery, the ability to modulate the properties of ionizable lipids in particular has become a key focus of the field. Recent notable advances in ionizable lipid design have included alkyne,^12^ beta-amido carbonate,^13^ phosphonate,^14^ and disulfide^15−18^ containing lipids, among many others.^19−21^ Nevertheless, generation of alternative ionizable lipids is often limited by the significant synthetic effort and resources needed to expand lipid libraries, so many of the most successful reports are those that utilize combinatorial or high-throughput libraries for candidate discovery.^16,17,22−24^ Here, we present a platform of mRNA delivery vehicles termed Nutshells, which utilize peptoids as the ionizable lipid component to enable diverse and systematic modulation of the peptoid base structure, thus tuning the biological properties of formulated particles.

Peptoids, or N-substituted alpha-amino acid oligomers, are a class of biomacromolecules structurally related to the commonly known peptides, but which incorporate side chain functionality on the amide nitrogen rather than α carbon, allowing for a significantly expanded set of monomer functionality, modular synthesis, and distinct structural properties.^25^ Foundational work by Zuckermann demonstrated that this class of materials can be expediently synthesized in high yield and fidelity on a solid support using the submonomer approach wherein repeating cycles of acylation using bromoacetic acid and nucleophilic displacement by primary amines grow these materials in a stepwise, programmable fashion.^26−28^ Peptoids have been successfully used in many applications including antibiofouling^29^ and antibacterial agents,^30,31^ drug delivery,^32^ antifreeze additives in tissue storage,^33,34^ and even as complexing agents for nucleic acids^35^ or shielding lipids for LNPs,^36−38^ but never as the ionizable component for mRNA delivery. Given the tunable nature of the synthesis and biomimetic properties, we hypothesized that peptoids would be an ideal candidate for the ionizable component of LNPs, creating effective and well-tolerated delivery vehicles. This sequence flexibility can enable the discovery of lipid-like materials with properties that escape previous paradigms of ionizable lipid structure, leading to enhanced delivery efficacy and variable biodistribution. Using solid-phase synthesis and the modular nature of the peptoid structure, we have assembled and evaluated a library of over 500 peptoid candidates for mRNA delivery, over 200 of which are highlighted in this work. Diverse library generation for delivery vehicle screening has been used by others including Wagner and co-workers for polyplex-driven delivery of nucleic acids.^39−42^ Here, we formulate this diverse peptoid library with mRNA, along with DSPC, Cholesterol, and DMG-PEG2000 to generate Nutshell particles, which can serve as a versatile platform for developing mRNA therapeutics.

Through multiple rounds of screening, we have identified structural classes of peptoids that can be used for diverse delivery applications including to the lung, spleen, and liver. Then, as one demonstration of the potential of peptoid-based Nutshell particles for tailored therapeutic modalities, we further optimized liver-targeted peptoid structures to maximize hepatic expression of mRNA-encoded antibodies.

Our optimization process utilizes a series of in silico and in vivo down-selection and structural refinement steps to identify lead structural classes which are then chemically optimized for enhanced mRNA delivery (Figure 1A). First, in silico physical property predictions and hierarchical clustering were used to group our entire peptoid library into clusters with related attributes. A subset of candidates from each of these clusters was then formulated with mRNA and evaluated for firefly luciferase expression (Fluc) in mice. In a second phase, the top-performing cluster(s) were selected as model compounds for structural refinement, in which we screened a panel of ionizable groups, followed by an optimization of the lipid features. This approach takes advantage of the modularity of peptoid structure and the fact that interactions between lipid and cationic portions of ionizable lipids are typically minimal.^22,43,44^ Following this approach, we demonstrate how the Nutshell platform can be optimized for the systemic delivery of mRNAs encoding a therapeutic antibody against respiratory syncytial virus (RSV) and compare expression levels and tolerability to the previously developed ionizable lipid, DLin-MC3-DMA.^45^

**Figure 1:**
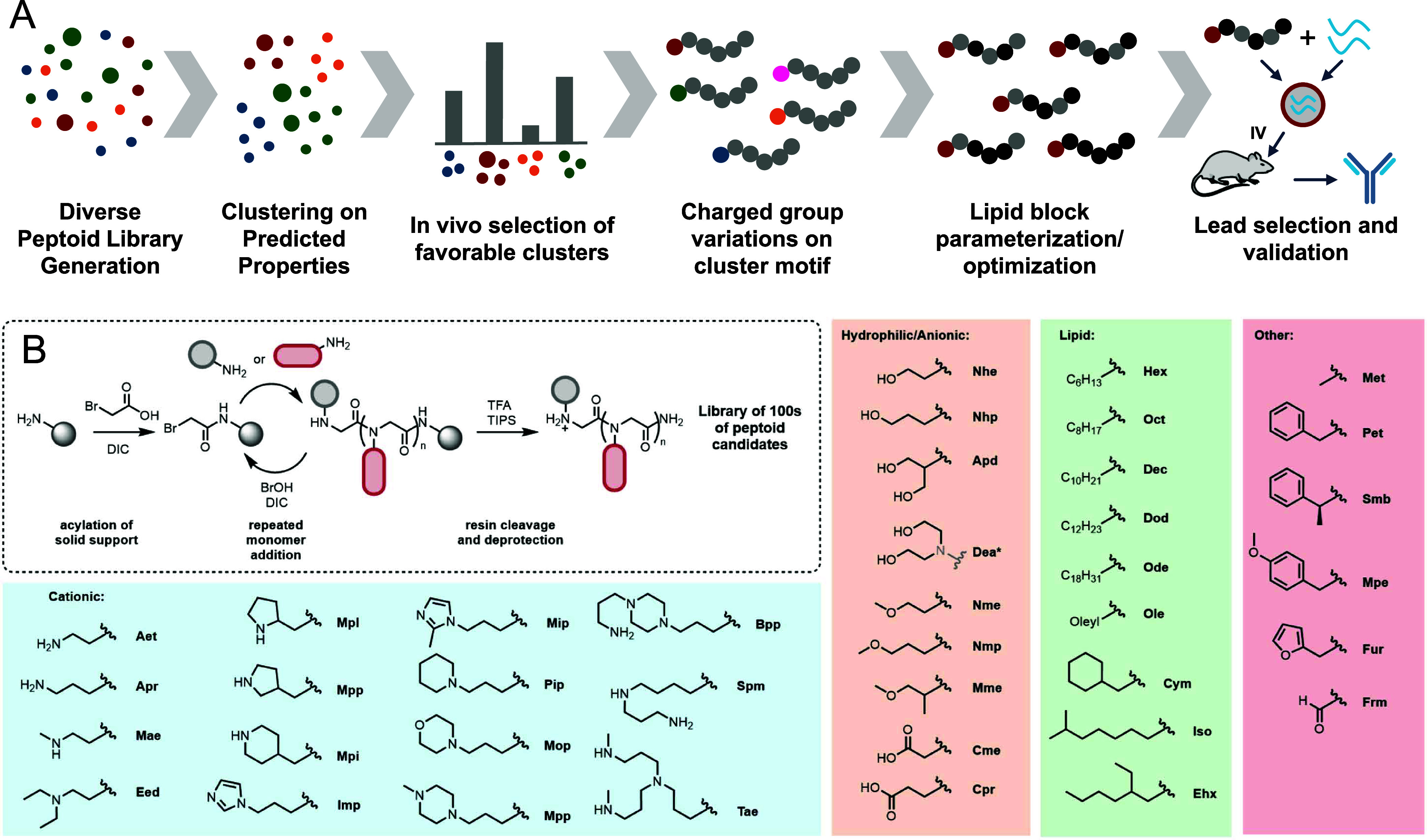
Facile synthetic processes enable the generation of a diverse library of peptoid candidates for mRNA delivery. (A) Outline of methodology used here to identify top-performing peptoid candidates starting with a diverse peptoid library, and then to optimize those candidates through a multistage structural parametrization of charged group and lipid monomer structure. (B) Submonomer synthesis method conducted on a solid support that produces peptoid candidates with high yield and sequence fidelity. The pool of monomer functionalities used in library construction is shown categorized by monomer type, along with three-letter designations used in sequences.

## Results and Discussion

We have assembled a large and diverse library of 221 peptoid molecules as potential candidates for systemic mRNA delivery using the submonomer approach (Figure 1B, sequences available in Table S1). This library contains peptoids of 2 to 30 residues in length and 39 different individual monomer functionalities (Figure 1B). Monomer functionalities encompass cationic, anionic, hydrophilic, and lipid groups.

**Figure 2:**
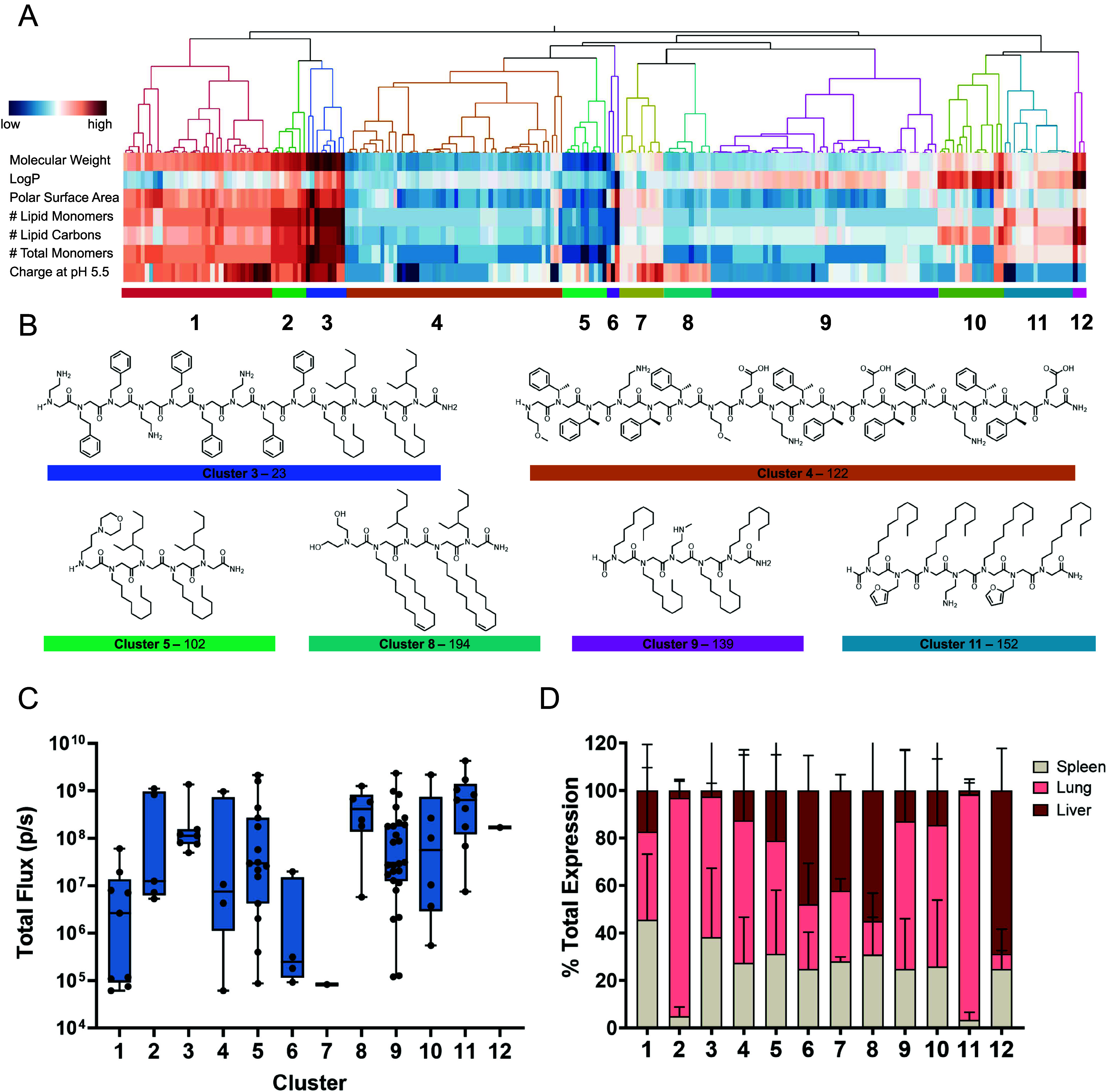
Clustering analysis of a diverse peptoid library to identify high-performing sequence motifs used in later optimization steps. (A) Hierarchical clustering of peptoid candidates based on predicted physical properties to group peptoids into 12 distinct families. (B) Representative structures from 6 of the 12 families highlight chemical distinctiveness. (C) Expression of firefly luciferase (Fluc) in Balb/c mice following injection of 0.125–0.25 mg/kg mRNA, and imaged after 6 h. Values are grouped by cluster, with each point representing the mean flux (*n* = 3) of individual cluster members. (D) Average organ distribution of Fluc signal for each cluster.

Exhaustively screening this entire library for in vivo expression would incur unnecessarily high animal usage and material costs, so we decided to first group peptoids with similar physical properties, thereby limiting the need to screen chemically redundant candidates. This was accomplished by using a Ward’s hierarchical clustering method to categorize peptoids into 12 different clusters based on the following in-silico predicted parameters: (1) molecular weight, (2) Log *P*, (3) total polar surface area, (4) predicted molecular charge at pH 5.5 and 7, (5) total number of monomers, (6) total number of hydrophobic or “lipid” monomers, and (7) total number of carbons contained on lipid monomers (Figure 2A). We hypothesized that this would adequately differentiate classes of peptoids with different properties and behaviors. Some selected examples of peptoid structures of cluster members are shown in Figure 2B, and a complete table of sequences and cluster memberships is presented in Table S1. Structural distinctiveness between clusters can be seen, for example, where cluster 3 contains peptoids with relatively high values for all parameters, with an example structure **23** containing a repeating cationic motif of aminoethyl and phenethyl side chains, followed by 4 lipid monomers (Figure 2B and Table S2). In contrast, cluster 5 contains structures with much lower molecular weights and numbers of charges, as exemplified by peptoid **102**, which contains a single charged morpholinone monomer and 4 lipid monomers. With clusters identified, peptoids were formulated into particles encapsulating Fluc mRNA using a microfluidic process as described in the methods section. In total, 93 peptoids were evaluated in vivo in Balb/c mice spread across the 12 clusters. The resulting total Fluc expression and percent localization in liver, lung, and spleen for each cluster are shown in Figure 2C,D.

The highest overall expression for all peptoids tested was observed in clusters 8 and 11 (Figure 2C). Both of these clusters have intermediate molecular weights, log *P* values, and numbers of total lipid monomers in the library. Interestingly, the localization patterns between the two were quite different, with cluster 8 primarily expressing in the liver, and cluster 11 nearly completely localized in the lung (Figures 2D and S2). We hypothesize that the distinct p*K*_a_ of the charged groups in these clusters drives differences in particle charge and localization. To better understand the connection between localization and particle charge, we collected zeta potential on representative peptoids from each cluster and saw differentiation among clusters (Figure S3). Cluster 8 contains lower p*K*_a_ amino groups such as diethanolamine (Dea) and 2-amino-1,3-propanediol (Apd) compared to the much more basic charged groups in cluster 11 (e.g., primary and secondary amines like Aet, Apr, and Mae). High particle charge is known to direct LNP cargos to the lung, while moderate surface charges often localize in the liver.^46,47^ The highest spleen expression was observed for cluster 3, which had significantly higher molecular weights and the largest number of total lipid monomers of any cluster. Interestingly, based on the hierarchy of clustering, cluster 3 splits from cluster 11 almost immediately, suggesting that they are the most chemically distinct from each other (Figure 2A) which is also reflected in their expression patterns. Some clusters, such as 6 and 7, had generally low expression across all organs. Of these, cluster 6 had a negative log *P*, confirming that significant lipophilicity is necessary for peptoids to act as an ionizable group. Taken together, these results suggest that peptoids as a class have utility across many different applications in mRNA delivery and different peptoid structural attributes can lead to distinct expression patterns. We selected cluster 8 for further optimization of a delivery vehicle to express mRNA-encoded therapeutic antibodies as a proof-of-principle of the therapeutic potential of this platform since it showed the second highest expression of any cluster, and had the greatest liver selectivity and thus avoided potential tolerability concerns of lung-based expression.^46,48,49^

**Figure 3:**
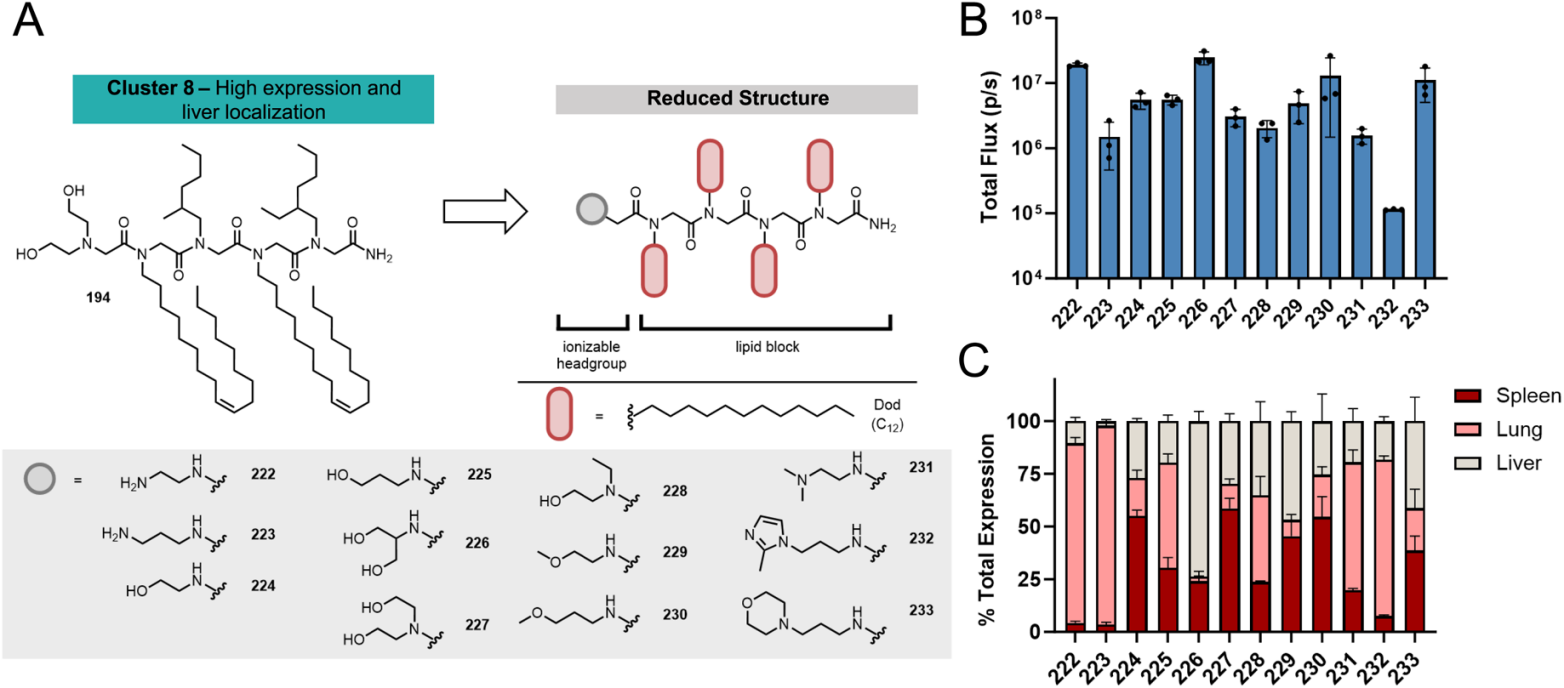
Optimization of the cationic portion of peptoid-based lipids. (A) Peptoid motif from Cluster 8 is reduced into a generalized structure and used to optimize charged headgroups for those with the highest liver selectivity and expression using a conserved lipid block. (B) Total luciferase expression in Balb/c mice 6 h after 0.125 mg/kg dose. (C) % total expression of in vivo luciferase expression among major organs: liver, lung, and spleen.

With a base motif selected, the next phase of structural refinement focused on the optimization of the cationic N-terminus of the peptoid molecules. Lead structures from cluster 8 with strong luciferase expression in the liver were selected as the starting point for optimization. The identity of the basic group used in an ionizable lipid significantly drives the particle p*K*_a_, which is correlated with efficacy and specificity of nanoparticle expression.^50^ Particle p*K*_a_’s of 6–6.5 generally show the highest efficacy in selective liver expression.^45,51,52^ However, it was unknown how a peptoid’s molecular p*K*_a_ would correlate to the observed p*K*_a_ once formulated in a particle; therefore, a variety of headgroup amines with varying basicity were selected. For this screen, a lipid block containing 4 dodecyl lipid (Dod) monomers was selected, and 12 peptoids containing these groups were synthesized (Figure 3A and Supporting Information Synthetic Characterization Data).

Peptoid headgroup variations were then formulated into Nutshell particles containing Fluc mRNA for in vivo testing, and particle properties including size, percent encapsulated RNA, and hemolysis were characterized (Table S3). In vivo expression with varied cationic groups was evaluated using Fluc expression in Balb/c mice 6 h after IV dose (Figure 3B). This panel of headgroup-modified peptoids showed significant differences in both total expression levels and organ selectivity, highlighting the importance of screening wide chemical diversity in the ionizable portion of the peptoid. The aminoethyl (Aet, **222**) and aminopropyl (Apr, **223**) charged monomers both show preferential expression in lung (Figure 3C), potentially due to the high basicity of the pendant primary amine, as groups with higher p*K*_a_’s are known to accumulate in the lung.^53,54^ Interestingly, the methoxyethyl (Nme, **229**) and methoxypropyl (Nmp, **230**) monomers shifted selectivity toward spleen from liver. While spleen and lung selectivity merit further investigation and will likely enable additional therapeutic applications, we chose to focus on liver-selective peptoids to demonstrate the in vivo expression of secreted antibody targets. The 1,3 diol (Apd, **226**) terminal monomer shows the highest liver expression and selectivity among the tested peptoids and was chosen for further lipid block optimization.

**Figure 4:**
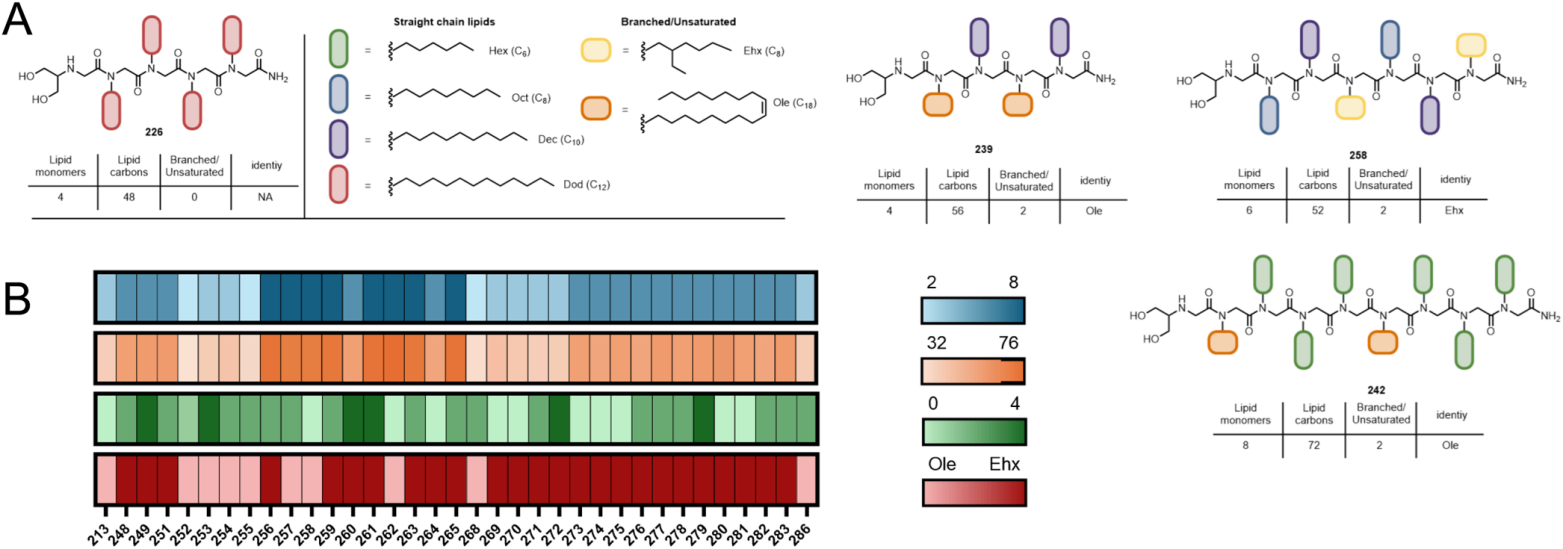
Parameterization strategy for representing peptoid lipid block variations. (A) Pool of lipid monomers used in variations and example structures highlighting the 4 parameters varied: Total number of lipid monomers, total lipid carbons, number of branched or unsaturated monomers, and identity of the branched/unsaturated monomer. Several examples of peptoid structures are shown. (B) Graphical representation of the design space surveyed by DOE-generated sequences varying each of the 4 aforementioned parameters. Each point represents a peptoid sequence that was synthesized and evaluated for mRNA delivery.

We hypothesized that modification to the lipophilic portion of the peptoid while keeping the leading Apd headgroup would allow for further optimization of the overall expression while maintaining or increasing liver selectivity. For this optimization, 6 different aliphatic monomers were selected ranging in length from C6 to C12, including the unsaturated oleyl (Ole) and branched 2-ethylhexyl (Ehx) lipids (Figure 4A). Even with this relatively small monomer pool, there are 55,944 peptoids designs of length 3–8 that are possible, so a systematic approach to evaluating this large structure space using design of experiments (DOE) methodology was employed. To allow chemical structure information to be accurately input into a DOE model, we devised a method of parametrizing properties of the lipid block using 4 key factors likely to impact LNP performance: (1) the total number of lipid-containing monomers in the structure, (2) the total number of carbon atoms contained on all lipid side chains,^55^ (3) the number of branched/unsaturated lipid monomers used,^56^ and (4) the identity of the branched/unsaturated monomer used.^57,58^ Each synthesized peptoid could be represented by a combination of these four factors, and the resulting performance data was fit to a multivariable model to separate the contribution of each factor to mRNA delivery performance. This method of parametrization does not attempt to capture the order of monomers within the sequence, so peptoids were designed to have alternating and repeating or symmetric motifs wherever possible. Further expansion of this methodology to sequence and order variations will be the subject of further investigation. Using this parametrization methodology, a multivariate DOE model was employed to generate 34 candidate peptoids structures spanning the defined parameter space (Figure 4B, Table S4).

Peptoid designs were synthesized based on DOE and target masses verified by liquid chromatography–mass spectrometry (LC-MS) (Supporting Information Synthetic Characterization). In line with our expectation, changing both the number of lipid monomers and total lipid carbons had a significant impact on the hydrophobicity of peptoid molecules as measured by high-performance liquid chromatography (HPLC) retention time (Figure S4). When fit to a multiparameter model, both the calculated log *P* and empirical reversed-phase HPLC retention time increase as the number of lipid carbons increased, as would be expected for a molecule with increasing lipid content. Additionally, as the number of lipid monomers increases (at a similar number of total lipid carbons), the predicted log *P* and retention time decrease, likely due to the increased contribution of hydrophilic amide backbone toward the overall molecule polarity. Taken together, these parameters result in a library of peptoid lipids with varying physical characteristics to optimize delivery and release when formulated into nanoparticles with mRNA cargos.

**Figure 5:**
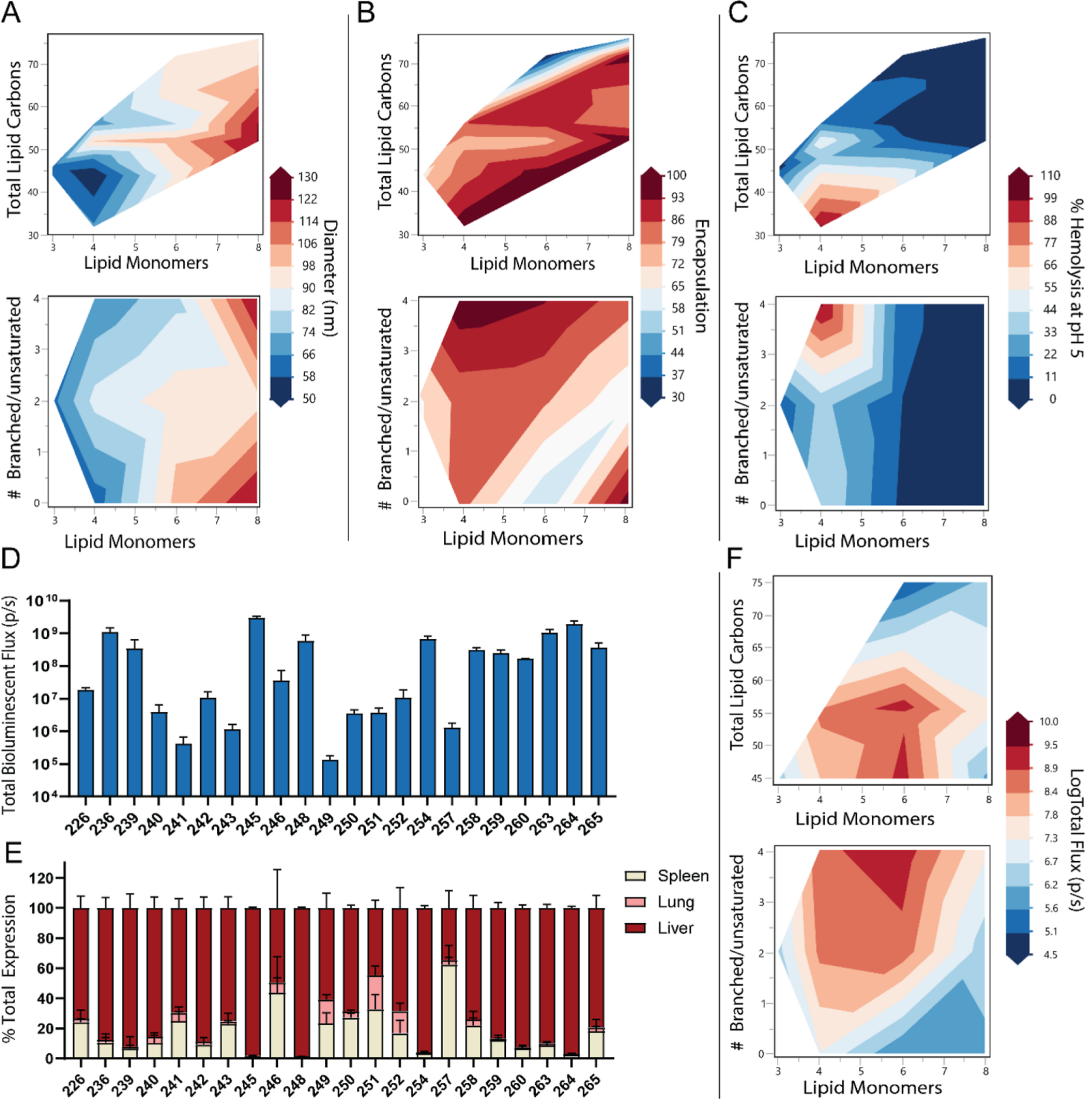
Impact of lipid block variations on the physical particle properties and in vivo expression. Multiparametric modeling contour plots were used to show how lipid parameters impact (A) diameter, (B) % encapsulated mRNA, and (C) hemolysis at pH 5. (D) Total Fluc bioluminescence in balb/c liver from lipid variations (E) distribution of bioluminescence among major organs from peptoid lipid block variations. (F) Contour plot of the impact of lipid parameters on in vivo luciferase expression.

Nutshell particles formulated using Apd lipid block variation peptoids were first evaluated for their physical properties, and then for their in vivo expression of luciferase mRNA (Figure 5). To understand the contribution of lipid-block properties to peptoid nanoparticle efficacy, experimental data for particle size, percent encapsulated mRNA, zeta potential, and p*K*_a_ were collected (Table S5). Overall, we found that monomer length and total carbon number have significant impacts on physical properties of particles. Encouragingly, the peptoids with the most lipid monomers and maximum total carbon number are not top performing, suggesting the range of monomers lengths we tested in this study is sufficient to identify an ideal balance of lipid character of the monomer side chains to the hydrophilicity of the peptoid backbone. Particle size was found to not strongly correlate with any single factor, except that particles were significantly larger when the oleyl (Ole) monomer was used relative to 2-ethylhexyl (Ehx) which might suggest differences in particle packing based on recent reports using small-angle X-ray scattering to characterize internal particle structure (Figure 5A).^59,60^ Percent of encapsulated mRNA depended on both the total number of lipid carbons on the peptoids and the type of branched/unsaturated monomer used, with maximal encapsulation occurring around 55 total lipid carbons, and using the 2-ethylhexyl (Ehx) monomer (Figure 5B). Consistent with a previous report of lipid length and branching impact LNP charge, we see particle p*K*_a_ varies from pH 5–7 with higher monomer and high total carbon count leading to lower particle p*K*_a_.^57^ Additionally, we see that increasing the number of branched monomers leads to a slightly lower p*K*_a_ in Nutshells even with similar headgroup p*K*_a_ (Figure S5). While traditional LNP ionizable lipids achieve the optimal particle p*K*_a_ of 6–6.5 by utilizing a tertiary or secondary amine with predicted p*K*_a_ of 9.5–10.5,^55^ Apd peptoids have predicted p*K*_a_’s of approximately 5.5–5.7, which are similar to their measured particle p*K*_a_’s of 5–7, indicating a lack of p*K*_a_ shift upon formulation into a LNP complex. This could be due to several factors including the hydrophilicity of the peptoid backbone providing an environment more favorable to amine protonation or the packing dynamics of the peptoid within the particle and will be the focus of further investigation. Fusogenicity, or the ability of the LNP to fuse with endosomal membranes and escape degradation, has also been shown to vary with lipid structure for LNPs.^43,61^ Hemolysis of red blood cells at neutral and acidic pH was used as a measure of fusogenicity.^22,56^ At low pH which is most predictive of membrane interactions in the endosome, increasing branched/unsaturated groups leads to higher hemolysis at pH 5 for peptoids with overall lower total monomers (Figures 5C and S5). Interestingly, we find that introduction of oleyl lipids often increased hemolysis at both pH 5 and 7. This suggests that oleyl-based peptoids may not be well tolerated due to background levels of hemolysis at physiological pH.

To investigate how the peptoid lipid block impacts in vivo expression, Nutshells were administered IV to Balb/c mice and the resulting luminescence in liver, lung and spleen were quantified after 6 h (Figure 5D–F). Even without changing the cationic headgroup, luciferase expression spans 4 orders of magnitude, demonstrating the impact of altering the lipid block in this series. Changing lipid block also impacts the liver selectivity of luciferase expression, but to a lesser extent than headgroup modifications (Figure 5E). The highest in vivo expression was observed for peptoids at intermediate values for both the number of lipid monomers (∼6) and total number of lipid carbons (∼55). This could imply that the hydrophilicity of each monomer due to the peptoid backbone must be offset by increasing total carbon number for delivery-related processes such as membrane fusion and endosomal escape to occur. Peptoids **236**, **245**, **263**, and **264** emerged as the top performers from Fluc expression screening. Interestingly, these peptoids are all 6 monomers in length and contain the 2-ethylhexyl (Ehx) branched monomer. In vivo Fluc expression of all candidates was fit to a multiparameter model containing the 4 input parameters to understand the individual contributions of each factor to overall performance (Figure S6). This model suggests that expression has a very strong second-order dependence on both the number of total lipid carbons and the number of unsaturated or branched monomers, a strong dependence on if the peptoids contained Ole or Ehx as the unsaturated/branched monomer, and a modest dependence on the total number of lipid monomers.

Based on this model, the maximal predicted expression was achieved using peptoids containing 5–6 total lipid monomers, 55 total lipid carbons, and 2–3 Ehx monomers as the nonlinear lipid. Notably, 3 out of the 4 top performing peptoids (**236**, **263**, and **264**) possessed attributes that closely aligned with this prediction. The fourth top candidate (**245**) had 3 out of 4 optimal attributes (lipid monomer number, nonlinear monomers, and Ehx as the branched/unsaturated monomer), with the total number of lipid carbons just outside of the predicted range (Table 1 and Figure 5D). One limitation of the DOE used for peptoid generation is that it neglected the monomer sequence as a variable, so we generated a peptoid with matched composition to peptoid **236**, but with a different monomer order (**266**, Figure S7). Physical characteristics, such as size, encapsulation, and hemolysis, were all similar between these two isomers (Table S6). In vivo luciferase expression was found to be within error for the two sequence variations, suggesting that monomer composition, rather than order, drives major differences between peptoids (Figure S7). This further validates the parametrization and screening criteria employed and future peptoid variations could look at sequence as secondary factor only, which will be the subject of further exploration. The top 4 candidates for in vivo luciferase expression were advanced into additional experiments to evaluate production of secreted proteins.

**Table 1:** Lipid Properties of Top-Performing Peptoids Compared to DOE Model-Predicted Optimal Configuration and Preoptimization Peptoid from the Screening Library^a^

peptoid	lipid monomers	total lipid carbons	branched/unsaturated monomers	branched/unsaturated identity	luciferase expression (p/s)
**226** (preoptimization)	4	48	0	none	2.46 ± 0.55 × 10^7^
**236**	6	56	4	Ehx	1.08 ± 0.41 × 10^9^
**245**	6	44	4	Ehx	2.93 ± 0.46 × 10^9^
**263**	6	52	2	Ehx	1.06 ± 0.27 × 10^9^
**264**	6	56	2	Ehx	1.90 ± 0.46 × 10^9^
**model predicted**	6.20 ± 1	52.5 ± 8.8	3.77 ± 0.8	Ehx	

a Data = mean ± SD (*n* = 3).

**Figure 6:**
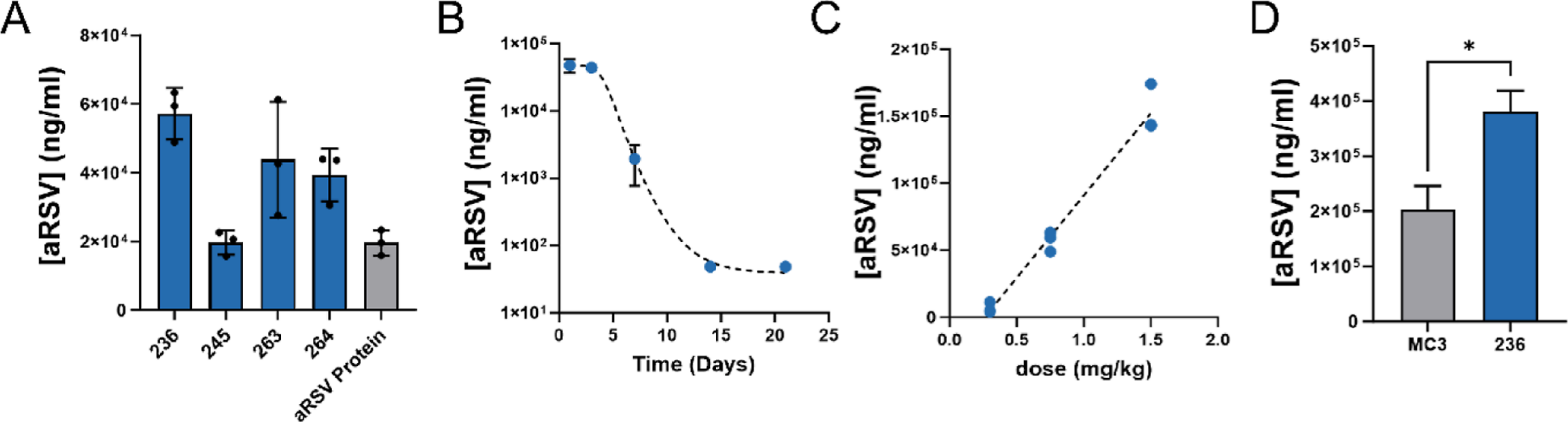
aRSV expression in Balb/c mice from the optimized peptoid nanoparticles. (A) Comparison of aRSV protein levels in serum 24 h after 0.75 mg/kg dose of peptoid-formulated mRNA. aRSV protein control group dosed at 3 mg/kg. (B) Time course of aRSV in serum after 0.75 mg/kg dose of Nutshell **236**. (C) Escalating level of aRSV in Balb/c serum after 24 h following increasing dose of mRNA formulated in peptoid **236**. (D) Comparison of aRSV serum level 24 h post 1.5 mg/kg IV administration of peptoid **236** compared to MC3.

To understand how Nutshells performed for the in vivo production of biologics, the top 4 peptoid candidates from the optimized DOE model were evaluated for their ability to produce an anti-RSV antibody in Balb/c mice. Severe viral infections of the respiratory tract such as human RSV account for a large number of hospitalizations and mortalities worldwide.^62^ To date, recombinant antibody therapies such as Motavizumab are available to treat RSV infection, but no mRNA-based therapies have been approved.^63^ We chose anti-RSV as a model biologic to demonstrate complex protein production with this platform due to the availability of the Motavizumab sequence and published benchmarking data.^63^ aRSV-containing Nutshells were formulated from 2 separate mRNA molecules encoding the heavy and light chain at a 2:1 mass ratio. All particles were below 120 nm in size with mRNA encapsulation greater than 85% (Table S7). Peptoid particles were administered IV at 0.75 mg/kg mRNA dose in Balb/c mice, and serum levels of aRSV were compared after 24 h by an antihuman IgG ELISA. Particles containing peptoids **263** and **236** resulted in the highest levels of secreted protein expression (Figure 6A). While there is not a perfect correlation between Fluc and secreted protein expression, the two correlate strongly (Figure S8). Peptoid **266**, the lipid monomer sequence variation of peptoid **236**, was also tested and expression of aRSV found to be within error (Figure S9). Expression time course and dose-escalation studies were carried out on Nutshell **236** which showed the highest overall aRSV titers (Figure 6B). With this candidate, aRSV titers were observed in the serum for the past 5 days, and serum titers showed a linear dependence on injected dose at 0.3, 0.75, and 1.5 mg/kg (Figure 6C). Serum expression from the 1.5 mg/kg IV dose was compared to a benchmark formulation, DLin-MC3-DMA (MC3), which is therapeutically used in the liver-targeting siRNA therapeutic Onpattro.^45^ In this comparison, Nutshell **236** showed an approximately 2-fold increase in anti-RSV serum levels above MC3 (Figure 6D). Resulting anti-RSV serum levels from the same Nutshell **236** dose are several folds above the therapeutic IC_50_ reported for Motavizumab against RSV strain A2.^64^ Together, this validates the use of this peptoid platform for the mRNA delivery and production of therapeutically relevant levels of secreted antibodies.

**Figure 7:**
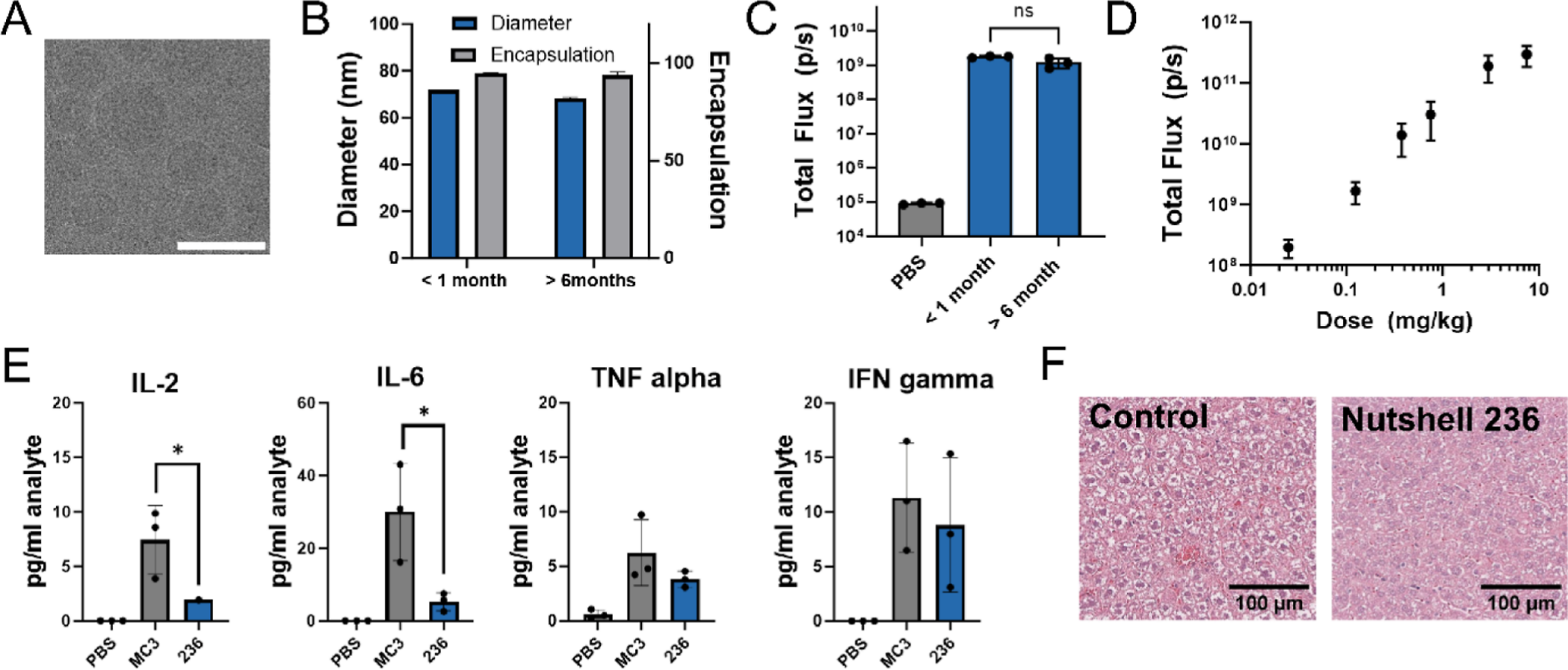
Characterization and tolerability of lead Nutshell **236**. (A) Cryo-TEM images of Nutshell 236. Scale bar = 100 nm. (B) Physical stability of Nutshell **236** after >6 months frozen storage. (C) In vivo expression after >6 months frozen storage. (D) Increasing Fluc expression in Balb/c mice 6 h after escalating dose of Nutshell **236** from 0.2 mg/kg up to 10 mg/kg. (E) Serum proinflammatory cytokines levels 6 h post treatment with 0.75 mg/kg dose of Nutshell **236** containing Fluc mRNA compared to benchmark MC3 formulation. PBS control was below LOD in all conditions shown. * *p* < 0.05 (unpaired *t* test). Data = mean ± SD (*n* = 3). (F) H&E staining of murine liver tissue after repeated (q14d, 2 times) 0.75 mg/kg dose of Nutshell **236** compared to PBS-treated liver (20× magnification). Tissue sections were collected 6 h after the second dose.

Successful clinical development of LNP therapies requires both stability and tolerability.^65^ Stability of Nutshell **236** during storage was monitored after −80 °C for over 6 months with no growth in particle size or change in encapsulation observed (Figure 7B). After 6 months of frozen storage, no change in Fluc expression in balb/c mice was observed (Figure 7C). To further validate expression levels of Nutshell **236**, we compared Fluc expression at matched doses to SM-102 and MC3 and showed serval fold higher expression than MC3 and comparable performance to SM-102 (Figure S10). Additionally, capillary electrophoresis was used to confirm RNA integrity after frozen storage (Figure S11). We also characterized particle structure with cryogenic-TEM and which showed that a majority of particles have a dense core morphology without bleb features observed (Figure 7A). Together, frozen stability and the spherical dense particle morphology support the practical use of Nutshells for therapeutic use. Fluorescence imaging was used to confirm the majority of particles show RNA loading, in line with other similar reports (Figure S12).^43,66^ Nanoparticle tracking analysis was used to understand the performance and stability of the particles in the presence of serum. We see similar behavior between Nutshell **236** and benchmark LNP SM-102 (Figure S13).^67^ Interestingly, a poor-performing peptoid for IV delivery that localizes in the lung rather than the liver showed much more significant changes in particle size distribution upon serum exposure (Figure S13, Nutshell **98**). One limitation of some LNP therapeutic candidates is the tolerability and reactogenicity of the ionizable lipid portion.^68−71^ Murine administration of Nutshell **236** at escalating IV doses from 0.3 to 7.5 mg/kg resulted in no observable signs of tolerability concerns, and Fluc expression continued to increase across this wide dose range (Figure 7D) with >97% expression in the liver (Figure S14). We further confirmed that the distribution of Fluc expression used throughout this study matched the distribution of peptoid molecules by using LC-MS to quantify the total amount of peptoid material in the primary organs, which showed very similar results (Figure S14). Histopathology and multiplexed cytokine analysis were collected at the 0.75 mg/kg dose in mice to further examine tolerability. Qualitative hematoxylin and eosin (H&E) staining on liver samples showed no signs of tissue damage compared to a phosphate-buffered saline (PBS)-treated control (Figure 7F). Proinflammatory cytokine markers, IL-2, IL-6, TNF-alpha, and INF-gamma, showed negligible elevation compared to both benchmark MC3 formulation and PBS control (Figure 7E). Induced levels of IL-6, which is known to be associated with innate immune recognition of LNPs, are not elevated for Nutshell **236** compared to MC3, supporting tolerability in mice.^70^ Additional cytokine levels and time points can be found in the Supporting Information (Figures S15 and 16). ALT, AST, and ALP levels also show limited change 24 h post dosing Nutshell **236** in line with the literature values^21^ (Figure S17). Together, these data suggest that Nutshell **236** does not illicit strong reactogenic responses in mice.

## Conclusions

Here, we show that peptoid-based LNPs provide a highly tunable platform for optimizing mRNA delivery for both intracellular and secreted protein expression. By clustering a large peptoid library, we surveyed a wide range of different peptoid types and side chain functionalities and screened representative structural candidates for reporter gene expression in mice. Structural clusters showed clear differences in organ selectivity and total expression, which allowed us to demonstrate connections between peptide structure and in vivo expression among major organs. As one example of tuning peptoid properties to match a therapeutic focus, we downselected structural themes with liver selectivity and high expression that served as the foundation for further headgroup and lipid block optimization. We found that changing the charged group changes the expression localization of Nutshells among the major organs including lung, spleen, and liver. Additional optimization of the lipid portion of peptoids containing this Apd cationic group was accomplished by a parametrization methodology whereby we used DOE to systematically identify ideal combinations of number of lipid monomers, total number of lipid carbons, number of branched or unsaturated monomers, and whether 2-ethylhexyl or oleyl monomers were used.

Throughout the course of this multistep screening and optimization process, several key relationships between peptoid structure and activity were observed. First, we identified that peptoids with low-basicity amines such as diethanolamine (Dea) and 2-amino-1,3-propanediol (Apd) had better selectivity for expression in the liver. This contrasts with traditional LNPs which require a much more basic tertiary amine and may be attributable to the hydrophilic environment provided by the peptoid backbone which modulates particle p*K*_a_. The optimal liver-expressing cluster contained peptoids with a single charged monomer and an intermediate number of lipid monomers. We found that spleen expression was highest with peptoids that had the largest overall molecular weights and highest numbers of total and lipid monomers, and lung expression was maximized with highly cationic peptoids. During lipid optimization, we showed that a balance between the number of lipid monomers and length of carbon chain had to be achieved, with the optimal performance coming from 6 lipid monomers and 55 carbons. Finally, the branched 2-ethylhexyl (Ehx) significantly outperformed the oleyl (Ole) monomer as a nonstraight chain lipid. By combining these ideal properties, peptoid **236** was identified as an ideal candidate to demonstrate proof-of-concept in vivo expression of a therapeutic protein.

Optimized Nutshell **236** showed liver selectivity above 97% and expressed sustained titers of aRSV in mouse serum validating the platform for systemic delivery. The optimized lead showed limited reactogenicity in mice and was storage stable for more than 6 months. While further studies in higher species are ongoing to explore tolerability and clinical translation, these data support peptoid-based Nutshell particles as a demonstrative platform for mRNA therapeutic delivery for a wide range of therapeutic targets. The nonviral delivery field is continually in search of innovative materials as complexing agents, particularly those that bring discovery into expanded regions of chemical space. We demonstrate that the distinct structural properties of peptoids lend them to favorable mRNA delivery performance relative to existing benchmarks, tunable biodistribution patterns based on their physicochemical properties, process and storage stability during manufacturing, and positive early indications of tolerability and translation of therapeutically relevant mRNA cargos.

## Experimental Methods

### Synthesis of Peptoids

Peptoids were synthesized by the submonomer method reported by Zuckermann et al.^27^ on a Protein Technologies Chorus solid-phase synthesizer. In a representative synthesis, polystyrene-supported MBHA Fmoc-protected Rink amide resin (200 mg scale, 0.64 mmol/g loading, Protein Technologies) was used as a solid support. For the bromoacetylation step, resin was combined with a 1:1 mixture of 0.8 M bromoacetic acid and 0.8 M *N*,*N*′-diisopropylcarbodiimide (DIC) for 15 min. Amine displacement was carried out using a 1 M solution of amine monomer in DMF for 45 min. Each addition was followed by washing with DMF to remove unreacted materials. Bromoacetylation and displacement steps were repeated for each monomer addition, building the target peptoid from the C-terminus to N-terminus.

Following synthesis, crude peptoids were cleaved from resin using 5 mL of a mixture of 95:5 trifluoroacetic acid (TFA): water for 40 min at room temperature. Resin was removed by filtration, and the filtrate was diluted with water to form a milky white suspension, followed by addition of Diaion HP-20SS beads for (heterogeneous) solid phase extraction of peptoids. The suspension was mixed well and incubated for 20 min at room temperature. Solid phase resin was collected by filtration by using a fritted disposable syringe. Next, the solid phase extracted peptoids were released by addition of ethanol, and the filtrate was concentrated using a vacuum centrifuge. The crude peptoids were further purified by reverse-phase flash chromatography (Biotage Selekt) using a C4 column and a gradient from 60 to 95% ACN/H_2_O containing 0.1 vol % TFA. Purity and identity were assayed with LC-MS system consisting of a TOF mass spectrometer (Agilent Infinity, 6230 LC/TOF). Chromatographic separation was performed using a Waters Acquity UPLC Peptide BEH C8 column (2.1 × 100 mm) at 40 °C. The gradient system was used at a flow rate of 0.2 mL/min: initially, the mobile phase consisted of acetonitrile–water-formic acid (60:40:0.1; vol/vol/vol) containing 10 mM ammonium formate; then it was programmed in a linear manner to isopropanol-acetonitrile-formic acid (80:20:0.1) containing 10 mM ammonium formate over 11 min.

### RNA Bench IVT and Characterization

mRNA was transcribed from DNA template encoding firefly luciferase using HiScribe T7 high yield RNA synthesis kit (New England Biolabs, Ipswich, MA, USA). *N*^1^-Methylpseudo-UTP (m1ΨTP) (TriLink, San Diego, CA, USA) was used in replaced of UTP for the reaction and CleanCap (TriLink, San Diego, CA, USA) was used for cotranscriptional capping during IVT. The reaction was performed for 4 h at 37 °C.

DNA template was removed through TURBO DNase digestion (ThermoFisher Scientific, Waltham, MA, USA) for 30 min at 37 °C. The reaction mix was purified in a two-step process, which involves purification through a cellulose packed column followed by RNAClean XP beads (Beckman Coulter) clean up. The purified mRNA was quantified spectrophotometrically using nanodrop (ThermoFisher Scientific, Waltham, MA, USA) and analyzed using a Labchip GX Touch nucleic acid analyzer (PerkinElmer, Waltham, MA, USA)

### Molecular Properties Predictions

In silico peptoid property predictions were carried out by using the ChemOffice plugin for Microsoft Excel (PerkinElmer). Peptoids were input using SMILES strings, and the following functions used for specific predictions: ChemPropStd (Molecular Weight), Molecular Networks (Log *P*, p*K*_a_), Molecular Topology (Total Polar Surface Area). Charge at pH 5.5 was calculated using the sum of individual charge states for each determined p*K*_a_ at pH 5.5 using the Henderson–Hasselbalch equation.

### Hierarchical Clustering

Clustering was performed using the Hierarchical Clustering platform within the JMP Statistical Discovery Package (JMP, Version 17. SAS Institute Inc., Cary, NC, 1989–2023). Clustering was performed according to the Ward method, with data for the 7 input parameters (Molecular Weight, Log *P*, Polar Surface Area, Lipid Monomers, Lipid Carbons, Total Monomers, and Charge at pH 5.5) standardized by column. The number of clusters was manually set to 12. This number was selected because it provided a reasonable number of different groups to explicitly test via in vivo screening and efficiently subdivided the library into relatively even groups. When the cluster number was lower than 12, the size of the largest cluster dominated the majority of the candidates and thus did not adequately group the library by physical properties. When more clusters were introduced, many subdivisions with only 1 or 2 members were created.

### Multivariate Model and DOE

The effect of different lipid block parameters on in vivo expression and physical characteristics were designed using a response surface model generated by JMP. The model was created specifying total lipid monomers, nonlinear monomers, and total lipid carbons as continuous variables and nonlinear monomer type (oleyl or branched) as a categorical factor. The final set of 34 peptoids was selected by iteratively generating designs and ruling out parameter sets that were not achievable with the specified monomer pool. Multivariable model was fit based on all first and second order interactions between factors using the Standard Least Squares personality.

### Nutshell Formulation and Processing

Nutshells were formulated by rapid microfluidic mixing using Nutcracker Therapeutics’ proprietary high-throughput microfluidic system. Briefly, ethanol solutions containing peptoid, DSPC, Cholesterol, and DMG-PEG2000 at weight ratio of 20:1.79:7.16:1.84, were mixed in a microfluidic turbulent mixing structure with 10 mM sodium citrate buffer (pH 5.0) containing synthesized mRNA at a ratio of 1:3 by volume matching a 1:3 flow rate (combined flow rate of 2 mL/min). Peptoid based particles were subsequently dialyzed against 1–2 L of tris-sucrose buffer (TS7, pH 7.4) overnight at 4 °C using Pur-A-Lyzer mini 12,000 dialysis devices (Sigma-Aldrich, Burlington, MA).

### In Vivo Studies

All animal studies were conducted by Lumigenics LLC (Hercules, CA) under accreditation by the California State Department of Public Health and under the IACUC and Veterinarian Supervision. For imaging studies, female Balb/c mice (6–8 weeks old) were obtained from Charles River and acclimatized for a minimum of 3 days prior to experiments. The animals were maintained on a 12 h light cycle in a temperature and humidity controlled room. A daily health check was performed as well as a food and water check. For dosing, mice were injected with 0.125 mg/kg Fluc mRNA-containing Nutshells intravenously in a 100 μL total injection volume. At 6 h postinjection, mice were anesthetized with isoflurane, injected IP with 30 mg/mL D-luciferin (Gold Bio, cat. LUCK-1G) at a dose of 10 μL per gram bodyweight intraperitoneally. Imaging was done 10 min post D-luciferin injection using an IVIS Spectrum Imaging System (Caliper Life Sciences). Immediately after in vivo imaging, mice were terminally bled by cardiac puncture and euthanized. The organs of interest were harvested, placed in black 24-well plates, and imaged. Living Image software (PerkinElmer) was used to quantify the total photon flux in the regions of interest. For secreted protein studies, mice were injected with aRSV mRNA-containing nutshells intravenously in a 100 μL total injection volume. Mice were euthanized after 24 h, with serum collection at 6 and 24 h postinjection. Serum was used to quantify secreted aRSV levels using ELISA following the manufacturer’s recommendation for IgG Human Elisa Kit Thermo fisher (cat: BMS2091) using a standard curve generated from the aRSV protein.

### Electron Microscopy

Cryo-TEM was performed by Nanoimaging Services (San Diego, CA). For sample preparation, a 3 μL drop of approximately 1 × 10^14^ particles/mL was applied to a 2/1C-FlatCu-Mesh grid (Electron Microscopy Sciences) that had been plasma-cleaned for 10 s using a 25% O_2_/75% Ar mixture in a Solarus 950 Plasma Cleaner (Gatan). Grids were vitrified by plunging into liquid ethane using a Vitrobot Mark IV (Thermo Fisher Scientific): blot time 6s, 4 °C, 100% humidity. After vitrification, the grids were kept under liquid nitrogen and transferred to a Thermo Fisher Scientific Glacios Cryo Transmission Electron Microscope (Cryo-TEM) operated at 200 kV and equipped with a Falcon 3 direct electron detector. Images were acquired using Leginon software at magnifications of 73,000× (0.200 nm/pixel) and 28,000× (0.524 nm/pixel), a nominal underfocus of −5.5 to −3.5 μm, and electron doses of ∼10–25 e–/Å2. The images were analyzed and scaled using ImageJ.

## Supporting Information Available

Detailed description of peptoid sequences and characterization data, particle characterization data, and Supplemental Figures (PDF)

## References

[ref1] Baden L. R.; El Sahly H. M.; Essink B.; Kotloff K.; Frey S.; Novak R.; Diemert D.; Spector S. A.; Rouphael N.; Creech C. B.; McGettigan J.; Khetan S.; Segall N.; Solis J.; Brosz A.; Fierro C.; Schwartz H.; Neuzil K.; Corey L.; Gilbert P.; Janes H.; Follmann D.; Marovich M.; Mascola J.; Polakowski L.; Ledgerwood J.; Graham B. S.; Bennett H.; Pajon R.; Knightly C.; Leav B.; Deng W.; Zhou H.; Han S.; Ivarsson M.; Miller J.; Zaks T. Efficacy and Safety of the mRNA-1273 SARS-CoV-2 Vaccine. N. Engl. J. Med. 2021, 384 (5), 403–416. 10.1056/NEJMoa2035389. 33378609 PMC7787219

[ref2] Polack F. P.; Thomas S. J.; Kitchin N.; Absalon J.; Gurtman A.; Lockhart S.; Perez J. L.; Pérez Marc G.; Moreira E. D.; Zerbini C.; Bailey R.; Swanson K. A.; Roychoudhury S.; Koury K.; Li P.; Kalina W. V.; Cooper D.; Frenck R. W.; Hammitt L. L.; Türeci Ö.; Nell H.; Schaefer A.; Ünal S.; Tresnan D. B.; Mather S.; Dormitzer P. R.; Şahin U.; Jansen K. U.; Gruber W. C. Safety and Efficacy of the BNT162b2 mRNA Covid-19 Vaccine. N. Engl. J. Med. 2020, 383 (27), 2603–2615. 10.1056/NEJMoa2034577. 33301246 PMC7745181

[ref3] Barbier A. J.; Jiang A. Y.; Zhang P.; Wooster R.; Anderson D. G. The clinical progress of mRNA vaccines and immunotherapies. Nat. Biotechnol. 2022, 40, 840–854. 10.1038/s41587-022-01294-2. 35534554

[ref4] Kon E.; Ad-El N.; Hazan-Halevy I.; Stotsky-Oterin L.; Peer D. Targeting cancer with mRNA–lipid nanoparticles: key considerations and future prospects. Nat. Rev. Clin. Oncol. 2023, 20 (11), 739–754. 10.1038/s41571-023-00811-9. 37587254

[ref5] Gu J.; Xu Z.; Liu Q.; Tang S.; Zhang W.; Xie S.; Chen X.; Chen J.; Yong K.-T.; Yang C.; Xu G. Building a Better Silver Bullet: Current Status and Perspectives of Non-Viral Vectors for mRNA Vaccines. Adv. Healthcare Mater. 2023, 13 (3), 2302409 10.1002/adhm.202302409. 37964681

[ref6] Wang C.; Zhang Y.; Dong Y. Lipid Nanoparticle–mRNA Formulations for Therapeutic Applications. Acc. Chem. Res. 2021, 54, 4283–4293. 10.1021/acs.accounts.1c00550. 34793124 PMC10068911

[ref7] Hajj K. A.; Whitehead K. A. Tools for translation: non-viral materials for therapeutic mRNA delivery. Nat. Rev. Mater. 2017, 2 (10), 17056 10.1038/natrevmats.2017.56.

[ref8] Zhang Y.; Sun C.; Wang C.; Jankovic K. E.; Dong Y. Lipids and Lipid Derivatives for RNA Delivery. Chem. Rev. 2021, 121, 12181–12277. 10.1021/acs.chemrev.1c00244. 34279087 PMC10088400

[ref9] Bevers S.; Kooijmans S. A. A.; Van de Velde E.; Evers M. J. W.; Seghers S.; Gitz-Francois J. J. J. M.; van Kronenburg N. C. H.; Fens M. H. A. M.; Mastrobattista E.; Hassler L.; Sork H.; Lehto T.; Ahmed K. E.; El Andaloussi S.; Fiedler K.; Breckpot K.; Maes M.; Van Hoorick D.; Bastogne T.; Schiffelers R. M.; De Koker S. mRNA-LNP vaccines tuned for systemic immunization induce strong antitumor immunity by engaging splenic immune cells. Mol. Ther. 2022, 30 (9), 3078–3094. 10.1016/j.ymthe.2022.07.007. 35821637 PMC9273295

[ref10] Kowalski P. S.; Rudra A.; Miao L.; Anderson D. G. Delivering the Messenger: Advances in Technologies for Therapeutic mRNA Delivery. Mol. Ther. 2019, 27, 710–728. 10.1016/j.ymthe.2019.02.012. 30846391 PMC6453548

[ref11] Mrksich K.; Padilla M. S.; Joseph R. A.; Han E. L.; Kim D.; Palanki R.; Xu J.; Mitchell M. J. Influence of ionizable lipid tail length on lipid nanoparticle delivery of mRNA of varying length. J. Biomed. Mater. Res., Part A 2024, 112, 1494–1505. 10.1002/jbm.a.37705. PMC11239295 38487970

[ref12] Miao L.; Lin J.; Huang Y.; Li L.; Delcassian D.; Ge Y.; Shi Y.; Anderson D. G. Synergistic lipid compositions for albumin receptor mediated delivery of mRNA to the liver. Nat. Commun. 2020, 11 (1), 2424 10.1038/s41467-020-16248-y. 32415122 PMC7229004

[ref13] Li Z.; Amaya L.; Pi R.; Wang S. K.; Ranjan A.; Waymouth R. M.; Blish C. A.; Chang H. Y.; Wender P. A. Charge-altering releasable transporters enhance mRNA delivery in vitro and exhibit in vivo tropism. Nat. Commun. 2023, 14 (1), 6983 10.1038/s41467-023-42672-x. 37914693 PMC10620205

[ref14] Xue L.; Gong N.; Shepherd S. J.; Xiong X.; Liao X.; Han X.; Zhao G.; Song C.; Huang X.; Zhang H.; Padilla M. S.; Qin J.; Shi Y.; Alameh M.-G.; Pochan D. J.; Wang K.; Long F.; Weissman D.; Mitchell M. J. Rational Design of Bisphosphonate Lipid-like Materials for mRNA Delivery to the Bone Microenvironment. J. Am. Chem. Soc. 2022, 144, 9926–9937. 10.1021/jacs.2c02706. 35616998

[ref15] Liu J.; Chang J.; Jiang Y.; Meng X.; Sun T.; Mao L.; Xu Q.; Wang M. Fast and Efficient CRISPR/Cas9 Genome Editing In Vivo Enabled by Bioreducible Lipid and Messenger RNA Nanoparticles. Adv. Mater. 2019, 31 (33), 1902575 10.1002/adma.201902575. PMC6732788 31215123

[ref16] Goldman R. L.; Vittala Murthy N. T.; Northen T. P.; Balakrishnan A.; Chivukula S.; Danz H.; Tibbitts T.; Dias A.; Vargas J.; Cooper D.; Gopani H.; Beaulieu A.; Kalnin K. V.; Plitnik T.; Karmakar S.; Dasari R.; Landis R.; Karve S.; DeRosa F. Understanding structure activity relationships of Good HEPES lipids for lipid nanoparticle mRNA vaccine applications. Biomaterials 2023, 301, 122243 10.1016/j.biomaterials.2023.122243. 37480759

[ref17] Chen Z.; Tian Y.; Yang J.; Wu F.; Liu S.; Cao W.; Xu W.; Hu T.; Siegwart D. J.; Xiong H. Modular Design of Biodegradable Ionizable Lipids for Improved mRNA Delivery and Precise Cancer Metastasis Delineation In Vivo. J. Am. Chem. Soc. 2023, 145, 24302–24314. 10.1021/jacs.3c09143. 37853662

[ref18] Tanaka H.; Takahashi T.; Konishi M.; Takata N.; Gomi M.; Shirane D.; Miyama R.; Hagiwara S.; Yamasaki Y.; Sakurai Y.; Ueda K.; Higashi K.; Moribe K.; Shinsho E.; Nishida R.; Fukuzawa K.; Yonemochi E.; Okuwaki K.; Mochizuki Y.; Nakai Y.; Tange K.; Yoshioka H.; Tamagawa S.; Akita H. Self-Degradable Lipid-Like Materials Based on “Hydrolysis Accelerated by the Intra-Particle Enrichment of Reactant (HyPER)” for Messenger RNA Delivery. Adv. Funct. Mater. 2020, 30 (34), 1910575 10.1002/adfm.201910575.

[ref19] Han X.; Zhang H.; Butowska K.; Swingle K. L.; Alameh M.-G.; Weissman D.; Mitchell M. J. An Ionizable Lipid Toolbox for RNA Delivery. Nat. Commun. 2021, 12 (1), 7233 10.1038/s41467-021-27493-0. 34903741 PMC8668901

[ref20] Fenton O. S.; Kauffman K. J.; McClellan R. L.; Appel E. A.; Dorkin J. R.; Tibbitt M. W.; Heartlein M. W.; DeRosa F.; Langer R.; Anderson D. G. Bioinspired Alkenyl Amino Alcohol Ionizable Lipid Materials for Highly Potent In Vivo mRNA Delivery. Adv. Mater. 2016, 28 (15), 2939–2943. 10.1002/adma.201505822. 26889757 PMC5245883

[ref21] Lam K.; Leung A.; Martin A.; Wood M.; Schreiner P.; Palmer L.; Daly O.; Zhao W.; McClintock K.; Heyes J. Unsaturated, Trialkyl Ionizable Lipids Are Versatile Lipid-Nanoparticle Components for Therapeutic and Vaccine Applications. Adv. Mater. 2023, 35 (15), 2209624 10.1002/adma.202209624. 36680477

[ref22] Miao L.; Li L.; Huang Y.; Delcassian D.; Chahal J.; Han J.; Shi Y.; Sadtler K.; Gao W.; Lin J.; Doloff J. C.; Langer R.; Anderson D. G. Delivery of mRNA vaccines with heterocyclic lipids increases anti-tumor efficacy by STING-mediated immune cell activation. Nat. Biotechnol. 2019, 37 (10), 1174–1185. 10.1038/s41587-019-0247-3. 31570898

[ref23] Lokugamage M. P.; Vanover D.; Beyersdorf J.; Hatit M. Z. C.; Rotolo L.; Echeverri E. S.; Peck H. E.; Ni H.; Yoon J.-K.; Kim Y.; Santangelo P. J.; Dahlman J. E. Optimization of lipid nanoparticles for the delivery of nebulized therapeutic mRNA to the lungs. Nat. Biomed. Eng. 2021, 5 (9), 1059–1068. 10.1038/s41551-021-00786-x. 34616046 PMC10197923

[ref24] Jiang A. Y.; Witten J.; Raji I. O.; Eweje F.; MacIsaac C.; Meng S.; Oladimeji F. A.; Hu Y.; Manan R. S.; Langer R.; Anderson D. G. Combinatorial development of nebulized mRNA delivery formulations for the lungs. Nat. Nanotechnol. 2023, 19, 364–375. 10.1038/s41565-023-01548-3. 37985700 PMC10954414

[ref25] Kline M. A.; Guo L.; Zuckermann R. N. Sequence-Controlled Peptoid Polymers: Bridging the Gap between Biology and Synthetic Polymers. In Sequence-Controlled Polymers; John Wiley & Sons, Ltd, 2018; pp 183–227. 10.1002/9783527806096.ch7.

[ref26] Zuckermann R. N.; Kerr J. M.; Kent S. B. H.; Moos W. H. Efficient Method for the Preparation of Peptoids [Oligo(N-Substituted Glycines)] by Submonomer Solid-Phase Synthesis. J. Am. Chem. Soc. 1992, 114 (26), 10646–10647. 10.1021/ja00052a076.

[ref27] Connolly M. D.; Xuan S.; Molchanova N.; Zuckermann R. N. Submonomer synthesis of sequence defined peptoids with diverse side-chains. In Methods in Enzymology; Elsevier, 2021; Vol. 656, pp 241–270. 10.1016/bs.mie.2021.04.022. 34325788

[ref28] Clapperton A. M.; Babi J.; Tran H. A Field Guide to Optimizing Peptoid Synthesis. ACS Polym. Au 2022, 2 (6), 417–429. 10.1021/acspolymersau.2c00036. 36536890 PMC9756346

[ref29] Barry M. E.; Davidson E. C.; Zhang C.; Patterson A. L.; Yu B.; Leonardi A. K.; Duzen N.; Malaviya K.; Clarke J. L.; Finlay J. A.; Clare A. S.; Chen Z.; Ober C. K.; Segalman R. A. The Role of Hydrogen Bonding in Peptoid-Based Marine Antifouling Coatings. Macromolecules 2019, 52 (3), 1287–1295. 10.1021/acs.macromol.8b02390.

[ref30] Greco I.; Emborg A. P.; Jana B.; Molchanova N.; Oddo A.; Damborg P.; Guardabassi L.; Hansen P. R. Characterization, Mechanism of Action and Optimization of Activity of a Novel Peptide-Peptoid Hybrid against Bacterial Pathogens Involved in Canine Skin Infections. Sci. Rep. 2019, 9 (1), 3679 10.1038/s41598-019-39042-3. 30842436 PMC6403271

[ref31] Nielsen J. E.; Alford M. A.; Yung D. B. Y.; Molchanova N.; Fortkort J. A.; Lin J. S.; Diamond G.; Hancock R. E. W.; Jenssen H.; Pletzer D.; Lund R.; Barron A. E. Self-Assembly of Antimicrobial Peptoids Impacts Their Biological Effects on ESKAPE Bacterial Pathogens. ACS Infect. Dis. 2022, 8 (3), 533–545. 10.1021/acsinfecdis.1c00536. 35175731 PMC8962514

[ref32] Wender P. A.; Mitchell D. J.; Pattabiraman K.; Pelkey E. T.; Steinman L.; Rothbard J. B. The Design, Synthesis, and Evaluation of Molecules That Enable or Enhance Cellular Uptake: Peptoid Molecular Transporters. Proc. Natl. Acad. Sci. U.S.A. 2000, 97 (24), 13003–13008. 10.1073/pnas.97.24.13003. 11087855 PMC27168

[ref33] Huang M. L.; Ehre D.; Jiang Q.; Hu C.; Kirshenbaum K.; Ward M. D. Biomimetic Peptoid Oligomers as Dual-Action Antifreeze Agents. Proc. Natl. Acad. Sci. U.S.A. 2012, 109 (49), 19922–19927. 10.1073/pnas.1212826109. 23169638 PMC3523862

[ref34] Zhang M.; Qiu Z.; Yang K.; Zhou W.; Liu W.; Lu J.; Guo L. Design, Synthesis and Antifreeze Properties of Biomimetic Peptoid Oligomers. Chem. Commun. 2023, 59 (46), 7028–7031. 10.1039/D3CC01062G. 37128894

[ref35] Huang C.-Y.; Uno T.; Murphy J. E.; Lee S.; Hamer J. D.; Escobedo J. A.; Cohen F. E.; Radhakrishnan R.; Dwarki V.; Zuckermann R. N. Lipitoids—Novel Cationic Lipids for Cellular Delivery of Plasmid DNA in Vitro. Chem. Biol. 1998, 5 (6), 345–354. 10.1016/S1074-5521(98)90173-9. 9653553

[ref36] Nogueira S. S.; Schlegel A.; Maxeiner K.; Weber B.; Barz M.; Schroer M. A.; Blanchet C. E.; Svergun D. I.; Ramishetti S.; Peer D.; Langguth P.; Sahin U.; Haas H. Polysarcosine-Functionalized Lipid Nanoparticles for Therapeutic mRNA Delivery. ACS Appl. Nano Mater. 2020, 3 (11), 10634–10645. 10.1021/acsanm.0c01834.

[ref37] Wilhelmy C.; Keil I. S.; Uebbing L.; Schroer M. A.; Franke D.; Nawroth T.; Barz M.; Sahin U.; Haas H.; Diken M.; Langguth P. Polysarcosine-Functionalized mRNA Lipid Nanoparticles Tailored for Immunotherapy. Pharmaceutics 2023, 15 (8), 2068 10.3390/pharmaceutics15082068. 37631282 PMC10458461

[ref38] Kang D. D.; Hou X.; Wang L.; Xue Y.; Li H.; Zhong Y.; Wang S.; Deng B.; McComb D. W.; Dong Y. Engineering LNPs with polysarcosine lipids for mRNA delivery. Bioact. Mater. 2024, 37, 86–93. 10.1016/j.bioactmat.2024.03.017. 38523704 PMC10957522

[ref39] He D.; Wagner E. Defined Polymeric Materials for Gene Delivery. Macromol. Biosci. 2015, 15 (5), 600–612. 10.1002/mabi.201400524. 25655078

[ref40] Reinhard S.; Wagner E. How to Tackle the Challenge of siRNA Delivery with Sequence-Defined Oligoamino Amides. Macromol. Biosci. 2017, 17 (1), 1600152 10.1002/mabi.201600152. 27328447

[ref41] Lin Y.; Luo X.; Burghardt T.; Dorrer S.; Höhn M.; Wagner E.; Lächelt U. Chemical Evolution of Amphiphilic Xenopeptides for Potentiated Cas9 Ribonucleoprotein Delivery. J. Am. Chem. Soc. 2023, 145 (28), 15171–15179. 10.1021/jacs.3c01902. 37395536 PMC10360056

[ref42] Haase F.; Pöhmerer J.; Yazdi M.; Grau M.; Zeyn Y.; Wilk U.; Burghardt T.; Höhn M.; Hieber C.; Bros M.; Wagner E.; Berger S. Lipoamino bundle LNPs for efficient mRNA transfection of dendritic cells and macrophages show high spleen selectivity. Eur. J. Pharm. Biopharm. 2024, 194, 95–109. 10.1016/j.ejpb.2023.11.025. 38065313

[ref43] Sabnis S.; Kumarasinghe E. S.; Salerno T.; Mihai C.; Ketova T.; Senn J. J.; Lynn A.; Bulychev A.; McFadyen I.; Chan J.; Almarsson O. ¨.; Stanton M. G.; Benenato K. E. A Novel Amino Lipid Series for mRNA Delivery: Improved Endosomal Escape and Sustained Pharmacology and Safety in Non-human Primates. Mol. Ther. 2018, 26 (6), 1509–1519. 10.1016/j.ymthe.2018.03.010. 29653760 PMC5986714

[ref44] Lee S. M.; Cheng Q.; Yu X.; Liu S.; Johnson L. T.; Siegwart D. J. A Systematic Study of Unsaturation in Lipid Nanoparticles Leads to Improved mRNA Transfection In Vivo. Angew. Chem., Int. Ed. 2021, 60 (11), 5848–5853. 10.1002/anie.202013927. PMC8100975 33305471

[ref45] Jayaraman M.; Ansell S. M.; Mui B. L.; Tam Y. K.; Chen J.; Du X.; Butler D.; Eltepu L.; Matsuda S.; Narayanannair J. K.; Rajeev K. G.; Hafez I. M.; Akinc A.; Maier M. A.; Tracy M. A.; Cullis P. R.; Madden T. D.; Manoharan M.; Hope M. J. Maximizing the Potency of siRNA Lipid Nanoparticles for Hepatic Gene Silencing In Vivo**. Angew. Chem., Int. Ed. 2012, 51 (34), 8529–8533. 10.1002/anie.201203263. PMC3470698 22782619

[ref46] Omo-Lamai S.; Zamora M. E.; Patel M. N.; Wu J.; Nong J.; Wang Z.; Peshkova A.; Majumder A.; Melamed J. R.; Chase L. S.; Essien E.; Weissman D.; Muzykantov V. R.; Marcos-Contreras O. A.; Myerson J. W.; Brenner J. S. Physicochemical Targeting of Lipid Nanoparticles to the Lungs Induces Clotting: Mechanisms and Solutions. Adv. Mater. 2024, 36, 2312026 10.1002/adma.202312026. PMC11209818 38394670

[ref47] Kim M.; Jeong M.; Lee G.; Lee Y.; Park J.; Jung H.; Im S.; Yang J.; Kim K.; Lee H. Novel piperazine-based ionizable lipid nanoparticles allow the repeated dose of mRNA to fibrotic lungs with improved potency and safety. Bioeng. Transl. Med. 2023, 8 (6), e10556 10.1002/btm2.10556. 38023699 PMC10658549

[ref48] Rybakova Y.; Kowalski P. S.; Huang Y.; Gonzalez J. T.; Heartlein M. W.; DeRosa F.; Delcassian D.; Anderson D. G. mRNA Delivery for Therapeutic Anti-HER2 Antibody Expression In Vivo. Mol. Ther. 2019, 27 (8), 1415–1423. 10.1016/j.ymthe.2019.05.012. 31160223 PMC6698250

[ref49] Ramaswamy S.; Tonnu N.; Tachikawa K.; Limphong P.; Vega J. B.; Karmali P. P.; Chivukula P.; Verma I. M. Systemic Delivery of Factor IX Messenger RNA for Protein Replacement Therapy. Proc. Natl. Acad. Sci. U.S.A. 2017, 114 (10), E1941–E1950. 10.1073/pnas.1619653114. 28202722 PMC5347596

[ref50] Patel P.; Ibrahim N. M.; Cheng K. The Importance of Apparent pKa in the Development of Nanoparticles Encapsulating siRNA and mRNA. Trends Pharmacol. Sci. 2021, 42, 448–460. 10.1016/j.tips.2021.03.002. 33875229 PMC8148296

[ref51] Carrasco M. J.; Alishetty S.; Alameh M.-G.; Said H.; Wright L.; Paige M.; Soliman O.; Weissman D.; Cleveland T. E.; Grishaev A.; Buschmann M. D. Ionization and structural properties of mRNA lipid nanoparticles influence expression in intramuscular and intravascular administration. Commun. Biol. 2021, 4 (1), 956 10.1038/s42003-021-02441-2. 34381159 PMC8358000

[ref52] Whitehead K. A.; Dorkin J. R.; Vegas A. J.; Chang P. H.; Veiseh O.; Matthews J.; Fenton O. S.; Zhang Y.; Olejnik K. T.; Yesilyurt V.; Chen D.; Barros S.; Klebanov B.; Novobrantseva T.; Langer R.; Anderson D. G. Degradable lipid nanoparticles with predictable in vivo siRNA delivery activity. Nat. Commun. 2014, 5, 4277 10.1038/ncomms5277. 24969323 PMC4111939

[ref53] Cheng Q.; Wei T.; Farbiak L.; Johnson L. T.; Dilliard S. A.; Siegwart D. J. Selective organ targeting (SORT) nanoparticles for tissue-specific mRNA delivery and CRISPR–Cas gene editing. Nat. Nanotechnol. 2020, 15, 313–320. 10.1038/s41565-020-0669-6. 32251383 PMC7735425

[ref54] Dilliard S. A.; Cheng Q.; Siegwart D. J. On the mechanism of tissue-specific mRNA delivery by selective organ targeting nanoparticles. Proc. Natl. Acad. Sci. U.S.A. 2021, 118 (52), e2109256118 10.1073/pnas.2109256118. 34933999 PMC8719871

[ref55] Rajappan K.; Tanis S. P.; Mukthavaram R.; Roberts S.; Nguyen M.; Tachikawa K.; Sagi A.; Sablad M.; Limphong P.; Leu A.; Yu H.; Chivukula P.; Payne J. E.; Karmali P. Property-Driven Design and Development of Lipids for Efficient Delivery of siRNA. J. Med. Chem. 2020, 63 (21), 12992–13012. 10.1021/acs.jmedchem.0c01407. 33119286

[ref56] Hashiba K.; Sato Y.; Taguchi M.; Sakamoto S.; Otsu A.; Maeda Y.; Shishido T.; Murakawa M.; Okazaki A.; Harashima H. Branching Ionizable Lipids Can Enhance the Stability, Fusogenicity, and Functional Delivery of mRNA. Small Sci. 2023, 3 (1), 2200071 10.1002/smsc.202200071. PMC11935957 40212983

[ref57] Hajj K. A.; Ball R. L.; Deluty S. B.; Singh S. R.; Strelkova D.; Knapp C. M.; Whitehead K. A. Branched-Tail Lipid Nanoparticles Potently Deliver mRNA In Vivo due to Enhanced Ionization at Endosomal pH. Small 2019, 15 (6), 1805097 10.1002/smll.201805097. 30637934

[ref58] Hajj K. A.; Melamed J. R.; Chaudhary N.; Lamson N. G.; Ball R. L.; Yerneni S. S.; Whitehead K. A. A Potent Branched-Tail Lipid Nanoparticle Enables Multiplexed mRNA Delivery and Gene Editing In Vivo. Nano Lett. 2020, 20, 5167–5175. 10.1021/acs.nanolett.0c00596. 32496069 PMC7781386

[ref59] Hammel M.; Fan Y.; Sarode A.; Byrnes A. E.; Zang N.; Kou P.; Nagapudi K.; Leung D.; Hoogenraad C. C.; Chen T.; Yen C.-W.; Hura G. L. Correlating the Structure and Gene Silencing Activity of Oligonucleotide-Loaded Lipid Nanoparticles Using Small-Angle X-Ray Scattering. ACS Nano 2023, 17 (12), 11454–11465. 10.1021/acsnano.3c01186. 37279108 PMC10311593

[ref60] Sarode A.; Fan Y.; Byrnes A. E.; Hammel M.; Hura G. L.; Fu Y.; Kou P.; Hu C.; Hinz F. I.; Roberts J.; Koenig S. G.; Nagapudi K.; Hoogenraad C. C.; Chen T.; Leung D.; Yen C.-W. Predictive High-Throughput Screening of PEGylated Lipids in Oligonucleotide-Loaded Lipid Nanoparticles for Neuronal Gene Silencing. Nanoscale Adv. 2022, 4 (9), 2107–2123. 10.1039/D1NA00712B. 36133441 PMC9417559

[ref61] Alabi C. A.; Love K. T.; Sahay G.; Yin H.; Luly K. M.; Langer R.; Anderson D. G. Multiparametric approach for the evaluation of lipid nanoparticles for siRNA delivery. Proc. Natl. Acad. Sci. U.S.A. 2013, 110 (32), 12881–12886. 10.1073/pnas.1306529110. 23882076 PMC3740846

[ref62] Borchers A. T.; Chang C.; Gershwin M. E.; Gershwin L. J. Respiratory Syncytial Virus-a Comprehensive Review. Clin. Rev. Allergy Immunol. 2013, 45 (3), 331–379. 10.1007/s12016-013-8368-9. 23575961 PMC7090643

[ref63] Jacobino S. R.; Nederend M.; Reijneveld J. F.; Augustijn D.; Jansen J. H. M.; Meeldijk J.; Reiding K. R.; Wuhrer M.; Coenjaerts F. E. J.; Hack C. E.; Bont L. J.; Leusen J. H. W. Reformatting Palivizumab and Motavizumab from IgG to Human IgA Impairs Their Efficacy against RSV Infection in Vitro and in Vivo. mAbs 2018, 10 (3), 453–462. 10.1080/19420862.2018.1433974. 29553863 PMC5939987

[ref64] Wu H.; Pfarr D. S.; Johnson S.; Brewah Y. A.; Woods R. M.; Patel N. K.; White W. I.; Young J. F.; Kiener P. A. Development of Motavizumab, an Ultra-Potent Antibody for the Prevention of Respiratory Syncytial Virus Infection in the Upper and Lower Respiratory Tract. J. Mol. Biol. 2007, 368 (3), 652–665. 10.1016/j.jmb.2007.02.024. 17362988

[ref65] Schoenmaker L.; Witzigmann D.; Kulkarni J. A.; Verbeke R.; Kersten G.; Jiskoot W.; Crommelin D. J. A. mRNA-lipid nanoparticle COVID-19 vaccines: Structure and stability. Int. J. Pharm. 2021, 601, 120586 10.1016/j.ijpharm.2021.120586. 33839230 PMC8032477

[ref66] Kulkarni J. A.; Darjuan M. M.; Mercer J. E.; Chen S.; van der Meel R.; Thewalt J. L.; Tam Y. Y. C.; Cullis P. R. On the Formation and Morphology of Lipid Nanoparticles Containing Ionizable Cationic Lipids and siRNA. ACS Nano 2018, 12 (5), 4787–4795. 10.1021/acsnano.8b01516. 29614232

[ref67] Berger M.; Degey M.; Leblond Chain J.; Maquoi E.; Evrard B.; Lechanteur A.; Piel G. Effect of PEG Anchor and Serum on Lipid Nanoparticles: Development of a Nanoparticles Tracking Method. Pharmaceutics 2023, 15 (2), 597 10.3390/pharmaceutics15020597. 36839919 PMC9962341

[ref68] Parhiz H.; Brenner J. S.; Patel P. N.; Papp T. E.; Shahnawaz H.; Li Q.; Shi R.; Zamora M. E.; Yadegari A.; Marcos-Contreras O. A.; Natesan A.; Pardi N.; Shuvaev V. V.; Kiseleva R.; Myerson J. W.; Uhler T.; Riley R. S.; Han X.; Mitchell M. J.; Lam K.; Heyes J.; Weissman D.; Muzykantov V. R. Added to pre-existing inflammation, mRNA-lipid nanoparticles induce inflammation exacerbation (IE). J. Controlled Release 2022, 344, 50–61. 10.1016/j.jconrel.2021.12.027. PMC8695324 34953981

[ref69] Hassett K. J.; Higgins J.; Woods A.; Levy B.; Xia Y.; Hsiao C. J.; Acosta E.; Almarsson O. ¨.; Moore M. J.; Brito L. A. Impact of lipid nanoparticle size on mRNA vaccine immunogenicity. J. Controlled Release 2021, 335, 237–246. 10.1016/j.jconrel.2021.05.021. 34019945

[ref70] Korzun T.; Moses A. S.; Diba P.; Sattler A. L.; Taratula O. R.; Sahay G.; Taratula O.; Marks D. L. From Bench to Bedside: Implications of Lipid Nanoparticle Carrier Reactogenicity for Advancing Nucleic Acid Therapeutics. Pharmaceuticals 2023, 16 (8), 1088 10.3390/ph16081088. 37631003 PMC10459564

[ref71] Tahtinen S.; Tong A.-J.; Himmels P.; Oh J.; Paler-Martinez A.; Kim L.; Wichner S.; Oei Y.; McCarron M. J.; Freund E. C.; Amir Z. A.; De La Cruz C. C.; Haley B.; Blanchette C.; Schartner J. M.; Ye W.; Yadav M.; Sahin U.; Delamarre L.; Mellman I. IL-1 and IL-1ra Are Key Regulators of the Inflammatory Response to RNA Vaccines. Nat. Immunol. 2022, 23 (4), 532–542. 10.1038/s41590-022-01160-y. 35332327

