# Supplementary material for: Discovery of a Peptoid-Based Nanoparticle Platform for Therapeutic mRNA Delivery via Diverse Library Clustering and Structural Parametrization
Source: ACS Nano. 2024 Aug 6;18(33):22181–93. doi: 10.1021/acsnano.4c05513 (PMC11342374; doi:10.1021/acsnano.4c05513)
Supplement: Supplementary file 1 — nn4c05513_si_001.pdf [file nn4c05513_si_001.pdf]

Supporting Information

Discovery of a peptoid-based nanoparticle platform for therapeutic mRNA delivery via diverse library clustering and structural parameterization

Elizabeth R. Webster[1], Nicole E. Peck[1], Juan Diego Echeverri[1], Shima Gholizadeh[1], Wei-Lun Tang[1], Rinette Woo[1], Anushtha Sharma[1], Weiqun Liu[1], Chris S. Rae[1], Adrienne Sallets[1], Gowrisudha Adusumilli[1], Kannan Gunasekaran[1], Ole A. W. Haabeth[1], Meredith Leong[1], Ronald N. Zuckermann[2], Samuel Deutsch[1], Colin J. McKinlay[1]

[1]Nutcracker Therapeutics, 5980 Horton Street Suite 350, Emeryville, CA 94608, USA

[2]Molecular Foundry, Lawrence Berkeley National Laboratory, Berkeley, CA 94720, USA

1. Supplementary Figures and Tables
2. Supplemental Methods
3. Synthetic Characterization Data

I. Supplementary Figures and Tables:

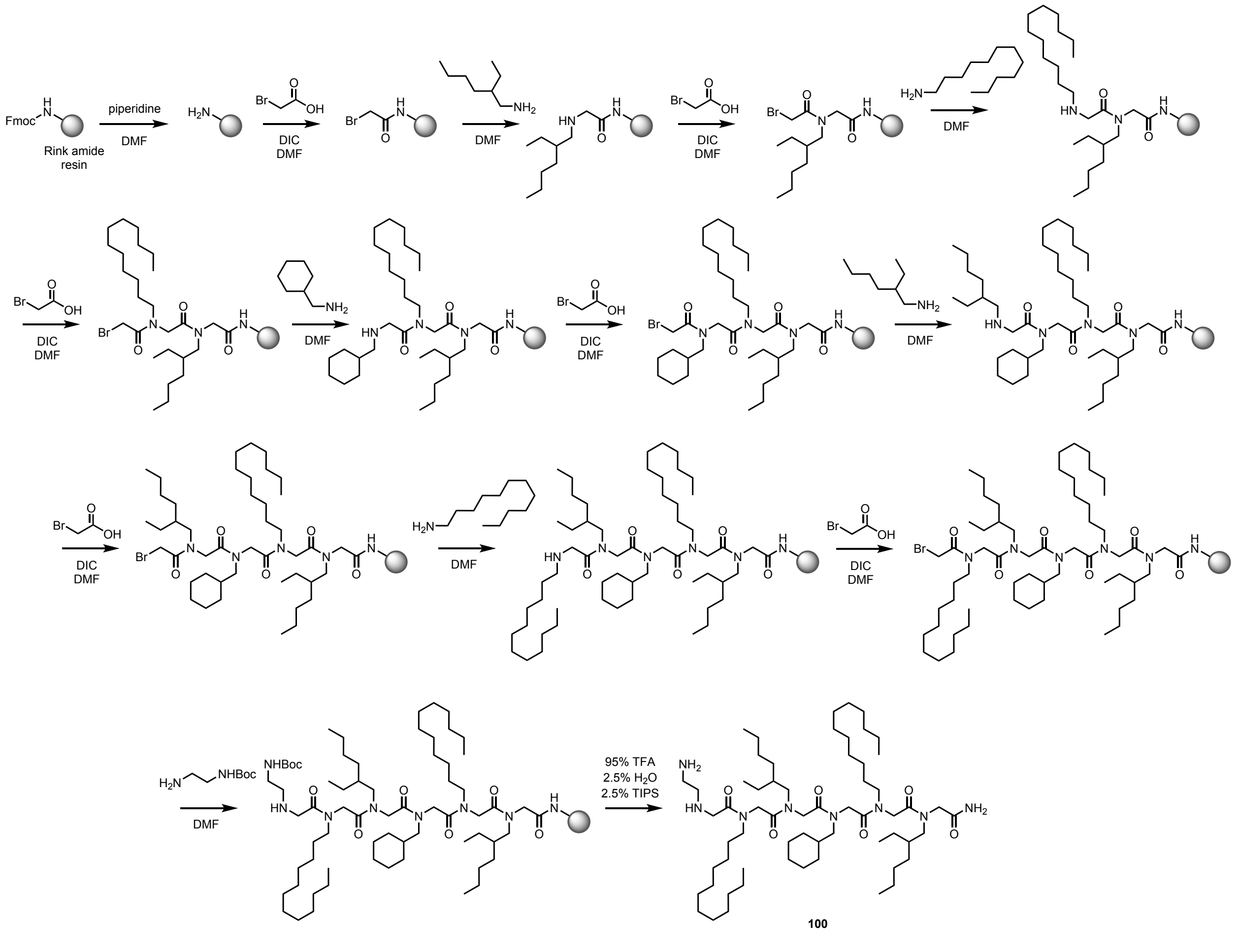

Figure S1. Full scheme for the synthesis of a representative peptoid lipid **100** utilizing the sub-monomer approach.

Table S1. Table of monomer sequences, predicted physical properties, and cluster assignments for peptoid library.

Peptoid	Monomer Sequence	Molecular Weight (Da)	LogP	Polar Surface Area	Lipid Monomers	Total Lipid Carbons	Total Monomers	Total Charge pH 5.5	Cluster
1	Aet-Pet-Pet-Aet-Pet-Pet-Aet-Pet-Pet-Oct-Oct	1623.154	6.5	336.3	8	64	11	3.0	1
2	Aet-Pet-Pet-Aet-Pet-Pet-Aet-Pet-Pet-Dec-Dec	1679.262	8.2	336.3	8	68	11	3.0	1
3	Aet-Pet-Pet-Aet-Pet-Pet-Aet-Pet-Pet-Dod-Dod	1735.37	10.0	336.3	8	72	11	3.0	1
4	Aet-Mpe-Mpe-Aet-Mpe-Mpe-Aet-Mpe-Mpe-Dod-Dod	1915.526	9.4	391.7	8	78	11	3.0	1
5	Aet-Pet-Pet-Aet-Pet-Pet-Aet-Pet-Pet-Ode-Ode	1903.694	15.4	336.3	8	84	11	3.0	1
6	Aet-Pet-Pet-Aet-Pet-Pet-Aet-Pet-Pet-Dec	1481.94	5.8	316.0	7	58	10	3.0	1
7	Aet-Pet-Pet-Aet-Pet-Pet-Aet-Pet-Pet-Dec-Dec-Dec	1876.584	11.3	356.6	9	78	12	3.0	1
8	Aet-Pet-Pet-Aet-Pet-Pet-Aet-Pet-Pet-Dec-Dec-Dec-Dec	2073.906	14.6	376.9	10	88	13	3.0	3
9	Aet-Pet-Pet-Dec-Dec	834.204	6.4	162.4	4	36	5	1.0	5
10	Aet-Pet-Pet-Aet-Pet-Pet-Dec-Dec	1256.733	7.3	249.3	6	52	8	2.0	2
11	Aet-Pet-Pet-Aet-Pet-Pet-Aet-Pet-Pet-Aet-Pet-Pet-Dec-Dec	2101.791	9.1	423.2	10	84	14	4.0	3
12	Aet-Pet-Pet-Aet-Pet-Pet-Aet-Pet-Pet-Aet-Pet-Pet-Aet-Pet-Pet-Dec-Dec	2524.32	10.0	510.2	12	100	17	5.0	4
13	Aet-Pet-Aet-Pet-Aet-Pet-Dec-Dec	1195.65	4.7	275.4	5	44	8	3.0	2
14	Aet-Aet-Aet-Dec-Dec	712.038	1.2	214.4	2	20	5	3.0	7
15	Aet-Aet-Aet-Aet-Aet-Dec-Dec	912.28	-1.7	307.1	2	20	7	5.0	7
16	Aet-Pet-Pet-Dec-Aet-Pet-Pet-Dec-Aet-Pet-Pet	1679.262	8.2	336.3	8	68	11	3.0	1
17	Dec-Aet-Pet-Pet-Aet-Pet-Pet-Aet-Pet-Pet-Dec	1679.262	8.2	336.3	8	68	11	4.0	1
18	Aet-Pet-Pet-Aet-Pet-Pet-Aet-Pet-Pet-Dec-Dec-Dec-Dec-Dec	2271.228	17.7	397.2	11	98	14	3.0	3
19	Aet-Pet-Pet-Aet-Pet-Pet-Dec-Dec-Dec-Dec-Dec	1848.699	16.8	310.3	9	82	11	2.0	1
20	Aet-Pet-Pet-Aet-Pet-Pet-Aet-Pet-Pet-Aet-Pet-Pet-Dec-Dec-Dec-Dec-Dec	2693.757	18.6	484.2	13	114	17	4.0	4
21	Aet-Pet-Pet-Aet-Pet-Pet-Aet-Pet-Pet-Dec-Dec-Dec-Dec-Dec-Dec	2468.55	21.0	417.5	12	108	15	3.0	4
22	Aet-Pet-Pet-Aet-Pet-Pet-Aet-Pet-Pet-Dec-Dec-Dec-Dec-Dec-Dec-Dec	2665.872	24.1	437.8	13	118	16	3.0	4
23	Aet-Pet-Pet-Aet-Pet-Pet-Aet-Pet-Pet-Dec-Ehx-Dec-Ehx	2017.798	12.6	376.9	10	84	13	3.0	3
24	Mae-Pet-Pet-Mae-Pet-Pet-Mae-Pet-Pet-Dec-Dec-Dec-Dec	2115.987	15.8	334.9	10	88	13	3.0	3
25	Mae-Pet-Pet-Mae-Pet-Pet-Mae-Pet-Pet-Mae-Pet-Pet-Dec-Dec-Dec-Dec	2552.543	17.0	407.9	12	104	16	4.0	4
26	Mae-Pet-Pet-Mae-Pet-Pet-Mae-Pet-Pet-Dec-Dec	1721.343	9.4	294.3	8	68	11	3.0	1
27	Apr-Pet-Pet-Apr-Pet-Pet-Apr-Pet-Pet-Dec-Dec-Dec-Dec	2115.987	15.6	376.9	10	88	13	3.5	3
28	Apr-Pet-Pet-Apr-Pet-Pet-Apr-Pet-Pet-Apr-Pet-Pet-Dec-Dec-Dec-Dec	2552.543	16.9	463.9	12	104	16	4.5	4

29	Apr-Pet-Pet-Apr-Pet-Pet-Apr-Pet-Pet-Dec-Dec	1721.343	9.3	336.3	8	68	11	3.5	1
30	Mpi-Pet-Pet-Mpi-Pet-Pet-Mpi-Pet-Pet-Dec-Dec-Dec-Dec	2236.182	17.9	334.9	10	88	13	3.7	3
31	Mpi-Pet-Pet-Mpi-Pet-Pet-Mpi-Pet-Pet-Mpi-Pet-Pet-Dec-Dec-Dec-Dec	2712.803	19.9	407.9	12	104	16	4.7	4
32	Mpi-Pet-Pet-Mpi-Pet-Pet-Mpi-Pet-Pet-Dec-Dec	1841.538	11.5	294.3	8	68	11	3.7	1
33	Mpl-Pet-Pet-Mpl-Pet-Pet-Mpl-Pet-Pet-Dec-Dec-Dec-Dec	2194.101	17.3	334.9	10	88	13	3.0	3
34	Mpl-Pet-Pet-Mpl-Pet-Pet-Mpl-Pet-Pet-Mpl-Pet-Pet-Dec-Dec-Dec-Dec	2656.695	19.1	407.9	12	104	16	4.0	4
35	Mpl-Pet-Pet-Mpl-Pet-Pet-Mpl-Pet-Pet-Dec-Dec	1799.457	11.0	294.3	8	68	11	3.0	1
36	Aet-Dec-Dec-Dec-Dec	906.44	10.5	162.4	4	40	5	1.0	5
37	Apr-Dec-Dec-Dec-Dec	920.467	10.8	162.4	4	40	5	1.5	5
38	Nhp-Dec-Dec-Dec-Dec	921.451	10.9	156.6	4	40	5	2.0	5
39	Spm-Dec-Dec-Dec-Dec	977.563	10.8	174.4	4	40	5	2.5	8
40	Bpp-Dec-Dec-Dec-Dec	1046.67	10.7	168.9	4	40	5	2.9	8
41	Tae-Dec-Dec-Dec-Dec	1034.659	10.6	154.9	4	40	5	2.8	8
42	Imp-Dec-Dec-Dec-Dec	971.515	11.7	152.0	4	40	5	0.9	5
43	Aet-Aet-Pet-Pet-Dec-Dec-Dec-Dec	1328.969	11.4	249.3	6	56	8	2.0	2
44	Apr-Aet-Pet-Pet-Dec-Dec-Dec-Dec	1342.996	11.7	249.3	6	56	8	2.3	2
45	Nhp-Aet-Pet-Pet-Dec-Dec-Dec-Dec	1343.98	11.8	243.5	6	56	8	2.9	2
46	Spm-Aet-Pet-Pet-Dec-Dec-Dec-Dec	1400.092	11.7	261.4	6	56	8	3.3	2
47	Bpp-Aet-Pet-Pet-Dec-Dec-Dec-Dec	1469.199	11.6	255.8	6	56	8	3.9	2
48	Tae-Aet-Pet-Pet-Dec-Dec-Dec-Dec	1457.188	11.5	241.8	6	56	8	3.6	2
49	Imp-Aet-Pet-Pet-Dec-Dec-Dec-Dec	1394.044	12.6	238.9	6	56	8	1.8	2
50	Aet-Aet-Pet-Pet-Aet-Pet-Pet-Dec-Dec-Dec-Dec	1751.498	12.3	336.3	8	72	11	3.0	1
51	Apr-Aet-Pet-Pet-Aet-Pet-Pet-Dec-Dec-Dec-Dec	1765.525	12.6	336.3	8	72	11	3.3	1
52	Nhp-Aet-Pet-Pet-Aet-Pet-Pet-Dec-Dec-Dec-Dec	1766.509	12.6	330.5	8	72	11	3.9	1
53	Spm-Aet-Pet-Pet-Aet-Pet-Pet-Dec-Dec-Dec-Dec	1822.621	12.6	348.3	8	72	11	4.3	1
54	Bpp-Aet-Pet-Pet-Aet-Pet-Pet-Dec-Dec-Dec-Dec	1891.728	12.5	342.8	8	72	11	4.9	1
55	Tae-Aet-Pet-Pet-Aet-Pet-Pet-Dec-Dec-Dec-Dec	1879.717	12.4	328.8	8	72	11	4.6	1
56	Imp-Aet-Pet-Pet-Aet-Pet-Pet-Dec-Dec-Dec-Dec	1816.573	13.5	325.9	8	72	11	2.8	1
57	Aet-Aet-Pet-Pet-Aet-Pet-Pet-Dec-Ehx-Dec-Ehx	1695.39	10.2	336.3	8	68	11	3.0	1
58	Apr-Aet-Pet-Pet-Aet-Pet-Pet-Dec-Ehx-Dec-Ehx	1709.417	10.5	336.3	8	68	11	3.3	1
59	Nhp-Aet-Pet-Pet-Aet-Pet-Pet-Dec-Ehx-Dec-Ehx	1710.401	10.6	330.5	8	68	11	3.9	1
60	Spm-Aet-Pet-Pet-Aet-Pet-Pet-Dec-Ehx-Dec-Ehx	1766.513	10.5	348.3	8	68	11	4.3	1
61	Bpp-Aet-Pet-Pet-Aet-Pet-Pet-Dec-Ehx-Dec-Ehx	1835.62	10.4	342.8	8	68	11	4.9	1
62	Tae-Aet-Pet-Pet-Aet-Pet-Pet-Dec-Ehx-Dec-Ehx	1823.609	10.3	328.8	8	68	11	4.6	1

63	Imp-Aet-Pet-Pet-Aet-Pet-Pet-Dec-Ehx-Dec-Ehx	1760.465	11.4	325.9	8	68	11	2.8	1
64	Aet-Dod-Dod-Dod-Dod	1018.656	14.0	162.4	4	48	5	1.0	9
65	Aet-Dod-Dod-Dod-Dod-Dod	1244.032	18.0	182.7	5	60	6	1.0	9
66	Aet-Dod-Dod-Dod-Dod-Dod-Dod	1469.408	22.2	203.0	6	72	7	1.0	10
67	Aet-Aet-Dod-Dod-Dod-Dod-Dod-Dod	1569.529	20.7	249.3	6	72	8	2.0	10
68	Aet-Ode-Ode-Ode-Ode	1355.304	24.7	162.4	4	72	5	1.0	10
69	Aet-Ole-Ole-Ole-Ole	1347.24	23.0	162.4	4	72	5	1.0	10
70	Aet-Dod-Ehx-Dod-Ehx	906.44	10.2	162.4	4	40	5	1.0	5
71	Aet-Dod-Ole-Dod-Ole	1182.948	18.5	162.4	4	60	5	1.0	9
72	Frm-Dod-Dod-Aet-Dod-Dod	1046.666	13.8	170.7	4	48	6	1.0	9
73	Frm-Dod-Dod-Aet-Aet-Dod-Dod	1146.787	12.4	217.0	4	48	7	2.0	9
74	Frm-Dod-Dod-Apr-Apr-Dod-Dod	1174.841	13.0	217.0	4	48	7	2.0	9
75	Frm-Dod-Aet-Dod-Dod-Aet-Dod	1146.787	12.4	217.0	4	48	7	2.0	9
76	Eed-Dod-Dod-Dod-Dod	1074.764	15.5	139.6	4	48	5	1.0	9
77	Eed-Ole-Ole-Ole-Ole	1403.348	24.5	139.6	4	72	5	1.0	10
78	Frm-Dod-Apr-Dod-Dod-Apr-Dod-Dod-Apr-Dod	1739.741	20.1	303.9	6	72	10	3.0	10
79	Frm-Dod-Apr-Ole-Dod-Apr-Ole-Dod-Apr-Ole	1986.179	26.8	303.9	6	90	10	3.0	10
80	Dec-Dec-Dec-Dec	806.319	11.9	116.1	4	40	4	1.0	5
81	Frm-Dec-Dec-Dec-Dec	834.329	11.7	124.3	4	40	5	0.0	5
82	Aet-Ehx-Ehx-Ehx-Ehx	794.224	6.4	162.4	4	32	5	1.0	5
83	Aet-Ehx-Ehx-Ehx-Ehx-Ehx	963.492	8.5	182.7	5	40	6	1.0	5
84	Aet-Ole-Ehx-Ole-Ehx	1070.732	14.7	162.4	4	52	5	1.0	9
85	Aet-Dec-Iso-Dec-Iso	850.332	8.6	162.4	4	36	5	1.0	5
86	Frm-Dec-Ehx-Dec-Ehx-Aet-Aet	978.463	6.7	217.0	4	36	7	2.0	5
87	Frm-Dec-Ehx-Dec-Ehx-Die-Die	1146.787	11.6	171.4	4	36	7	2.0	5
88	Die-Dec-Ehx-Dec-Ehx	934.494	10.8	139.6	4	36	5	1.0	5
89	Apr-Dec-Ehx-Dec-Ehx	864.359	8.8	162.4	4	36	5	1.5	5
90	Nme-Aet-Dec-Ehx-Dec-Ehx	965.464	7.5	191.9	4	36	6	1.8	5
91	Aet-Met-Dec-Ehx-Dec-Ehx	921.411	7.7	182.7	4	36	6	1.0	5
92	Aet-Met-Met-Dec-Ehx-Dec-Ehx	992.49	7.0	203.0	4	36	7	1.0	5
93	Aet-Nme-Dec-Ehx-Dec-Ehx	965.464	7.5	191.9	4	36	6	1.0	5
94	Aet-Nme-Nme-Dec-Ehx-Dec-Ehx	1080.596	6.6	221.5	4	36	7	1.0	5
95	Aet-CprDec-Ehx-Dec-Ehx	979.447	7.3	220.0	4	26	5	1.1	5
96	Aet-Cpr-Cpr-Dec-Ehx-Dec-Ehx	1108.562	6.1	277.6	4	36	7	1.2	5
97	Aet-Dod-Dod-Dod-Ehx	962.548	12.0	162.4	4	44	5	1.0	5
98	Apr-Dod-Ehx-Dod-Ehx	920.467	10.5	162.4	4	40	5	1.5	5
99	Aet-Cym-Dod-Ehx-Dod-Ehx	1059.665	11.4	182.7	5	47	6	1.0	5
100	Aet-Dod-Ehx-Cym-Dod-Ehx	1059.665	11.4	182.7	5	47	6	1.0	5
101	Pip-Dod-Ehx-Dod-Ehx-Dod	988.586	12.0	139.6	5	52	6	1.6	5
102	Mop-Dod-Ehx-Dod-Ehx	990.558	10.8	148.8	4	40	5	1.5	5
103	Aet-Dod-Dec-Dod-Dec	962.548	12.3	162.4	4	44	5	1.0	5

104	Aet-Dod-Iso-Dod-Iso	906.44	10.4	162.4	4	40	5	1.0	5
105	Frm-Dec-Ehx-Dec-Ehx	835.273	8.8	153.4	4	36	5	0.0	5
106	Frm-Nme-Dec-Nme-Dec-Nme-Dec-Nme-Dec	1294.857	8.2	242.5	4	40	9	0.0	5
107	Frm-Dec-Smb-Smb-Dec-Smb-Smb-Dec-Smb-Smb	1604.231	17.6	225.9	9	78	10	0.0	11
108	Frm-Nme-Smb-Smb-Nme-Smb-Smb-Nme-Smb-Smb	1357.661	5.5	253.6	6	48	10	0.0	5
109	Frm-Hex-Hex-Hex-Hex-Hex-Hex-Hex-Hex	1286.969	13.8	205.6	8	48	9	0.0	11
110	Nme-Smb-Smb-Cpr-Smb-Smb-Apr-Smb-Smb-Cpr-Smb-Smb	1794.173	7.2	388.4	8	64	12	2.0	1
111	Nme-Smb-Smb-Apr-Smb-Smb-Nme-Cpr-Apr-Smb-Smb-Cpr	1701.045	2.1	423.6	6	48	12	3.1	1
112	Nme-Smb-Smb-Cpr-Smb-Dec-Apr-Smb-Dec-Cpr-Smb-Dec	1902.527	12.0	388.4	8	70	12	2.0	1
113	Apr-Smb-Smb-Apr-Smb-Smb-Apr-Smb-Smb	1326.699	5.0	295.7	6	48	9	3.5	2
114	Frm-Dec-Cye-Dec-Cye	831.241	8.3	153.4	4	36	5	0.0	5
115	Frm-Dec-Pet-Dec-Pet	819.145	6.8	153.4	4	36	5	0.0	5
116	Apr-Smb-Smb-Dec-Smb-Smb-Dec-Smb-Smb	1493.047	13.7	243.6	8	68	9	1.5	11
117	Nme-Pet-Pet-Apr-Pet-Pet-Nme-Cpr-Apr-Pet-Pet-Cpr	1701.045	-0.1	423.6	6	48	12	3.1	1
118	Apr-Mme-Mme-Dec-Mme-Mme-Dec-Mme-Mme	1284.778	2.9	289.8	2	20	9	1.5	5
119	Nme-Mme-Mme-Apr-Mme-Mme-Nme-Cpr-Apr-Mme-Mme-Cpr	1492.776	-8.7	469.8	0	0	12	3.1	7
120	Nme-Smb-Smb-Apr-Smb-Smb-Cpr-Smb-Smb-Apr-Smb-Smb	1779.206	7.2	377.1	8	64	12	3.0	1
121	Nme-Smb-Smb-Cpr-Smb-Smb-Cpr-Smb-Smb-Cpr-Smb-Smb	1809.14	7.1	399.7	8	64	12	1.1	1
122	Nme-Smb-Smb-Apr-Smb-Smb-Nme-Cpr-Apr-Smb-Smb-Cpr-Smb-Smb-Apr-Smb-Smb-Cpr	2589.124	6.0	608.8	10	80	18	4.1	4
123	Nhp-Dod-Dod-Dod-Dod	1033.667	14.4	156.6	4	48	5	2.0	9
124	Nmp-Dec-Dec-Dec-Dec	935.478	11.4	145.6	4	40	5	1.0	5
125	Mor-Dec-Dec-Dec-Dec	933.462	11.1	136.8	4	40	5	0.2	5
126	Nhe-Dec-Dec-Dec-Dec	907.424	10.5	156.6	4	40	5	1.9	5
127	Nhp-Pet-Dec-Dec-Dec-Dec	1082.655	12.0	176.9	5	48	6	2.0	5
128	Frm-Dod-Dod-Apr-Dod-Dod	1060.693	14.2	170.7	4	48	6	1.0	9
129	Frm-Dod-Dod-Pet-Apr-Pet-Dod-Dod	1383.101	16.5	211.3	6	64	8	1.0	11
130	Frm-Ehx-Dod-Aet-Dod-Ehx	948.477	10.3	170.7	4	40	6	1.0	5
131	Nhp-Dec-Dec-Dec	724.129	7.6	136.3	3	30	4	2.0	6
132	Eea-Dec-Ehx-Dec	710.102	7.0	127.5	4	28	4	1.9	6
133	Nhp-Nmp-Dec-Dec-Dec	993.558	11.5	151.8	3	30	5	2.0	5
134	Nhp-Dec-Ehx-Dec	696.075	6.6	136.3	3	28	4	2.0	6
135	Nhe-Dec-Ehx-Dec-Ehx	851.316	8.5	156.6	4	36	5	1.9	5
136	Nhe-Dec-Ole-Dec-Ole	1127.824	16.8	156.6	4	56	5	1.9	9
137	Aet-Dec-Ehx-Dec	681.064	6.3	142.1	3	28	4	1.0	6
138	Apr-Dec-Ehx-Dec	695.091	6.6	142.1	3	28	4	1.5	6

139	Frm-Dod-Dod-Mae-Dod-Dod	1060.693	14.2	156.7	4	48	6	1.0	9
140	Frm-Dec-Dec-Aet-Dec-Dec	934.45	10.3	170.7	4	40	6	1.0	5
141	Frm-Dod-Pet-Dod-Aet-Dod-Pet-Dod	1369.074	16.2	211.3	6	64	8	1.0	11
142	Frm-Dod-Nme-Dod-Aet-Dod-Nme-Dod	1262.947	12.6	212.7	4	48	8	2.0	9
143	Eea-Dec-Dec-Dec-Dec	935.478	11.2	147.8	5	40	5	1.9	5
144	Nhe-Dod-Dod-Dod-Dod	1019.64	14.1	156.6	4	48	5	1.9	9
145	Dea-Dec-Dec-Dec-Dec	951.477	10.1	168.0	4	40	5	2.5	8
146	Frm-Aet-Dec-Dec-Dec-Dec	934.45	10.3	170.7	4	40	6	1.0	5
147	Frm-Mae-Dec-Dec-Dec-Dec	948.477	10.6	156.7	4	40	6	1.0	5
148	Frm-Apr-Dec-Dec-Dec-Dec	948.477	10.6	170.7	4	40	6	1.0	5
149	Frm-Dod-Dod-Eed-Dod-Dod	1116.801	15.6	147.9	4	48	6	1.0	9
150	Frm-Dod-Dod-Mpi-Dod-Dod	1100.758	14.9	156.7	4	48	6	1.0	9
151	Frm-Dod-Pet-Dod-Mpi-Dod-Pet-Dod	1423.166	17.3	197.3	6	64	8	1.0	11
152	Frm-Dod-Fur-Dod-Aet-Dod-Fur-Dod	1320.942	13.2	229.7	6	58	8	1.0	11
153	Frm-Dod-Pet-Dod-Apr-Dod-Pet-Dod	1383.101	16.5	211.3	6	64	8	1.0	11
154	Frm-Dod-Pet-Ole-Aet-Ole-Pet-Dod	1533.366	20.6	211.3	6	76	8	1.0	10
155	Frm-Ole-Ole-Ole-Ole	1275.129	24.2	124.3	4	48	5	1.0	10
156	Eea-Dod-Ole-Dod-Ole	1047.694	14.8	147.8	5	60	5		9
157	Dea-Dod-Ehx-Dod-Ehx	893.397	9.5	147.8	4	40	5	1.9	5
158	Eea-Dod-Ehx-Dod-Ehx	879.37	9.1	147.8	5	40	5	1.9	5
159	Frm-Dod-Dod-Pet-Aet-Pet-Dod-Dod	1369.074	16.2	211.3	6	64	8	1.9	11
160	Frm-Pet-Dod-Dod-Aet-Dod-Dod-Pet	1369.074	16.2	211.3	6	64	8	1.0	11
161	Frm-Ehx-Pet-Dod-Aet-Dod-Pet-Ehx	1256.858	12.3	211.3	6	56	8	1.0	11
162	Frm-Dod-Pet-Ehx-Aet-Ehx-Pet-Dod	1227.816	13.9	185.3	6	56	8	1.0	11
163	Apr-Ole-Ehx-Ole-Ehx	1098.786	15.3	162.4	4	52	5	1.0	9
164	Nhe-Ole-Ehx-Ole-Ehx	1085.743	15.1	156.6	4	52	5	1.5	9
165	Apr-Ehx-Ole-Ehx-Ole	1084.759	15.0	162.4	4	52	5	1.9	9
166	Aet-Ole-Ole-Ole-Ole	1347.24	23.0	162.4	4	72	5		10
167	Frm-Dec-Ehx	411.631	4.2	83.7	2	18	3	0.0	6
168	Frm-Dec-Ehx-Dec-Ehx-Dec-Ehx-Dec-Ehx	1511.401	20.4	205.6	8	72	9	0.0	11
169	Frm-Dec-Ehx-Dec-Ehx-Dec-Ehx-Dec-Ehx-Dec-Ehx-Dec-Ehx	2244.581	31.1	286.8	12	108	13	0.0	12
170	Aet-Ole-Ehx-Ole-Ehx-Ole	1378.254	20.9	182.7	5	70	6	1.0	10
171	Aet-Pet-Ole-Ehx-Ole-Ehx	1231.936	15.8	182.7	5	60	6	1.0	9
172	Frm-Ole-Ehx-Aet-Ole-Ehx	1084.715	14.1	170.7	4	52	6	1.0	9
173	Frm-Dod-Dod-Aet-Aet-Dod-Dod	1160.814	12.3	217.0	4	48	7	2.1	9
174	Pip-Ole-Ehx-Ole-Ehx	1166.905	16.8	139.6	4	52	5	1.6	9
175	Mpi-Ole-Ehx-Ole-Ehx	1138.851	16.1	148.4	4	52	5	1.7	9
176	Mpn-Ole-Ehx-Ole-Ehx	1124.824	15.8	148.4	4	52	5	1.3	9
177	Apd-Ole-Ehx-Ole-Ehx	1101.742	14.1	176.8	4	52	5	2.6	8
178	Imp-Ole-Ehx-Ole-Ehx	1135.807	15.9	152.0	4	52	5	0.9	9

179	Mip-Ole-Ehx-Ole-Ehx	1149.834	16.1	152.0	4	52	5	0.9	9
180	Mop-Ole-Ehx-Ole-Ehx	1154.85	15.3	148.8	4	52	5	1.5	9
181	Mpp-Ole-Ehx-Ole-Ehx	1167.893	15.3	142.8	4	52	5	2.0	9
182	Aet-Ole-Ehx	593.942	6.1	121.8	2	26	3	1.0	6
183	Aet-Ole-Ehx-Ole-Ehx-Ole-Ehx	1547.522	23.0	203.0	6	78	7	1.0	10
184	Aet-Ole-Ehx-Ole-Ehx-Ole-Ehx-Ole	1855.044	29.5	223.3	7	96	8	1.0	12
185	Aet-Ole-Ehx-Ole-Ehx-Ole-Ehx-Ole-Ehx	2024.312	31.6	243.6	8	104	9	1.0	12
186	Aet-Ole-Ole-Ehx-Ehx	1070.732	14.7	162.4	4	52	5	1.0	9
187	Aet-Ehx-Ehx-Ole-Ole	1070.732	14.7	162.4	4	52	5	1.0	9
188	Aet-Cme-Ole-Ehx-Ole-Ehx	1199.847	13.5	220.0	4	52	6	1.1	9
189	Aet-Cme-Cme-Ole-Ehx-Ole-Ehx	1328.962	12.4	277.6	4	52	7	1.2	9
190	Aet-Ole-Ehx-Cme-Ole-Ehx	1242.872	12.1	249.1	4	52	6	1.1	9
191	Aet-Ole-Ehx-Ole-Ehx-Cme	1199.847	13.5	220.0	4	52	6	1.1	9
192	Apd-Dod-Ehx-Dod-Ehx	937.45	9.6	176.8	4	40	5	2.6	8
193	Apd-Dec-Dec-Dec-Dec	937.45	9.9	176.8	4	40	5	2.6	8
194	Dea-Ole-Ehx-Ole-Ehx	1115.769	14.3	168.0	4	52	5	2.5	8
195	Aad-Ole-Ehx-Ole-Ehx	1101.742	14.1	176.8	4	52	5	2.8	8
196	Frm-Cpr-Ole-Ehx-Ole-Ehx	1127.736	14.8	181.9	4	52	6	0.1	9
197	Frm-Dec-Pet-Dec-Aet-Dec-Pet-Dec	1256.858	12.6	211.3	6	56	8	1.0	11
198	Frm-Ole-Ehx-Pet-Aet-Pet-Ehx-Ole	1421.15	16.8	211.3	6	68	8	1.0	11
199	Frm-Ole-Pet-Ehx-Aet-Ehx-Pet-Ole	1421.15	16.8	211.3	6	68	8	1.0	11
200	Frm-Ole-Ehx-Ole-Ehx	1085.699	14.8	173.7	4	52	5	1.0	9
201	Frm-Imp-Ole-Ehx-Ole-Ehx	1163.817	15.7	160.2	4	52	6	1.0	9
202	Frm-Pip-Ole-Ehx-Ole-Ehx	1180.888	16.2	147.9	4	52	6	1.0	9
203	Frm-Mop-Ole-Ehx-Ole-Ehx	1182.86	15.0	157.1	4	52	6	1.0	9
204	Frm-Mpp-Ole-Ehx-Ole-Ehx	1195.903	15.1	151.1	4	52	6	1.0	9
205	Frm-Aet-Ole-Ehx-Ole-Ehx	1098.742	14.5	170.7	4	52	6	1.0	9
206	Frm-Apr-Ole-Ehx-Ole-Ehx	1112.769	14.8	170.7	4	52	6	1.0	9
207	Frm-Ahx-Ole-Ehx-Ole-Ehx	1154.85	15.8	170.7	4	52	6	1.0	9
208	Frm-Mip-Ole-Ehx-Ole-Ehx	1177.844	15.9	160.2	4	52	6	1.0	9
209	Eea-Ole-Hex-Ole-Ehx	1099.77	15.4	147.8	5	50	5	1.9	9
210	Frm-Imp-Imp-Ole-Ehx-Ole-Ehx	1329.013	15.4	196.2	4	52	7	1.9	9
211	Frm-Ole-Ehx-Imp-Imp-Ole-Ehx	1329.013	15.4	196.2	4	52	7	1.9	9
212	Frm-Ole-Imp-Ehx-Imp-Ole-Ehx	1329.013	15.4	196.2	4	52	7	1.9	9
213	Frm-Ole-Ehx-Imp-Ole-Ehx	1163.817	15.7	160.2	4	52	6	1.0	9
214	Nhe-Ole-Ehx	594.926	6.1	116.0	2	26	3	1.9	6
215	Nhe-Ole-Ehx-Ole	932.474	11.9	156.5	3	44	4	2.6	8
216	Nhe-Ole-Ehx-Ole-Ehx-Ole	1425.307	20.3	197.1	5	70	6	2.6	10
217	Nhe-Ole-Ehx-Ole-Ehx-Ole-Ehx	1578.532	22.4	217.4	6	78	7	2.6	10
218	Nhe-Dec-Dec	542.806	3.5	136.2	2	20	3	2.6	6
219	Nhe-Dec-Dec-Dec	740.128	6.6	156.5	3	30	4	2.6	6

220	Nhe-Dec-Dec-Dec-Dec-Dec	1134.772	12.9	197.1	5	50	6	2.6	8
221	Nhe-Dec-Dec-Dec-Dec-Dec-Dec	1332.094	16.2	217.4	6	60	7	2.6	10

Table S2. Average values of computed parameters for each cluster, and total number of cluster members.

Cluster	Count	Molecular Weight	LogP	Polar Surface Area	Lipid Monomers	Total Lipid Carbons	Total Monomers	Total Charge pH 5.5
1	34	1770.27	9.97	344.40	7.91	68.53	11.18	3.27
2	8	2140.87	15.08	369.49	10.13	88.25	13.25	3.27
3	9	2601.80	16.95	460.67	12.00	104.00	16.33	4.04
4	50	960.93	9.47	172.83	4.12	38.68	5.70	1.14
5	10	638.99	6.05	129.83	2.70	26.20	3.60	1.63
6	3	1039.03	-3.09	330.43	1.33	13.33	8.00	3.69
7	10	1351.56	9.92	256.04	5.90	53.60	8.10	2.83
8	11	1024.71	11.70	172.28	4.00	44.55	5.00	2.63
9	52	1145.53	14.87	173.25	4.08	51.58	5.77	1.32
10	15	1485.88	22.18	202.68	5.20	71.60	6.73	1.65
11	16	1381.06	15.62	212.15	6.56	63.25	8.31	0.84
12	3	2041.31	30.72	251.25	9.00	102.67	10.00	0.69
Full Library	221	1326.47	12.7	118.0	5.52	55.7	7.59	1.9

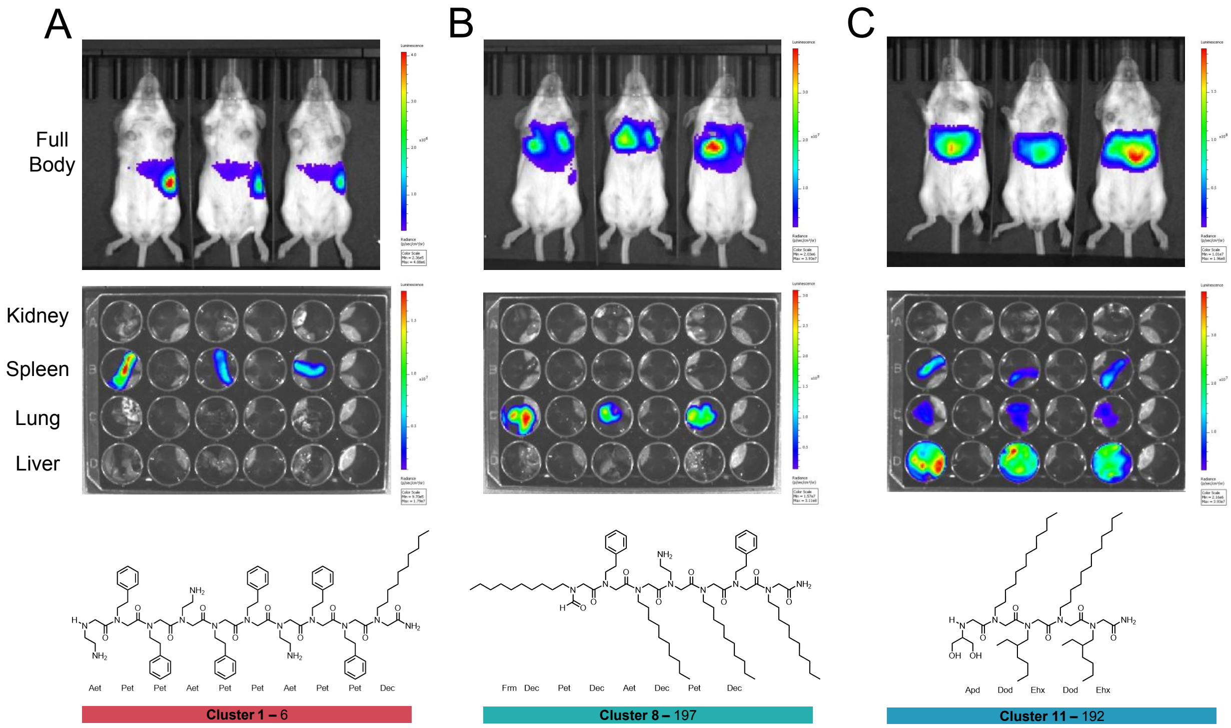

Figure S2. Full-body and ex-vivo bioluminescence images for representative candidates in three different clusters, highlighting differences in biodistribution between cluster biodistribution. All animals were injected IV via the tail vein with 2.5 µg Fluc-encoding mRNA and imaged 6 hours post-injection

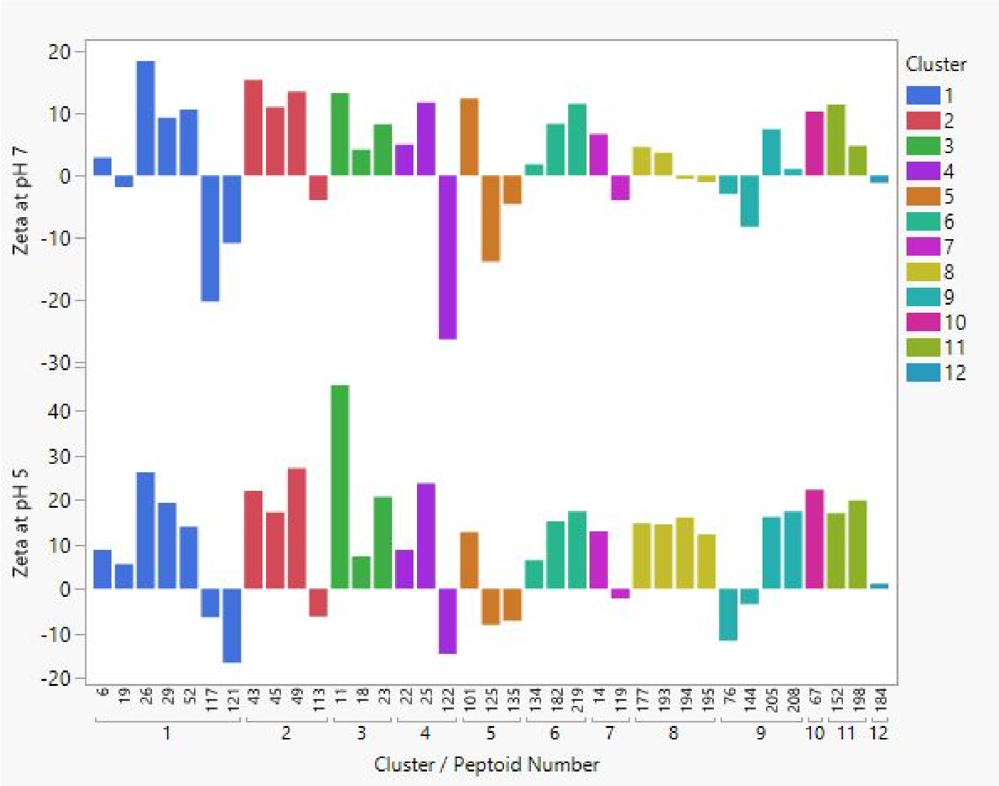

Figure S3. Zeta potential measurements from several candidates from each cluster at pH 5 and 7. All samples were measured on the Malvern Zetasizer at 1 µg/ml mRNA in Zeta Buffer at pH 7.4 or 5.5 (10 mM MES, 10 mM HEPES, 10 mM Ammonium Acetate).

Table S3. Measured physical properties of Nutshell® particles formulated using panel of headgroup variations.

Peptoid	Sequence	Particle Size (nm)	PDI	% mRNA Encapsulation	Hemolysis at pH 5	Hemolysis at pH 7
222	Aet-Dod-Dod-Dod-Dod	61.3	0.206	86.6	-15.1	39.2
223	Apr-Dod-Dod-Dod-Dod	57.3	0.135	97.5	-15.6	39.8
224	Nhe-Dod-Dod-Dod-Dod	65.3	0.196	87.5	24.4	51.1
225	Nhp-Dod-Dod-Dod-Dod	67	0.179	92.2	10.6	37.2
226	Apd-Dod-Dod-Dod-Dod	66.4	0.262	81.16	35.8	59.4
227	Dea-Dod-Dod-Dod-Dod	67.7	0.186	90.5	4.9	12
228	Eae-Dod-Dod-Dod-Dod	62.4	0.175	95.9	-2.4	3.9
229	Nme-Dod-Dod-Dod-Dod	67.1	0.223	92.6	4.9	22.7
230	Nmp-Dod-Dod-Dod-Dod	71.3	0.219	93.1	31.6	65.5
231	Dme-Dod-Dod-Dod-Dod	57.6	0.18	95.4	-12.3	8.2
232	Mip-Dod-Dod-Dod-Dod	63	0.201	98.8	0.7	2.4
233	Mop-Dod-Dod-Dod-Dod	84.6	0.52	86.6	0.6	1

Table S4. Peptoid sequence designs for panel of lipid variations.

Peptoid	Sequence	Total Lipid Monomers	Branched/Unsaturated Monomers	Total Lipid Carbons	Branched/Unsaturated Type
226	Apd-Dod-Dod-Dod-Dod	4	0	48	
234	Apd-Dec-Dec-Dec-Dec	4	0	40	
235	Apd-Dod-Ehx-Dod-Dod-Ehx-Dod	6	2	64	EthylHexyl
236	Apd-Ehx-Dod-Ehx-Ehx-Dod-Ehx	6	4	56	EthylHexyl
237	Apd-Dec-Ehx-Dec-Dec-Ehx-Dec	6	2	56	EthylHexyl
238	Apd-Dod-Ole-Dod	3	1	42	Oleyl
239	Apd-Ole-Dec-Ole-Dec	4	2	56	Oleyl
240	Apd-Ole-Dec-Ole	3	2	46	Oleyl
241	Apd-Ehx-Dec-Dec-Dec-Ehx-Dec-Dec-Dec	8	2	76	EthylHexyl
242	Apd-Ole-Hex-Hex-Hex-Ole-Hex-Hex-Hex	8	2	72	Oleyl
243	Apd-Oct-Hex-Hex-Hex-Hex-Hex-Hex-Oct	8	0	52	
244	Apd-Ehx-Oct-Oct-Oct-Ehx-Oct-Oct-Oct	8	2	64	EthylHexyl
245	Apd-Ehx-Ehx-Hex-Ehx-Ehx-Hex	6	4	44	EthylHexyl
246	Apd-Ehx-Hex-Ehx-Hex-Ehx-Hex-Ehx-Hex	8	4	56	EthylHexyl
247	Apd-Hex-Hex-Hex-Hex-Hex-Hex-Hex-Hex	8	0	48	
248	Apd-Ehx-Dod-Hex-Hex-Ehx-Dod-Hex-Hex	8	2	64	EthylHexyl
249	Apd-Dod-Dod-Dod-Dod-Dod-Dod	6	0	72	
250	Apd-Ehx-Dod-Dod-Hex-Ehx-Dod-Dod-Hex	8	2	76	EthylHexyl
251	Apd-Ole-Oct-Ole	3	2	44	Oleyl
252	Apd-Dod-Dec-Dod-Dec	4	0	44	
253	Apd-Dod-Ehx-Dod-Ehx	4	2	40	EthylHexyl
254	Apd-Ehx-Ehx-Ehx-Ehx	4	4	32	EthylHexyl
255	Apd-Oct-Oct-Hex-Oct-Oct-Hex	6	0	44	
256	Apd-Oct-Oct-Dec-Oct-Oct-Dec	6	0	52	
257	Apd-Dec-Dod-Dec-Dec-Dod-Dec	6	0	64	
258	Apd-Oct-Dec-Ehx-Oct-Dec-Ehx	6	2	52	EthylHexyl
259	Apd-Dec-Dec-Ehx-Dec-Dec-Ehx	6	2	56	EthylHexyl
260	Apd-Dod-Dod-Ehx-Dod-Dod-Ehx	6	2	64	EthylHexyl
261	Apd-Oct-Dec-Hex-Oct-Dec-Hex	6	0	48	
262	Apd-Dod-Dod-Oct-Dod-Dod-Oct	6	0	64	
263	Apd-Hex-Dod-Ehx-Hex-Dod-Ehx	6	2	52	EthylHexyl
264	Apd-Oct-Dod-Ehx-Oct-Dod-Ehx	6	2	56	EthylHexyl
265	Apd-Ole-Oct-Ole-Oct	4	2	52	Oleyl
266	Apd-Dod-Ehx-Ehx-Dod-Ehx-Ehx	6	4	56	EthylHexyl

Table S5. Measured particle properties of Nutshells® formulated using panel of lipid variations.

Peptoid	Sequence	Particle Size (nm)	PDI	% Encap.	Zeta Potential	pKa (TNS)	Hemolysis at pH 5	Hemolysis at pH 7
226	Apd-Dod-Dod-Dod-Dod	66.4	0.262	81.2	2.7	5.406	35.8	59.5
234	Apd-Dec-Dec-Dec-Dec	181.4	Multimodal	56.4	-0.676	6.418	7.8	23.9
235	Apd-Dod-Ehx-Dod-Dod-Ehx-Dod	270.5	Multimodal	55.9	-7.48	4.883	0.5	-0.1
236	Apd-Ehx-Dod-Ehx-Ehx-Dod-Ehx	75.7	0.15	94.2	-5.38	6.068	4.5	2.9
237	Apd-Dec-Ehx-Dec-Dec-Ehx-Dec	2058.2	Multimodal		-6.37	4.595	1.2	0.2
238	Apd-Dod-Ole-Dod	631.3	Multimodal	12.3				
239	Apd-Ole-Dec-Ole-Dec	71.3	0.39	85.9	-0.785	6.373	21.5	60.6
240	Apd-Ole-Dec-Ole	62.3	0.13	86.3	-2.31	6.283	6.1	23.8
241	Apd-Ehx-Dec-Dec-Dec-Ehx-Dec-Dec-Dec	90.5	0.04	46.6	-12.4	5.786	0.5	-0.1
242	Apd-Ole-Hex-Hex-Hex-Ole-Hex-Hex-Hex	94.9	0.14	95.2	-1.6	5.906	1.1	0.1
243	Apd-Oct-Hex-Hex-Hex-Hex-Hex-Hex-Oct	122.5	0.3	97	-2.71	6.052	1.1	0
244	Apd-Ehx-Oct-Oct-Oct-Ehx-Oct-Oct-Oct	106.6	0.111	66.1				
245	Apd-Ehx-Ehx-Hex-Ehx-Ehx-Hex	91.4	0.32	97.4	-8.68	6.018	52.1	1.5
246	Apd-Ehx-Hex-Ehx-Hex-Ehx-Hex-Ehx-Hex	122.4	0.102	82	-5.17	5.686	1.8	0.6
247	Apd-Hex-Hex-Hex-Hex-Hex-Hex-Hex-Hex	2228.3	Multimodal	57.3	-3.14	6.825	9.1	0.2
248	Apd-Ehx-Dod-Hex-Hex-Ehx-Dod-Hex-Hex	106.6	0.111	84.5	-8	5.909	0.9	0.6
249	Apd-Dod-Dod-Dod-Dod-Dod-Dod	93.1	0.24	30.6	-3.58	5.554	4.1	2.2
250	Apd-Ehx-Dod-Dod-Hex-Ehx-Dod-Dod-Hex	96.7	0.11	50.3	-17.8	5.656	2.1	0.3
251	Apd-Ole-Oct-Ole	65.4	0.18	70.1	3.41	5.773	16.7	33.3
252	Apd-Dod-Dec-Dod-Dec	51.5	0.179	83.3	4.24	5.888	58.9	81.5
253	Apd-Dod-Ehx-Dod-Ehx	1269.5	Multimodal	2.3				
254	Apd-Ehx-Ehx-Ehx-Ehx	66.9	0.12	97.6	-1.59	6.43	100.4	99.9
255	Apd-Oct-Oct-Hex-Oct-Oct-Hex	2220.3	Multimodal	24.3			94.6	94.7
256	Apd-Oct-Oct-Dec-Oct-Oct-Dec	421.9	Multimodal	66.1				
257	Apd-Dec-Dod-Dec-Dec-Dod-Dec	107.6	0.214	87.9	1.84	5.48	12.4	9.1
258	Apd-Oct-Dec-Ehx-Oct-Dec-Ehx	86.6	0.089	82.2	-5.98	5.844	15.1	5.6
259	Apd-Dec-Dec-Ehx-Dec-Dec-Ehx	92.7	0.094	81.9	-7.04	6.044	4.8	1.3
260	Apd-Dod-Dod-Ehx-Dod-Dod-Ehx	82.6	0.097	87.7	-1.41	6.084	17.3	2.1
261	Apd-Oct-Dec-Hex-Oct-Dec-Hex	692.4	Multimodal	23.3				
262	Apd-Dod-Dod-Oct-Dod-Dod-Oct	165.8	1	-1.8	-2.12	5.935	0.9	0.4
263	Apd-Hex-Dod-Ehx-Hex-Dod-Ehx	118.4	0.093	71.2	-9.58	6.374	11.8	27.6
264	Apd-Oct-Dod-Ehx-Oct-Dod-Ehx	90.7	0.082	76.1	-5.88	5.937	4.5	5.1
265	Apd-Ole-Oct-Ole-Oct	98.1	0.194	76.4	2.39	6.936	51.6	95.8
266	Apd-Dod-Ehx-Ehx-Dod-Ehx-Ehx	80.8	0.1	86.9	-11.2	6.523	5.8	2

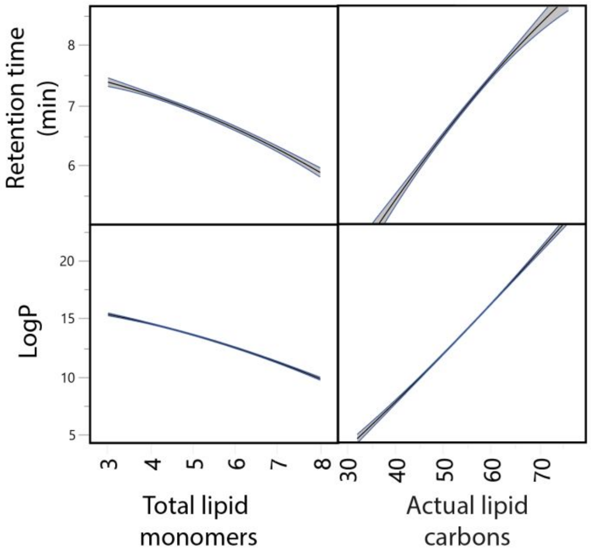

Figure S4. Impact of lipid structure on peptoid retention time and predicted LogP. Model fit generated using JMP.

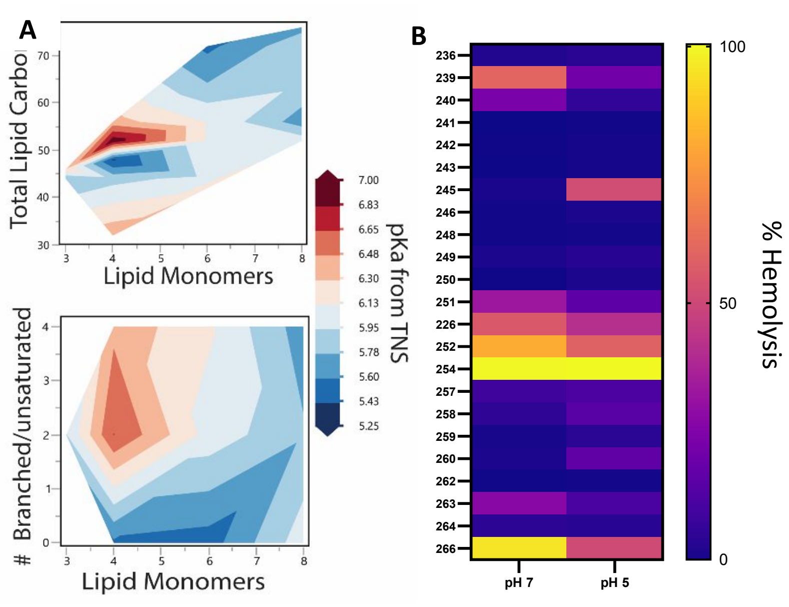

Figure S5. Additional physical characterization of the peptoids with parameterized lipid block variations. Paired contour plots show how lipid parameters impact particle pKa. C) Heat map of RBC hemolysis at pH 7 and 5.

Response Log10[Total Flux [p/s]]

Actual by Predicted Plot

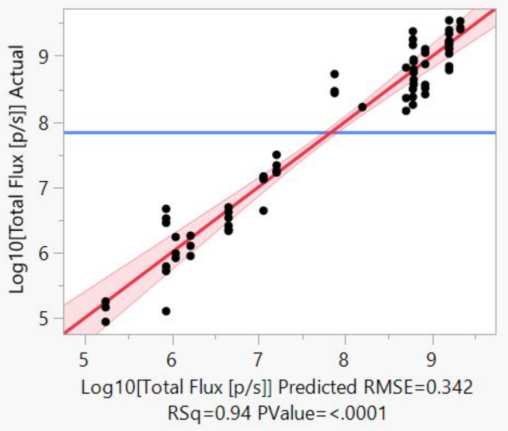

Effect Summary

Source	Logworth	PValue
Actual Lipid Carbons*Actual Lipid Carbons	11.103	0.00000
Unsaturated/Branched Monomers*Unsaturated/Branched Monomers	6.611	0.00000
Actual Lipid Carbons*Unsaturated/Branched Type	6.415	0.00000
Unsaturated/Branched Type	6.150	0.00000 ^
Unsaturated/Branched Monomers*Unsaturated/Branched Type	4.596	0.00003
Total Lipid Monomers	4.018	0.00010
Actual Lipid Carbons	3.396	0.00040 ^
Total Lipid Monomers*Unsaturated/Branched Type	2.407	0.00392
Unsaturated/Branched Monomers	1.546	0.02845 ^
Total Lipid Monomers*Total Lipid Monomers	0.760	0.17370

Remove Add Edit Undo ☐ FDR ('^' denotes effects with containing effects above them)

Prediction Profiler

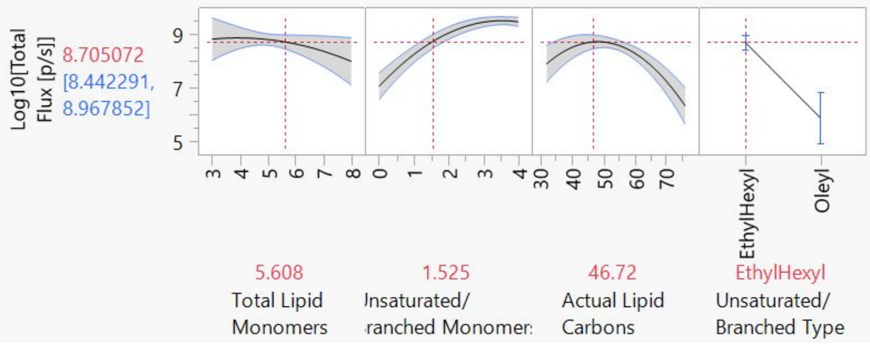

Figure S6. Multivariate model fit for expression based on lipid parameters. Graphics and fittings generated using JMP.

Table S6. Measured physical properties of Nutshell® particles formulated using peptoids with variations in lipid monomer sequence.

Compound	Sequence	Particle Size (nm)	PDI	% Encapsulation	Zeta Potential	pKa (TNS)
236	Apd-Ehx-Dod-Ehx-Ehx-Dod-Ehx	75.7	0.150	94.2	-5.38	6.07
266	Apd-Dod-Ehx-Ehx-Dod-Ehx-Ehx	80.8	0.100	86.95	-11.2	6.52

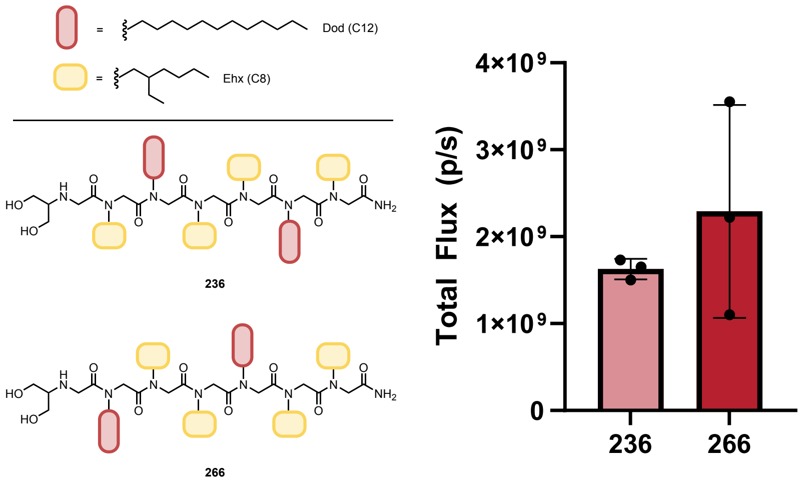

Figure S7. In vivo luciferase expression for peptoids with similar lipid monomer composition but different sequences. 2.5 µg IV dose of Fluc mRNA containing Nutshell® particles in Balb/c mice with Bioluminescence imaging of organs after 6 hrs. Data = mean ± SD. N = 3.

Table S7. Particle properties for Nutshells® formulated with anti-RSV mRNA

Peptoid	Sequence	Diameter (nm)	PDI	% mRNA encapsulation
236	Apd-Ehx-Dod-Ehx-Ehx-Dod-Ehx	80.2	0.078	96.1
245	Apd-Ehx-Ehx-Hex-Ehx-Ehx-Hex	67.6	0.065	95.8
263	Apd-Hex-Dod-Ehx-Hex-Dod-Ehx	71.8	0.087	88.5
264	Apd-Oct-Dod-Ehx-Oct-Dod-Ehx	71.7	0.09	92.3

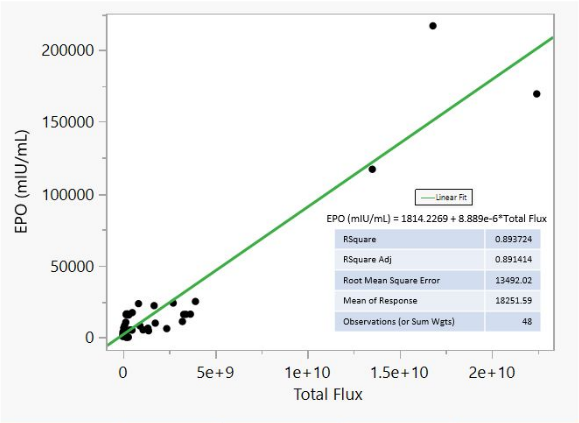

	Value	Lower 95%	Upper 95%	Signif. Prob
Correlation	0.94537	0.904083	0.969173	<.0001*
Covariance	1.69e+14			
Count	48			

Figure S8. Strong correlation between secreted protein (hEPO) and Fluc observed for peptoid-based lipid nanoparticles. Serum expression of hEPO after 24 hrs was compared to 6hr luciferase expression for matched peptoid formulations.

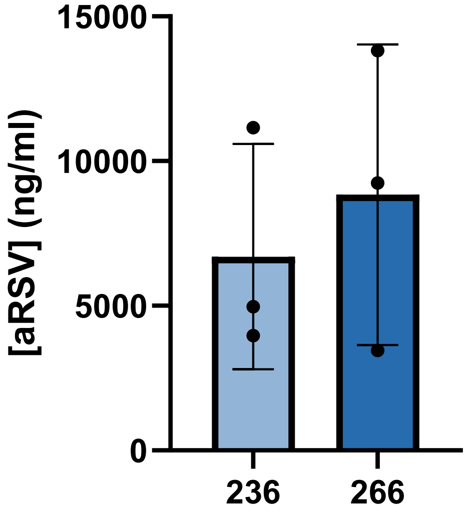

Figure S9. In vivo serum levels of anti-RSV antibody for Nutshell® particles made with peptoids with similar lipid monomer identity but different sequences. Balb/c mice (n = 3) were treated IV with 0.3 mg/kg of aRSV mRNA and serum collected after 24 hours. Data represents mean ± SD.

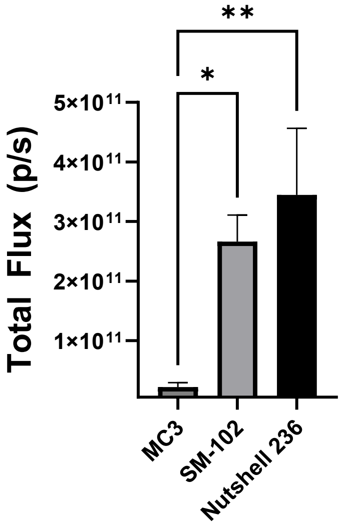

Figure S10. Comparison of in vivo Fluc expression of Nutshell® 236 to benchmark LNPs. In vivo Fluc expression of Nutshell 236 is comparable to SM-102 and greater than MC3. All conditions were treated in Balb/c mice at 0.75 mg/kg and luminescence measured at 6h. Students t-test used to calculate significance (* $p < 0.05$).

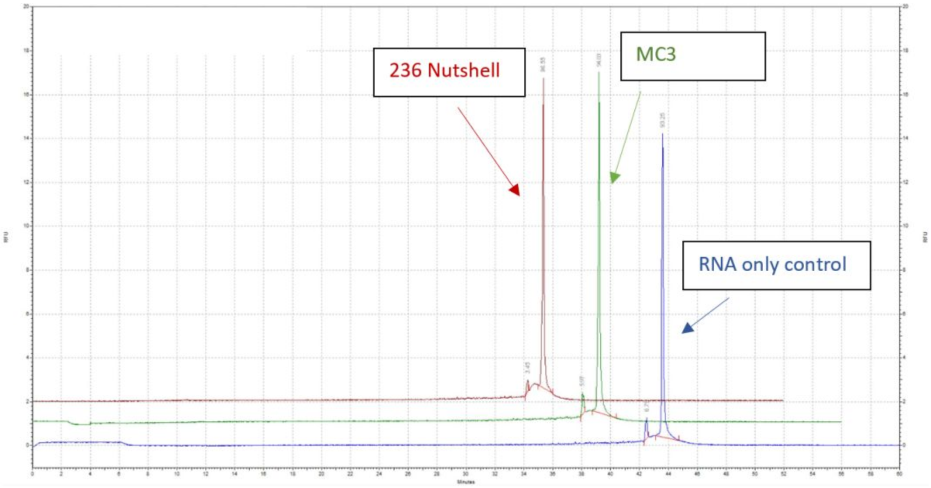

Figure S11. Integrity analysis of mRNA from deformulated Nutshell 236 and MC3 stored for > 6 months at -80 C compared to a fresh mRNA control. Lack of low molecular weight peaks indicates that mRNA integrity is retained during this storage period. Purity analysis was completed by capillary electrophoresis on the PA800 Plus Pharmaceutical Analysis System using the RNA 9000 Purity & Integrity Kit by Agilent Technologies.

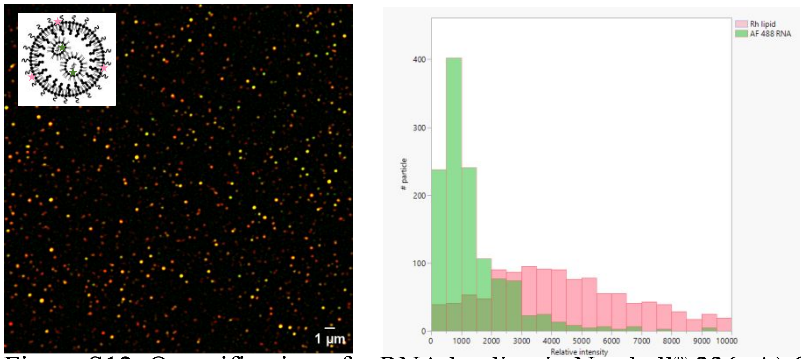

Figure S12. Quantification of mRNA loading in Nutshell® 236. A) Overlay of fluorescence micrographs showing colocalization (yellow) of Rh-DSPE lipid (red) with Alexa Fluor 488 mRNA (green) in Nutshell® 236. B) Quantification of lipid and RNA channel intensity from colocalization.

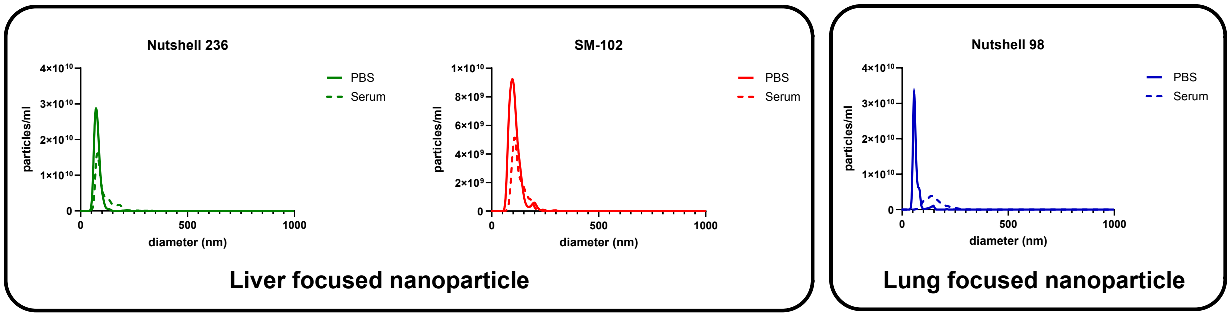

Figure S13. NTA analysis of nanoparticles in the presence of serum reveals differences in behavior associated with biodistribution. Nutshell® 236 and SM-102 both show peak broadening and a shift towards higher populations in the presence of serum, but the main peak population is maintained. Lung focused Nutshell 98 shows a more prominent shift with the entire main peak shifting to higher size and broadening.

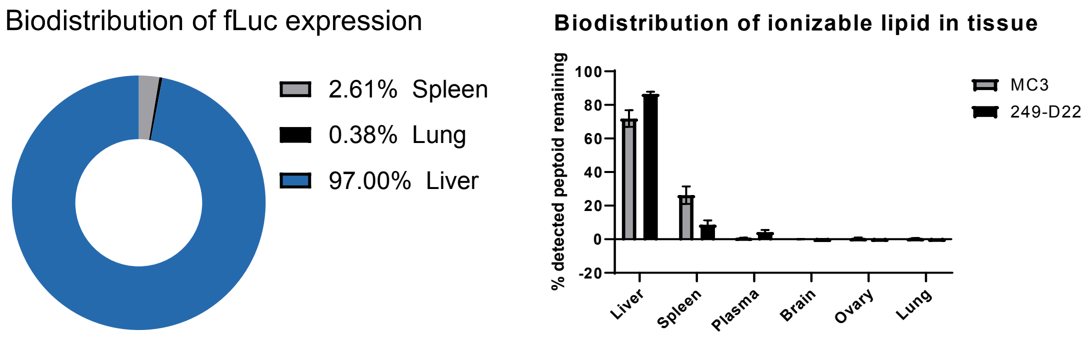

Figure S14. Distribution of Fluc expression aligns with ionizable lipid localization in Balb/c mice. Distribution of Fluc expression shows greater than 97% liver selectivity at 0.75 mg/kg dose of Nutshell 236. Biodistribution of ionizable lipid in major organs 6 hrs post dose for Nutshell 236 and MC3 LNP. Nutshell 236 shows higher localization of peptoid in liver compared to MC3 benchmark.

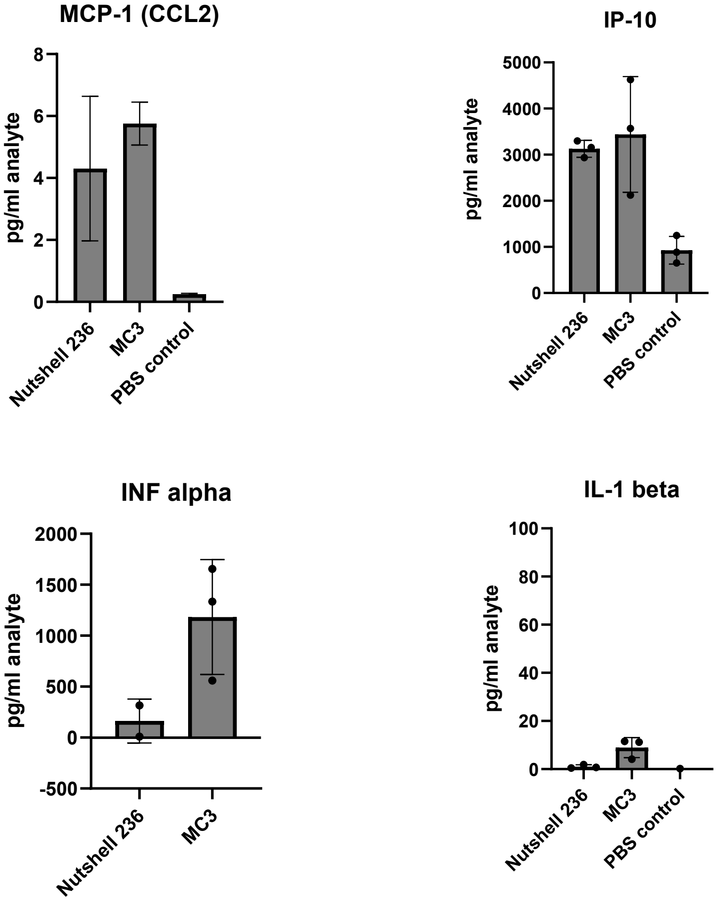

Figure S15. 6hr additional cytokine analytes from 0.75 mg/kg Fluc mRNA dose show no elevation compared to MC3 (benchmark ionizable lipid). Data = mean ± SD .

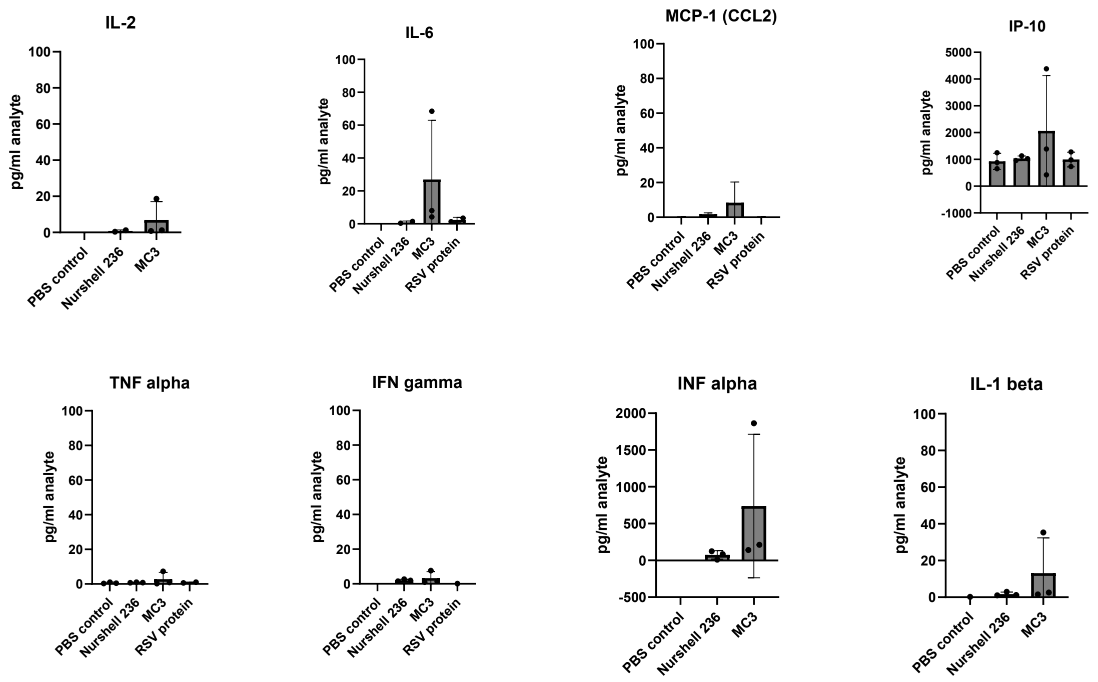

Figure S16. 24hr cytokines analytes from 0.75 mg/kg aRSV mRNA dose in Balb/c mice show no elevation above benchmark lipid (MC3) or protein control.

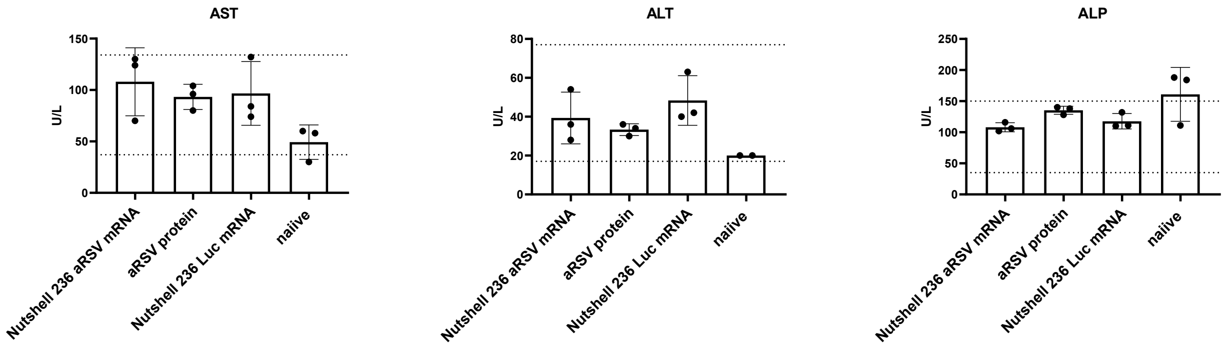

Figure S17. AST, ALT, and ALP levels 24hr post dose in Balb/c serum (n=3). One way ANOVA analysis shows no difference among all groups. All mRNA groups were dosed at 0.75mg/kg while protein group was dosed at 3mg/kg to achieve matched serum titers. Changing from Fluc to aRSV cargo in Nutshell formulation had no impact.

II. Supplemental Methods:

Materials:

Acetonitrile (ACN), N,N-dimethylformamide (DMF), N,N'-diisopropylcarbodiimide (DIC), bromoacetic acid, trifluoroacetic acid, 2-amino-1,3-propanediol, and n-decylamine were purchased from Fisher Scientific. 3-methoxypropan-1-amine, tert-butyl N-(3-aminopropyl)carbamate, and 3-morpholinopropylamine were purchased from Enamine LTD. 2-Ethylhexylamine was purchased from VWR. 2-aminoethan-1-ol, 1-(3-aminopropyl)imidazole, 2-ethylaminoethanol, diethanolamine, and 4-methyl piperidine were purchased from TCI. High-load Rink Amide MBHA Resin (0.6 mmol/g) was purchased from Gyros Protein Technologies. IgG Human Elisa Kit (cat: BMS2091), Quant-it RiboGreen RNA Reagent and TE buffer were purchased from Thermo Fisher. Biotage Sfar Bio C4 Duo 300 A flash chromatography columns were purchased from Biotage. All other chemical reagents were purchased from Millipore Sigma (Burlington, MA).

HPLC:

The retention time and the purity of synthesized peptoids were detected using HPLC-UV on a 1.7 μm ACQUITY UPLC protein BEH C4 protein column (Waters, pore size: 300 Å, 2.1(I.D.) × 100 mm) via a running gradient at a temperature of 55 °C with a mixture of Solvent A and Solvent B (Solvent A: water/isopropanol (98/2 with 0.1% v/v trifluoroacetic acid), Solvent B: Isopropanol/acetonitrile (80/20 with 0.1 trifluoroacetic acid)) from 35 to 90% solvent B in 8 min, followed by a 4 min wash with 100% solvent B and return to 35% solvent B at a flow rate of 0.4 ml/min. Detection was accomplished using UV absorption at 214 nm, and an injection volume of 3 μL was used for all samples.

Particle Size and Dispersity:

The size and polydispersity (PDI) of nutshells was measured by dynamic light scattering in PBS using DynaPro Plate Reader III (Wyatt Technology, Santa Barbara, CA). The zeta potential of Nutshells® was determined using Nano-ZS particle analyzer (Malvern Instruments, Malvern, UK). Zeta sample was diluted to 30 μM total lipid in a weakly buffered solution of 10 mM HEPES, 10 mM MES, 10 mM ammonium acetate at pH 7. Measurements we collected in duplicate for over 30 runs with the voltage manually set to 120 mV

% mRNA Encapsulation

The mRNA concentration and the percentage of encapsulated mRNA were determined using fluorescent-based Quant-iT RiboGreen RNA Reagent Kit (Life Technologies, Waltham, MA). To determine the mRNA concentration, particles were incubated with 2% Triton X-100 for 10 min to release entrapped mRNA, followed by addition of RiboGreen RNA reagent. The fluorescence intensity of mRNA-RiboGreen reagent complex (excitation at 485 nm and emission at 530 nm) was detected using a SpectraMax iD3 microplate reader (Molecular Devices, San Jose, CA, USA). The ratio of fluorescence without and with Triton X-100 were used to calculate the percentage of encapsulated mRNA.

TNS assay:

The apparent particle pKa was assessed by TNS fluorescence assay. Briefly, particles were diluted to 130 µM total lipid in TNS buffer (10 mM HEPES, 10 mM Ammonium Acetate, 10 mM MES, 140 mM NaCl) ranging in pH 3-11 for 10 minutes with 10 mM of TNS probe. The fluorescence was measured at room temperature in a Molecular Devices plate reader with (ex 320 nm/em445 nm). Fluorescence intensity was normalized to the minimal and maximal values and the pKa was determined by a 3-parameter logistic fit to determine the half maximum of the curve.

Hemolysis:

RBC hemolysis was used as a metric for fusogenicity of LNPs at low pH. RBCs were washed with 2x PBS by pelleting at 800x g and resuspended to 5% v/v in either 20 mM citrate with 140mM NaCl (pH 5.5) or PBS. Particles were diluted to 150 mM total lipid in well with 5% RBC solution and incubated at 37 C for 45 min. Supernatant was collected, and UV absorbance read on a Spectramax i3x at 425 nm. % Hemolysis was calculated using the following equation:

$\% \, Hemolysis = 100 * \frac{A_{Sample}}{A_{Triton}}$ Where A_{Sample} is the absorbance at 425 nm of test well at a given pH after subtracting the background of buffer + RBC only and A_{Triton} is the fully lysed condition at a given pH with 0.1%% Triton-X after background subtraction

Fluorescence Imaging:

For fluorescent cargo loading measurements, DBCO Alexa488 was conjugated to the 3' end of the polyA tail of uc mRNA that was modified by the addition of 2' azido-ATP using yeast Poly(A) polymerase. Ampur and ethanol precipitation we used to further purify and the conjugation efficiency was measured by comparing the RNA UV absorbance to that of the dye max absorbance. Labeled RNA was used when formulating Nutshell 236 along with 0.1 mol% Rh-DHPE. Particles were immobilized on a glass slide and images using a Zeiss Elyra inverted widefield microscope equipped with 63x oil immersion objective and 488, 561, and 647 nm laser excitation. Image processing and colocalization was completed with ImageJ and JMP for generating data summary.

Nanoparticle Tracking Analysis (NTA):

Nanoparticle tracking analysis was completed using the Malvern Panalytical Nanosight 3.4 Instrument with capture settings: camera level= 12; slider gain = 125; frame number 1498; FPS = 25; 3 x 60s streams; and detection threshold = 20. Particles were diluted 1500x from an initial starting concentration of 0.05mg/ml. Plots shown are an average of the 3 measurements completed for each condition.

Cytokine quantification:

Cytokines were detected in serum using a Mouse Immune Monitoring 48-Plex ProcartaPlex Panel (Invitrogen EPX480-20834-901) bead-based immunoassay according to manufacturer's instructions and data was collected on a Luminex 200 System.

Peptoid Biodistribution by Mass Spectrometry:

Quantity of peptoids in mice tissues was determined via high-performance liquid chromatography coupled with Time-of-flight mass spectrometer (HPLC-TOF). Solid tissue homogenate samples were prepared by resuspending 25 mg tissue in 500 mL extraction solvent (IPA/MeOH=8/2) containing an internal standard followed by bead homogenization (45 sec per cycle for 3 cycles). Homogenized samples were centrifuged at 12,000 rpm for 8 min at 6 °C. The resulting supernatant was collected for further LC analysis. Serum samples were diluted with extraction buffer (200 pg/uL internal standard) by 20X followed by centrifugation at 12, 000 rpm for 8 min to collect the supernatant. 5 µL of the resulting supernatant sample from solid or liquid tissue was injected into a BEH C8 column on an Agilent 1290 Inifinity II liquid chromatograph coupled with an Agilent 6230 LC-TOF system. Concentrations of peptoids were determined by a linear calibration curve (linear range:0.2-50 µg/g for solid tissue and 10-2500 pg/µL for liquid tissue).

III. Synthetic Characterization Data

Table S8: Synthetic characterization data for peptoids tested as part of cluster evaluation. [a] LCMS methods used are defined as:

Method A: Waters Acquity UPLC Peptide BEH C8 column; 5-95% Gradient over 7 minutes; Solvent A = H_2O + 0.1 vol% TFA, Solvent B = MeCN + 0.1 vol% TFA

Method B: Waters Acquity UPLC Peptide BEH C8 column; 5-95% Gradient over 13 minutes; Solvent A = H_2O + 0.1 vol% TFA, Solvent B = MeCN + 0.1 vol% TFA

Method C: Waters Acquity UPLC Peptide BEH C8 column; Solvent A = 60% MeCN/40%H_2O + 0.1 vol% Formic Acid, Solvent B = 20% iPrOH/80% MeCN + 0.1 vol% Formic Acid

Peptoid	Monomer Sequence	Exact Mass (Da)	Found Mass (Da)	LCMS Retention Time (min)	LCMS Method Comment[a]
1	Aet-Pet-Pet-Aet-Pet-Pet-Aet-Pet-Pet-Oct-Oct	1622.02	1623.94	4.43	A
2	Aet-Pet-Pet-Aet-Pet-Pet-Aet-Pet-Pet-Dec-Dec	1678.08	1679.10	4.75	A
3	Aet-Pet-Pet-Aet-Pet-Pet-Aet-Pet-Pet-Dod-Dod	1734.14	1735.30	5.03	A
4	Aet-Mpe-Mpe-Aet-Mpe-Mpe-Aet-Mpe-Mpe-Dod-Dod	1914.20	1915.40	4.97	A
5	Aet-Pet-Pet-Aet-Pet-Pet-Aet-Pet-Pet-Ode-Ode	1902.33	1904.30	5.88	A
6	Aet-Pet-Pet-Aet-Pet-Pet-Aet-Pet-Pet-Dec	1480.90	1482.10	4.17	A
7	Aet-Pet-Pet-Aet-Pet-Pet-Aet-Pet-Pet-Dec-Dec-Dec	1875.26	1876.40	5.31	A
8	Aet-Pet-Pet-Aet-Pet-Pet-Aet-Pet-Pet-Dec-Dec-Dec-Dec	2072.43	2073.40	5.62	A
9	Aet-Pet-Pet-Dec-Dec	833.61	834.79	4.54	A
10	Aet-Pet-Pet-Aet-Pet-Pet-Dec-Dec	1255.85	1257.73	4.67	A
11	Aet-Pet-Pet-Aet-Pet-Pet-Aet-Pet-Pet-Aet-Pet-Pet-Dec-Dec	2100.31	2101.50	4.78	A
12	Aet-Pet-Pet-Aet-Pet-Pet-Aet-Pet-Pet-Aet-Pet-Pet-Aet-Pet-Pet-Dec-Dec	2522.54	2524.50	4.81	A
13	Aet-Pet-Aet-Pet-Aet-Pet-Dec-Dec	1194.83	1196.70	4.13	A
14	Aet-Aet-Aet-Dec-Dec	711.57	712.70	3.43	A
15	Aet-Aet-Aet-Aet-Aet-Dec-Dec	911.70	912.87	3.23	A
16	Aet-Pet-Pet-Dec-Aet-Pet-Pet-Dec-Aet-Pet-Pet	1678.08	1680.10	4.6	A
17	Dec-Aet-Pet-Pet-Aet-Pet-Pet-Aet-Pet-Pet-Dec	1678.08	1679.30	4.6	A
18	Aet-Pet-Pet-Aet-Pet-Pet-Aet-Pet-Pet-Dec-Dec-Dec-Dec-Dec	2269.61	2290.70	5.96	A
19	Aet-Pet-Pet-Aet-Pet-Pet-Dec-Dec-Dec-Dec-Dec	1847.38	1849.21	6.02	A
20	Aet-Pet-Pet-Aet-Pet-Pet-Aet-Pet-Pet-Aet-Pet-Pet-Dec-Dec-Dec-Dec-Dec	2691.84	2694.10	5.88	A
21	Aet-Pet-Pet-Aet-Pet-Pet-Aet-Pet-Pet-Dec-Dec-Dec-Dec-Dec-Dec	2466.79	2469.62	6.2	A
22	Aet-Pet-Pet-Aet-Pet-Pet-Aet-Pet-Pet-Dec-Dec-Dec-Dec-Dec-Dec-Dec	2663.97	2666.70	6.42	A
23	Aet-Pet-Pet-Aet-Pet-Pet-Aet-Pet-Pet-Dec-Ehx-Dec-Ehx	2016.37	2018.02	5.41	A
24	Mae-Pet-Pet-Mae-Pet-Pet-Mae-Pet-Pet-Dec-Dec-Dec-Dec	2114.48	2116.41	5.63	A

25	Mae-Pet-Pet-Mae-Pet-Pet-Mae-Pet-Pet-Mae-Pet-Pet-Dec-Dec-Dec-Dec	2550.73	2553.77	5.58	A
26	Mae-Pet-Pet-Mae-Pet-Pet-Mae-Pet-Pet-Dec-Dec	1720.12	1721.81	4.76	A
27	Apr-Pet-Pet-Apr-Pet-Pet-Apr-Pet-Pet-Dec-Dec-Dec-Dec	2114.48	2116.40	5.58	A
28	Apr-Pet-Pet-Apr-Pet-Pet-Apr-Pet-Pet-Apr-Pet-Pet-Dec-Dec-Dec-Dec	2550.73	2552.81	5.52	A
29	Apr-Pet-Pet-Apr-Pet-Pet-Apr-Pet-Pet-Dec-Dec	1720.12	1722.11	4.7	A
30	Mpi-Pet-Pet-Mpi-Pet-Pet-Mpi-Pet-Pet-Dec-Dec-Dec-Dec	2234.57	2236.66	5.54	A
31	Mpi-Pet-Pet-Mpi-Pet-Pet-Mpi-Pet-Pet-Mpi-Pet-Pet-Dec-Dec-Dec-Dec	2710.85	2713.72	5.46	A
32	Mpi-Pet-Pet-Mpi-Pet-Pet-Mpi-Pet-Pet-Dec-Dec	1840.22	1842.20	4.64	A
33	Mpl-Pet-Pet-Mpl-Pet-Pet-Mpl-Pet-Pet-Dec-Dec-Dec-Dec	2192.53	2194.43	5.65	A
34	Mpl-Pet-Pet-Mpl-Pet-Pet-Mpl-Pet-Pet-Mpl-Pet-Pet-Dec-Dec-Dec-Dec	2654.79	2656.74	5.6	A
35	Mpl-Pet-Pet-Mpl-Pet-Pet-Mpl-Pet-Pet-Dec-Dec	1798.17	1800.27	4.79	A
36	Aet-Dec-Dec-Dec-Dec	905.80	908.00	5.39	A
37	Apr-Dec-Dec-Dec-Dec	919.82	921.60	5.36	A
38	Nhp-Dec-Dec-Dec-Dec	920.80	922.90	5.62	A
39	Spm-Dec-Dec-Dec-Dec	976.88	978.00	5.23	A
40	Bpp-Dec-Dec-Dec-Dec	1045.93	1047.70	5.16	A
41	Tae-Dec-Dec-Dec-Dec	1033.93	1035.10	5.23	A
42	Imp-Dec-Dec-Dec-Dec	970.83	972.60	5.39	A
43	Aet-Aet-Pet-Pet-Dec-Dec-Dec-Dec	1328.03	1330.00	5.51	A
44	Apr-Aet-Pet-Pet-Dec-Dec-Dec-Dec	1342.05	1343.90	5.43	A
45	Nhp-Aet-Pet-Pet-Dec-Dec-Dec-Dec	1343.03	1345.00	5.62	A
46	Spm-Aet-Pet-Pet-Dec-Dec-Dec-Dec	1399.11	1401.10	5.35	A
47	Bpp-Aet-Pet-Pet-Dec-Dec-Dec-Dec	1468.17	1470.20	5.33	A
48	Tae-Aet-Pet-Pet-Dec-Dec-Dec-Dec	1456.17	1457.30	5.38	A
49	Imp-Aet-Pet-Pet-Dec-Dec-Dec-Dec	1393.06	1395.10	5.42	A
50	Aet-Aet-Pet-Pet-Aet-Pet-Pet-Dec-Dec-Dec-Dec	1750.27	1752.30	5.51	A
51	Apr-Aet-Pet-Pet-Aet-Pet-Pet-Dec-Dec-Dec-Dec	1764.28	1766.40	5.48	A
52	Nhp-Aet-Pet-Pet-Aet-Pet-Pet-Dec-Dec-Dec-Dec	1765.27	1767.30	5.61	A
53	Spm-Aet-Pet-Pet-Aet-Pet-Pet-Dec-Dec-Dec-Dec	1821.34	1824.40	5.4	A
54	Bpp-Aet-Pet-Pet-Aet-Pet-Pet-Dec-Dec-Dec-Dec	1890.40	1892.50	5.38	A
55	Tae-Aet-Pet-Pet-Aet-Pet-Pet-Dec-Dec-Dec-Dec	1878.40	1880.50	5.42	A
56	Imp-Aet-Pet-Pet-Aet-Pet-Pet-Dec-Dec-Dec-Dec	1815.29	1817.30	5.47	A
57	Aet-Aet-Pet-Pet-Aet-Pet-Pet-Dec-Ehx-Dec-Ehx	1694.20	1697.20	5.21	A
58	Apr-Aet-Pet-Pet-Aet-Pet-Pet-Dec-Ehx-Dec-Ehx	1708.22	1710.30	5.23	A
59	Nhp-Aet-Pet-Pet-Aet-Pet-Pet-Dec-Ehx-Dec-Ehx	1709.20	1711.30	5.36	A
60	Spm-Aet-Pet-Pet-Aet-Pet-Pet-Dec-Ehx-Dec-Ehx	1765.28	1767.30	5.14	A
61	Bpp-Aet-Pet-Pet-Aet-Pet-Pet-Dec-Ehx-Dec-Ehx	1834.33	1836.40	5.12	A
62	Tae-Aet-Pet-Pet-Aet-Pet-Pet-Dec-Ehx-Dec-Ehx	1822.33	1823.40	5.17	A
63	Imp-Aet-Pet-Pet-Aet-Pet-Pet-Dec-Ehx-Dec-Ehx	1759.23	1761.20	5.22	A
64	Aet-Dod-Dod-Dod-Dod	1017.93	1018.70	5.93	A

65	Aet-Dod-Dod-Dod-Dod-Dod	1243.14	1244.00	6.33	A
66	Aet-Dod-Dod-Dod-Dod-Dod-Dod	1468.35	1469.30	6.61	A
67	Aet-Aet-Dod-Dod-Dod-Dod-Dod-Dod	1568.41	1569.40	6.47	A
69	Aet-Ole-Ole-Ole-Ole	1346.24	1347.30	6.72	A
70	Aet-Dod-Ehx-Dod-Ehx	905.80	907.60	5.45	A
71	Aet-Dod-Ole-Dod-Ole	1182.08	1183.10	6.37	A
72	Frm-Dod-Dod-Aet-Dod-Dod	1045.92	1046.70	6.13	A
74	Frm-Dod-Dod-Apr-Apr-Dod-Dod	1174.02	1147.02	6.039	C
76	Eed-Dod-Dod-Dod-Dod	1073.99	1075.05	6.545	C
77	Eed-Ole-Ole-Ole-Ole	1402.30	1403.30	8.986	C
84	Aet-Ole-Ehx-Ole-Ehx	1069.96	1070.00	5.91	A
95	Aet-CprDec-Ehx-Dec-Ehx	978.78	979.85	6.019	C
96	Aet-Cpr-Cpr-Dec-Ehx-Dec-Ehx	1107.82	991.86	5.902	C
97	Aet-Dod-Dod-Dod-Ehx	961.86	962.50	9.59	B
98	Apr-Dod-Ehx-Dod-Ehx	919.82	921.00	9.48	B
99	Aet-Cym-Dod-Ehx-Dod-Ehx	1058.92	1059.70	9.83	B
100	Aet-Dod-Ehx-Cym-Dod-Ehx	1058.92	1059.70	9.52	B
101	Pip-Dod-Ehx-Dod-Ehx-Dod	987.88	988.60	9.56	B
102	Mop-Dod-Ehx-Dod-Ehx	989.86	990.81	4.59	C
103	Aet-Dod-Dec-Dod-Dec	961.86	962.87	6.316	C
104	Aet-Dod-Iso-Dod-Iso	905.80	906.40	9.18	B
110	Nme-Smb-Smb-Cpr-Smb-Smb-Apr-Smb-Smb-Cpr-Smb-Smb	1792.93	1794.20	4.22	A
111	Nme-Smb-Smb-Apr-Smb-Smb-Nme-Cpr-Apr-Smb-Smb-Cpr	1699.90	1701.10	3.66	A
112	Nme-Smb-Smb-Cpr-Smb-Dec-Apr-Smb-Dec-Cpr-Smb-Dec	1901.21	1902.50	5.33	A
113	Apr-Smb-Smb-Apr-Smb-Smb-Apr-Smb-Smb	1325.77	1326.70	3.26	A
115	Frm-Dec-Pet-Dec-Pet	818.57	819.30	4.98	A
116	Apr-Smb-Smb-Dec-Smb-Smb-Dec-Smb-Smb	1491.97	1492.80	5.17	A
117	Nme-Pet-Pet-Apr-Pet-Pet-Nme-Cpr-Apr-Pet-Pet-Cpr	1699.90	1700.90	3.43	A
118	Apr-Mme-Mme-Dec-Mme-Mme-Dec-Mme-Mme	1283.94	1285.00	4.36	A
119	Nme-Mme-Mme-Apr-Mme-Mme-Nme-Cpr-Apr-Mme-Mme-Cpr	1491.88	1508.80	2	A
120	Nme-Smb-Smb-Apr-Smb-Smb-Cpr-Smb-Smb-Apr-Smb-Smb	1777.96	1778.90	3.99	A
121	Nme-Smb-Smb-Cpr-Smb-Smb-Cpr-Smb-Smb-Cpr-Smb-Smb	1807.89	1808.50	2.731	C
122	Nme-Smb-Smb-Apr-Smb-Smb-Nme-Cpr-Apr-Smb-Smb-Cpr-Smb-Smb-Apr-Smb-Smb-Cpr	2587.36	2588.47	2.364	C
123	Nhp-Dod-Dod-Dod-Dod	1032.93	1033.60	6.01	A
124	Nmp-Dec-Dec-Dec-Dec	934.82	936.00	5.59	A
125	Mor-Dec-Dec-Dec-Dec	932.80	933.83	5.705	C
126	Nhe-Dec-Dec-Dec-Dec	906.79	907.90	5.49	A
127	Nhp-Pet-Dec-Dec-Dec-Dec	1081.89	1082.90	5.65	A
128	Frm-Dod-Dod-Apr-Dod-Dod	1059.94	1061.00	5.64	A
129	Frm-Dod-Dod-Pet-Apr-Pet-Dod-Dod	1382.11	1383.10	6.23	A
130	Frm-Ehx-Dod-Aet-Dod-Ehx	947.81	948.90	5.12	A
131	Nhp-Dec-Dec-Dec	723.62	724.40	5.16	A

132	Eea-Dec-Ehx-Dec	709.61	710.10	4.92	A
133	Nhp-Nmp-Dec-Dec-Dec	992.86	993.80	5.13	A
134	Nhp-Dec-Ehx-Dec	695.59	696.30	4.73	A
135	Nhe-Dec-Ehx-Dec-Ehx	850.72	851.40	5.14	A
136	Nhe-Dec-Ole-Dec-Ole	1127.01	1128.06	7.475	C
137	Aet-Dec-Ehx-Dec	680.59	681.63	4.453	C
139	Frm-Dod-Dod-Mae-Dod-Dod	1059.94	1060.96	6.614	C
141	Frm-Dod-Pet-Dod-Aet-Dod-Pet-Dod	1368.09	1369.11	6.93	C
144	Nhe-Dod-Dod-Dod-Dod	1018.91	1019.91	6.06	C
145	Dea-Dec-Dec-Dec-Dec	950.81	972.86	5.403	C
146	Frm-Aet-Dec-Dec-Dec-Dec	933.80	934.83	5.239	C
147	Frm-Mae-Dec-Dec-Dec-Dec	947.81	948.84	5.431	C
152	Frm-Dod-Fur-Dod-Aet-Dod-Fur-Dod	1320.02	1321.07	6.406	C
153	Frm-Dod-Pet-Dod-Apr-Dod-Pet-Dod	1382.11	1383.15	6.954	C
155	Frm-Ole-Ole-Ole-Ole	1274.17	1063.98	6.839	C
159	Frm-Dod-Dod-Pet-Aet-Pet-Dod-Dod	1368.09	1369.50	6.13	A
160	Frm-Pet-Dod-Dod-Aet-Dod-Dod-Pet	1368.09	1369.20	6.17	A
161	Frm-Ehx-Pet-Dod-Aet-Dod-Pet-Ehx	1255.97	1256.90	5.72	A
162	Frm-Dod-Pet-Ehx-Aet-Ehx-Pet-Dod	1226.94	1257.00	5.82	A
163	Apr-Ole-Ehx-Ole-Ehx	1097.99	1099.65	5.885	A
164	Nhe-Ole-Ehx-Ole-Ehx	1084.96	1085.00	5.81	A
165	Apr-Ehx-Ole-Ehx-Ole	1083.97	1089.10	5.82	A
166	Aet-Ole-Ole-Ole-Ole	1346.24	1347.03	5.829	A
170	Aet-Ole-Ehx-Ole-Ehx-Ole	1377.25	1378.22	5.86	A
171	Aet-Pet-Ole-Ehx-Ole-Ehx	1231.04	1232.20	5.98	A
172	Frm-Ole-Ehx-Aet-Ole-Ehx	1083.94	1084.74	6.06	A
173	Frm-Dod-Dod-Aet-Aet-Dod-Dod	1160.00	1160.91	5.59	A
174	Pip-Ole-Ehx-Ole-Ehx	1166.05	1167.10	5.658	A
175	Mpi-Ole-Ehx-Ole-Ehx	1138.02	1139.10	5.84	A
176	Mpn-Ole-Ehx-Ole-Ehx	1124.01	1125.20	5.83	A
177	Apd-Ole-Ehx-Ole-Ehx	1100.95	1101.20	5.87	A
178	Imp-Ole-Ehx-Ole-Ehx	1134.99	1136.20	5.93	A
179	Mip-Ole-Ehx-Ole-Ehx	1149.00	1150.10	5.81	A
180	Mop-Ole-Ehx-Ole-Ehx	1154.02	1155.20	5.82	A
181	Mpp-Ole-Ehx-Ole-Ehx	1167.05	1168.21	5.85	A
182	Aet-Ole-Ehx	593.52	594.60	5.58	A
183	Aet-Ole-Ehx-Ole-Ehx-Ole-Ehx	1546.39	1547.40	6.6	A
184	Aet-Ole-Ehx-Ole-Ehx-Ole-Ehx-Ole	1853.68	1855.72	10.319	C
193	Apd-Dec-Dec-Dec-Dec	936.80	937.80	5.332	C
194	Dea-Ole-Ehx-Ole-Ehx	1114.97	1118.00	6.765	C
195	Aad-Ole-Ehx-Ole-Ehx	1100.95	1099.97	6.488	C
198	Frm-Ole-Ehx-Pet-Aet-Pet-Ehx-Ole	1420.12	1421.13	7.299	C
201	Frm-Imp-Ole-Ehx-Ole-Ehx	1162.98	1163.99	7.014	C
202	Frm-Pip-Ole-Ehx-Ole-Ehx	1180.03	1181.07	6.742	C

204	Frm-Mpp-Ole-Ehx-Ole-Ehx	1195.04	1196.08	6.487	C
205	Frm-Aet-Ole-Ehx-Ole-Ehx	1097.95	1099.01	6.804	C
208	Frm-Mip-Ole-Ehx-Ole-Ehx	1177.00	1178.04	6.54	C
210	Frm-Imp-Imp-Ole-Ehx-Ole-Ehx	1328.07	1329.08	6.696	C
211	Frm-Ole-Ehx-Imp-Imp-Ole-Ehx	1328.07	1329.08	6.465	C
212	Frm-Ole-Imp-Ehx-Imp-Ole-Ehx	1328.07	1329.86	4.783	C
213	Frm-Ole-Ehx-Imp-Ole-Ehx	1162.98	1164.70	5.04	C
214	Nhe-Ole-Ehx	594.51	595.53	4.193	C
215	Nhe-Ole-Ehx-Ole	931.81	932.82	6.493	C
217	Nhe-Ole-Ehx-Ole-Ehx-Ole-Ehx	1577.39	1579.40	9.563	C
219	Nhe-Dec-Dec-Dec	739.62	740.6337	4.855	C

Table S9. Sequences and synthetic results for all cationic group variations and lipid block variations synthesized in this work.

Peptoid	Sequence	Yield (mg)	HPLC Retention Time (min)	HPLC Purity	Calculated Exact Mass	Found Exact Mass
222	Aet-Dod-Dod-Dod-Dod	37.5	6.627	87.12	1017.93	1018.93
223	Apr-Dod-Dod-Dod-Dod	61.4	6.602	82.98	1031.94	1032.95
224	Nhe-Dod-Dod-Dod-Dod	36	6.939	90.86	1018.91	1019.92
225	Nhp-Dod-Dod-Dod-Dod	38.3	6.922	94.036	1032.93	1033.93
226	Apd-Dod-Dod-Dod-Dod	n.d	6.799	96.89	1048.92	1049.94
227	Dea-Dod-Dod-Dod-Dod	40.2	6.901	90.83	1062.94	1063.94
228	Eae-Dod-Dod-Dod-Dod	28	6.606	94.12	1046.94	1101.01
229	Nme-Dod-Dod-Dod-Dod	45.5	6.96	87.24	1032.93	1033.94
230	Nmp-Dod-Dod-Dod-Dod	21.8	6.971	99.09	1046.94	1047.95
231	Dme-Dod-Dod-Dod-Dod	49.2	6.579	81.18	1045.96	1048.10
232	Mip-Dod-Dod-Dod-Dod	28.2	6.437	87.94	1096.97	1116.02
233	Mop-Dod-Dod-Dod-Dod	7	6.438	98.5	1101.98	1102.98
234	Apd-Dec-Dec-Dec-Dec	n.d	6.096	96.62	936.80	937.80
235	Apd-Dod-Ehx-Dod-Dod-Ehx-Dod	97.2	9.686	90.41	1387.22	1388.22
236	Apd-Ehx-Dod-Ehx-Ehx-Dod-Ehx	59.8	7.359	93.88	1275.09	1276.10
237	Apd-Dec-Ehx-Dec-Dec-Ehx-Dec	111.8	7.039	91.31	1275.09	1276.10
238	Apd-Dod-Ole-Dod	22.2	6.478	89.1	905.79	906.80
239	Apd-Ole-Dec-Ole-Dec	n.d	7.499	63.81	1157.02	1158.02
240	Apd-Ole-Dec-Ole	48.8	6.837	81.18	959.84	960.85
241	Apd-Ehx-Dec-Dec-Dec-Ehx-Dec-Dec-Dec	87.2	8.163	91.07	1669.45	1671.46
242	Apd-Ole-Hex-Hex-Hex-Ole-Hex-Hex-Hex	69.6	7.804	53.34	1609.35	1611.36
243	Apd-Oct-Hex-Hex-Hex-Hex-Hex-Hex-Oct	55.6	6.042	92.79	1333.07	1334.08
244	Apd-Ehx-Oct-Oct-Oct-Ehx-Oct-Oct-Oct	32.8	7.103	93.66	1501.26	1502.26
245	Apd-Ehx-Ehx-Hex-Ehx-Ehx-Hex	48.2	5.899	96.49	1106.90	1107.91
246	Apd-Ehx-Hex-Ehx-Hex-Ehx-Hex-Ehx-Hex	25.2	6.455	93.19	1389.13	1390.14
247	Apd-Hex-Hex-Hex-Hex-Hex-Hex-Hex-Hex	58.6	5.7	92.22	1277.01	1278.02
248	Apd-Ehx-Dod-Hex-Hex-Ehx-Dod-Hex-Hex	33	7.33	93.4	1501.26	1202.06
249	Apd-Dod-Dod-Dod-Dod-Dod-Dod	n.d	8.396	87.77	1499.34	1501.37
250	Apd-Ehx-Dod-Dod-Hex-Ehx-Dod-Dod-Hex	63.4	8.186	95.93	1669.45	1671.82
251	Apd-Ole-Oct-Ole	n.d	6.583	68.5	931.81	932.82
252	Apd-Dod-Dec-Dod-Dec	25.2	6.389	98.4	992.86	993.86
253	Apd-Dod-Ehx-Dod-Ehx	64.3	6.121	100	936.80	1107.91
254	Apd-Ehx-Ehx-Ehx-Ehx	59.4	5.278	100	824.67	825.68
255	Apd-Oct-Oct-Hex-Oct-Oct-Hex	62.9	5.815	85.35	1106.90	1107.91
256	Apd-Oct-Oct-Dec-Oct-Oct-Dec	16.1	5.891	95.3	1219.03	1220.04
257	Apd-Dec-Dod-Dec-Dec-Dod-Dec	n.d	7.651	80.69	1387.22	1388.23
258	Apd-Oct-Dec-Ehx-Oct-Dec-Ehx	62.4	6.587	96.01	1219.03	1220.04
259	Apd-Dec-Dec-Ehx-Dec-Dec-Ehx	65.4	6.943	99.64	1275.09	1276.10

260	Apd-Dod-Dod-Ehx-Dod-Dod-Ehx	49.3	7.73	94.48	1387.22	1388.23
261	Apd-Oct-Dec-Hex-Oct-Dec-Hex	n.d	6.179	60.41	1162.96	1163.98
262	Apd-Dod-Dod-Oct-Dod-Dod-Oct	14.1	7.743	91.21	1387.22	2.00
263	Apd-Hex-Dod-Ehx-Hex-Dod-Ehx	n.d	6.622	94.12	1219.03	1220.04
264	Apd-Oct-Dod-Ehx-Oct-Dod-Ehx	n.d	6.977	93.49	1275.09	1276.11
265	Apd-Ole-Oct-Ole-Oct	n.d	7.048	62.25	1100.95	1101.97
266	Apd-Dod-Ehx-Ehx-Dod-Ehx-Ehx	60.8	7.052	97.52	1275.09	1276.11

Representative Peptoid LC-MS Traces and Spectra:

Peptoid **3** – Cluster 1

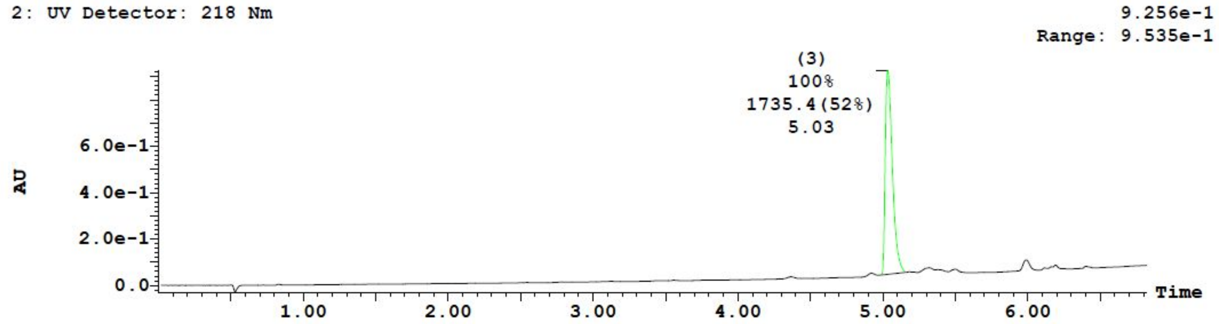

Peak Number	Time	AreaAbs	Area %Total	Height	Mass Found
3	5.03	5.03e+004	100.00	8.77e+005	1735.3700

Peak ID	Time	Mass Found
3	5.04	1758.86,579.59,868.29,1736.20

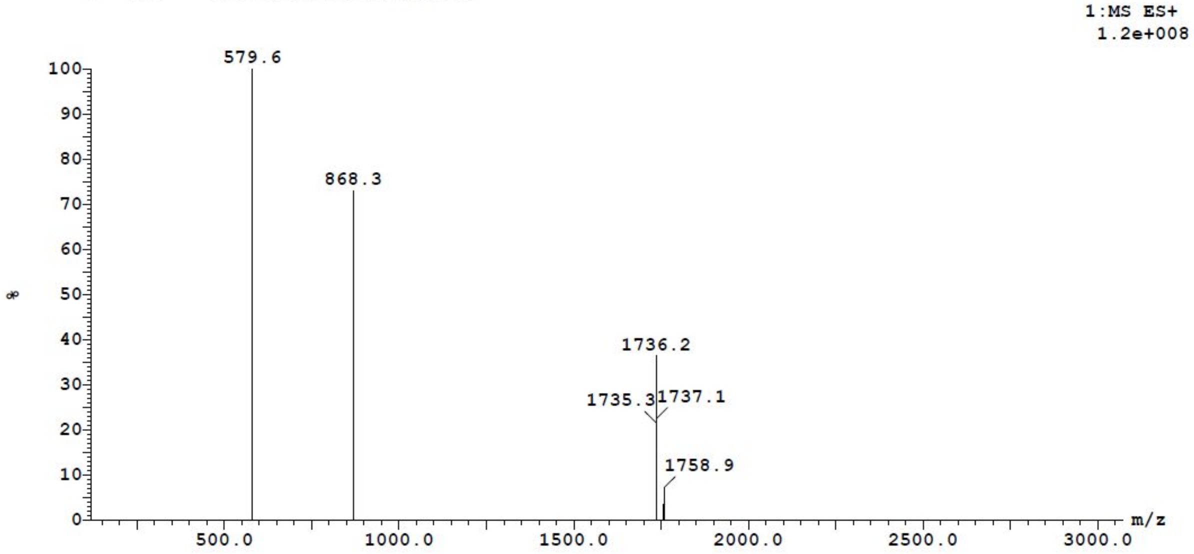

Peptoid **8** – Cluster 3

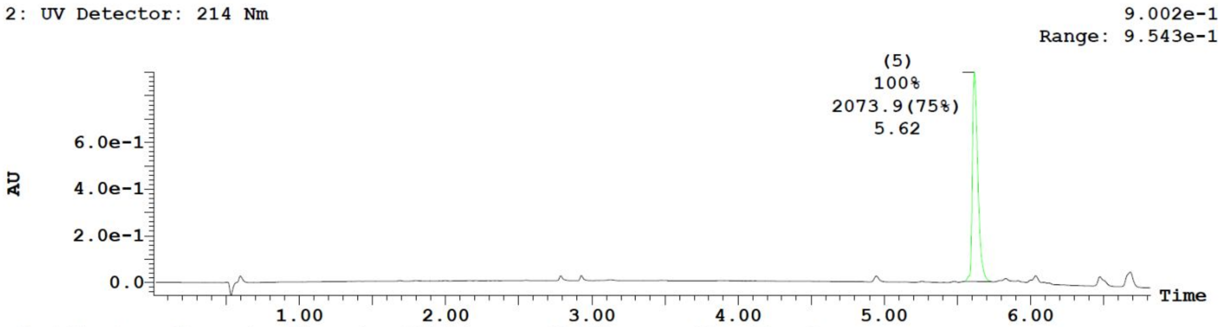

Peak Number	Time	AreaAbs	Area %Total	Height	Mass Found
5	5.62	3.91e+004	100.00	8.95e+005	2073.9099

Peak ID	Time	Mass Found
5	5.62	692.21,1037.81,2075.30

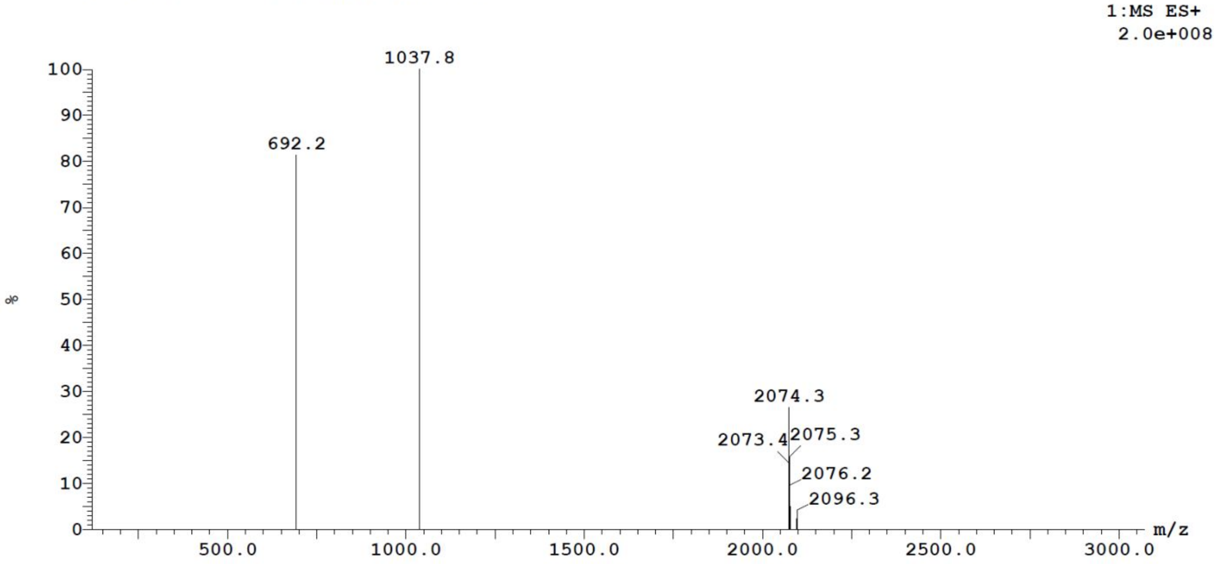

Peptoid **12** – Cluster 4

Sample 1 Vial 9:38 File CJM_20190815_NTX-DVI-0012 Date 15-Aug-2019 Time 19:59:48 Description NTX-DVI-0012

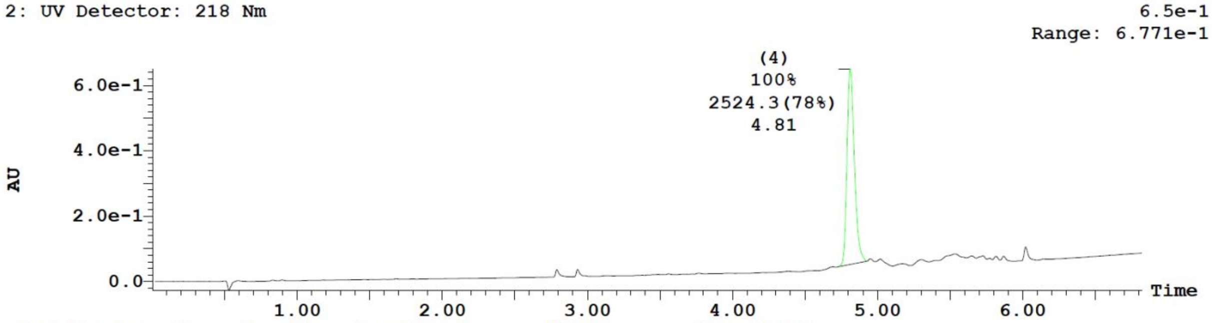

Peak Number	Time	AreaAbs	Area %Total	Height	Mass Found
4	4.81	3.56e+004	100.00	5.98e+005	2524.3201

Peak ID	Time	Mass Found
4	4.82	632.14,842.46,1263.41,2525.57

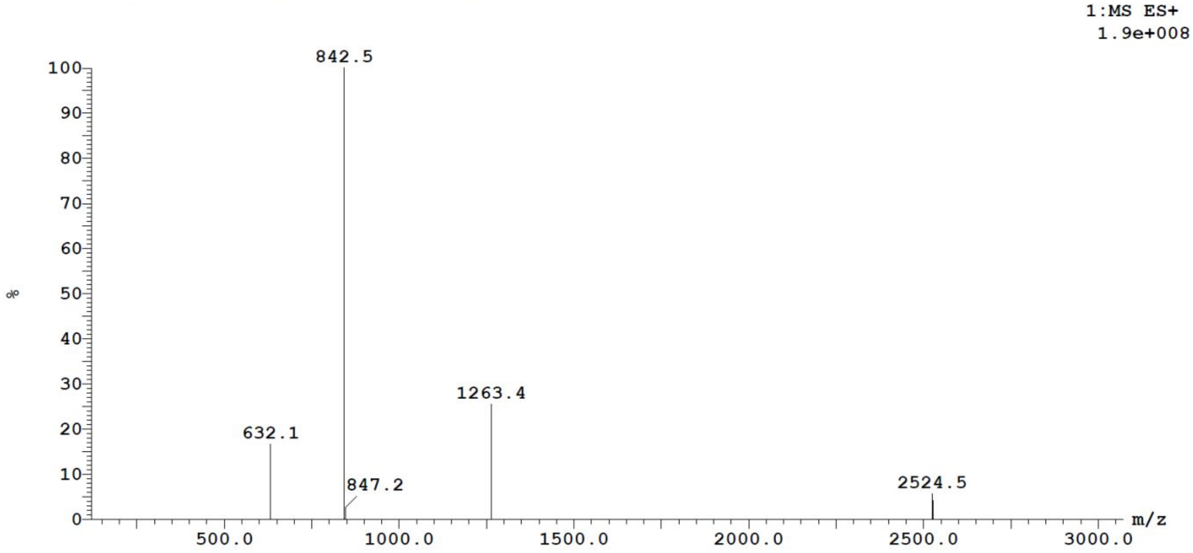

Peptoid **40** - Cluster 8

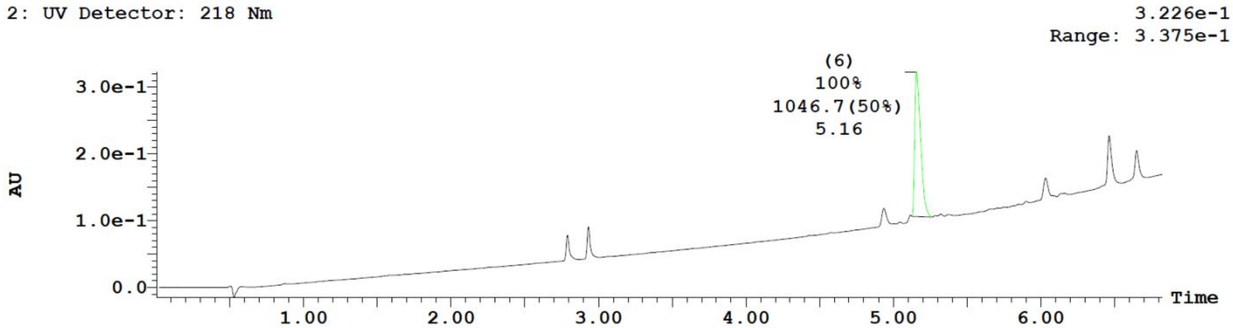

Peak Number	Time	AreaAbs	Area %Total	Height	Mass Found
6	5.16	1.01e+004	100.00	2.16e+005	1046.6700

Peak ID	Time	Mass Found
6	5.17	1070.06,524.16,1047.70

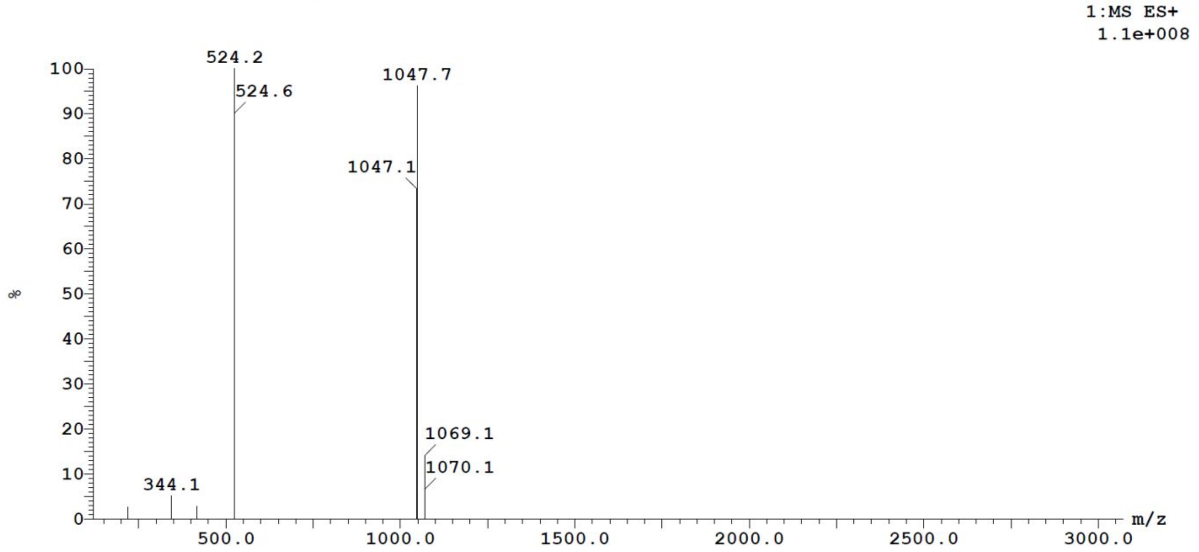

Peptoid **72/178** - Cluster 9

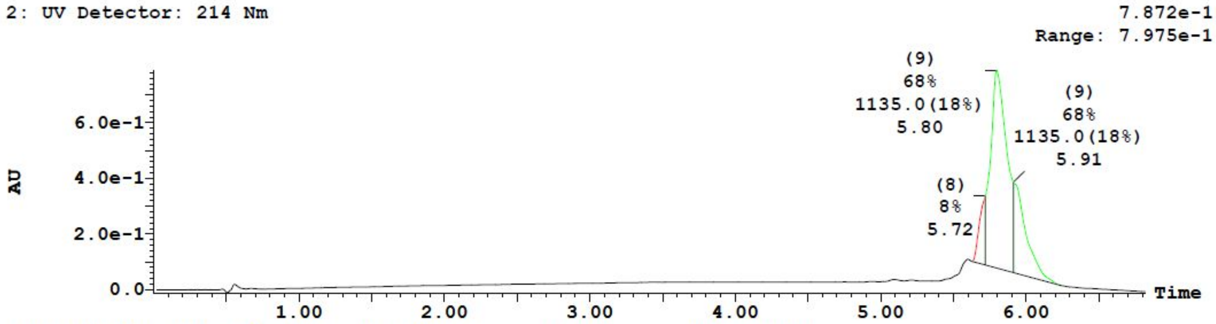

Peak Number	Time	AreaAbs	Area %Total	Height	Mass Found
8	5.72	1.11e+004	8.09	2.47e+005	Not Found
9	5.80	9.30e+004	67.99	7.09e+005	1134.99
10	5.91	3.27e+004	23.91	3.24e+005	1134.99

Peak ID	Time	Mass Found
9	5.81	1158.17,568.70,1136.18

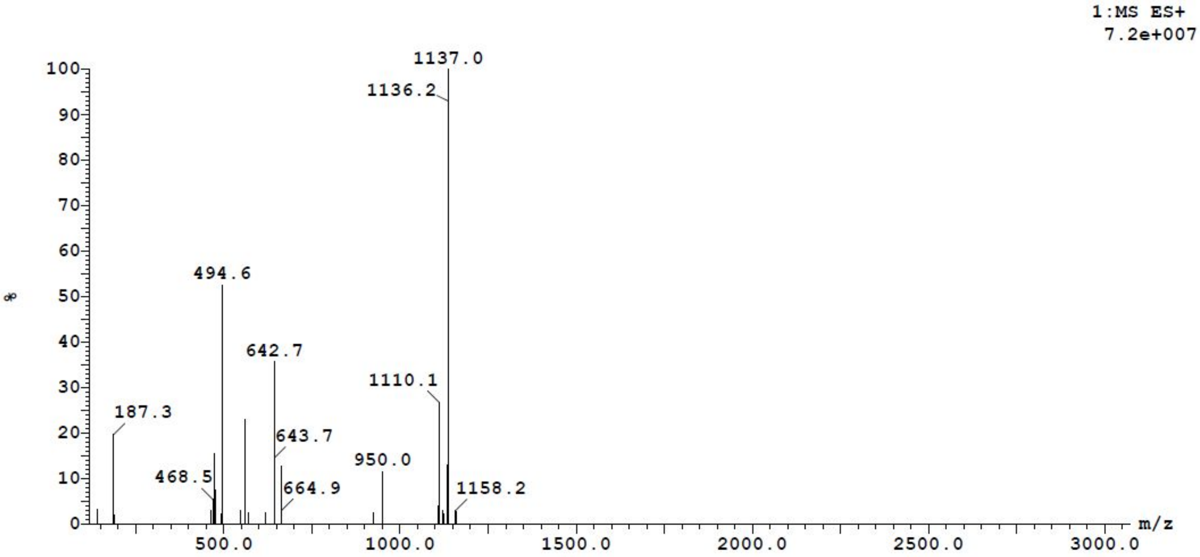

Peak ID	Time	Mass Found
10	5.93	1157.73,568.77,1136.18
